# Heterocyclic Compounds as Synthetic Tyrosinase Inhibitors: Recent Advances

**DOI:** 10.3390/ijms24109097

**Published:** 2023-05-22

**Authors:** Serena Vittorio, Christian Dank, Laura Ielo

**Affiliations:** 1 Dipartimento di Scienze Farmaceutiche, Università degli Studi di Milano, Via Mangiagalli, 25, 20133 Milano, Italy; serena.vittorio@unimi.it; 2 Institute of Organic Chemistry, University of Vienna, Währinger Strasse 38, 1090 Vienna, Austria; christian.dank@univie.ac.at; 3 Department of Chemistry, University of Turin, Via P. Giuria 7, 10125 Torino, Italy

**Keywords:** tyrosinase, heterocycles, mushroom tyrosinase inhibitors, human tyrosinase inhibitors

## Abstract

Tyrosinase is a copper-containing enzyme which is widely distributed in nature (e.g., bacteria, mammals, fungi) and involved in two consecutive steps of melanin biosynthesis. In humans, an excessive production of melanin can determine hyperpigmentation disorders as well as neurodegenerative processes in Parkinson’s disease. The development of molecules able to inhibit the high activity of the enzyme remain a current topic in medicinal chemistry, because the inhibitors reported so far present several side effects. Heterocycle-bearing molecules are largely diffuse in this sense. Due to their importance as biologically active compounds, we decided to report a comprehensive review of synthetic tyrosinase inhibitors possessing heterocyclic moieties reported within the last five years. For the reader’s convenience, we classified them as inhibitors of mushroom tyrosinase (*Agaricus bisporus*) and human tyrosinase.

## 1. Introduction

**Figure 1:**
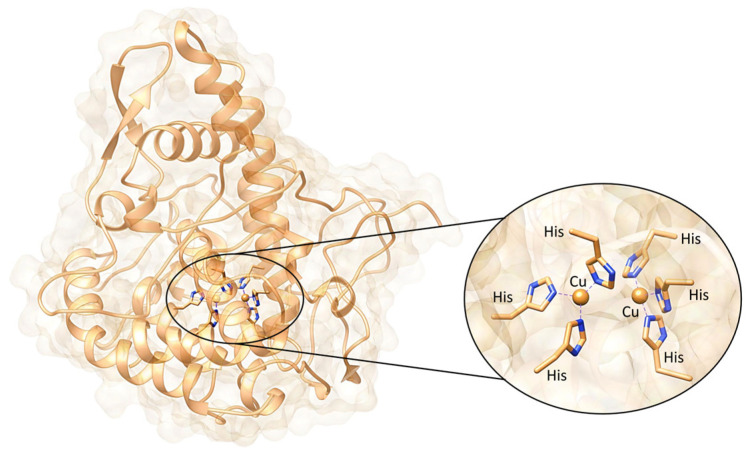
Three-dimensional structure of TYR with a close view on the active site of the enzyme.

Tyrosinase (TYR) (EC 1.14.18.1) is a binuclear copper-containing protein widely distributed in nature (e.g., bacteria, mammals, fungi). The structure of the enzyme can be divided into three parts: *N*-terminal domain, central domain, and *C*-terminal domain. The central domain, that represents the catalytic site, is characterized by six conserved histidine residues, coordinating two copper-oxidizing ions (Figure 1) [1]. 

**Scheme 1:**
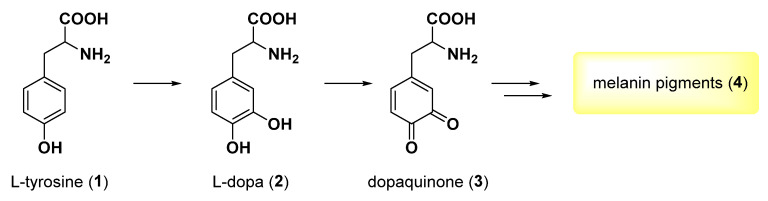
Biosynthesis of melanin (**4**).

TYR is involved in the biosynthesis of melanin. In particular, it catalyzes the oxidation of l-tyrosine (**1**) to l-dopa (**2**) and the subsequent oxidation of l-dopa (**2**) to dopaquinone (**3**). The first activity is called tyrosine hydroxylase (monophenolase activity) and the second one is o-diphenol oxidase, catechol oxidase or DOPA oxidase (diphenolase activity). Once dopaquinone (**3**) is formed, the pathway progresses through a series of spontaneous reactions leading to the final melanin (**4**) pigments (Scheme 1) [2]. 

Melanin (**4**) has a crucial role in skin, hair, and eye pigmentation, and in skin protection from UV radiation. For this reason, an excessive production of melanin can lead to hyperpigmentation disorders. Furthermore, the accumulation of melanin in the substantia nigra of human neurons seems related to neurodegeneration in Parkinson’s disease [3]. Thus, it is important to mitigate the excessive activity of TYR. 

In recent years, several tyrosinase inhibitors (TYRIs) from natural [4] and synthetic [5,6] sources have been reported. However, the TYRIs currently employed in hyperpigmentation disorders present various side effects and the development of molecules with better pharmacological characteristics is still needed [7]. 

Heterocycles are cyclic structures containing one or more heteroatoms. The most recurring ones bear nitrogen, oxygen, or sulfur, but rings containing phosphorus, magnesium, selenium, and others can be found as well [8]. Heterocycles constitute privileged scaffolds as they are building blocks of many natural molecules such as DNA, RNA, proteins, vitamins, the heme group, and chlorophyll, which are essential for physiological cellular functions [9]. Due to their important biological roles, several efforts have been made towards the development of new synthetic strategies which allow access to and functionalization of heterocyclic molecules [10,11,12,13,14,15,16]. Moreover, some heterocyclic compounds were isolated from natural sources and their structures were opportunely modified [17]. It was estimated that more than 90% of new drugs present a heterocyclic moiety [9]. Heterocycles are widely applied in medicinal chemistry as they enable the formation of hydrogen bonds, and the modification of the solubility, lipophilicity, and polarity, thus facilitating the optimization of the ADMET profile of novel drug candidates [18]. 

For this reason, we decided to report a comprehensive review of the recent advances (last five years) of TYRIs bearing heterocycles. In particular, we organized them by taking into consideration the heterocyclic moiety and the inhibition towards the tyrosinase from the mushroom *Agaricus bisporus* (AbTYR) or the human tyrosinase (hTYR). This work is intended to be useful and inspiring for the scientific community in order to collect new SAR information for future developments of novel TYRIs with better pharmacological characteristics. The IC_50_ values or PI of all the compounds described in the manuscript are reported as Supplementary Material File.

## 2. Mushroom Tyrosinase Inhibitors

**Figure 2:**
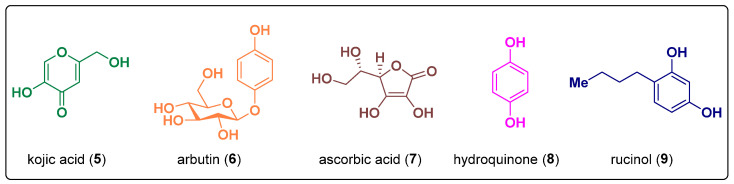
Common TYRIs, typically used as positive controls in determining inhibitory activities.

AbTYR [19] is usually employed for determining the inhibitory activity of TYRIs [20]. Although several differences exist between human and mushroom isoforms, the usage of the commercially available AbTYR allows a simple screening of new plausible TYRIs. Several hit compounds, detected via AbTYR, also proved to be able to inhibit hTYR-enabling anti-melanogenic effects in human cell lines, although they resulted in less potent agents [7]. Kojic acid (**5**), arbutin (**6**), ascorbic acid (**7**), hydroquinone (**8**), and rucinol (**9**) are well-known whitening agents and are often used as positive controls in the in vitro assays for determining anti-tyrosinase activity (Figure 2) [21].

### 2.1. Carbazoles

**Figure 3:**
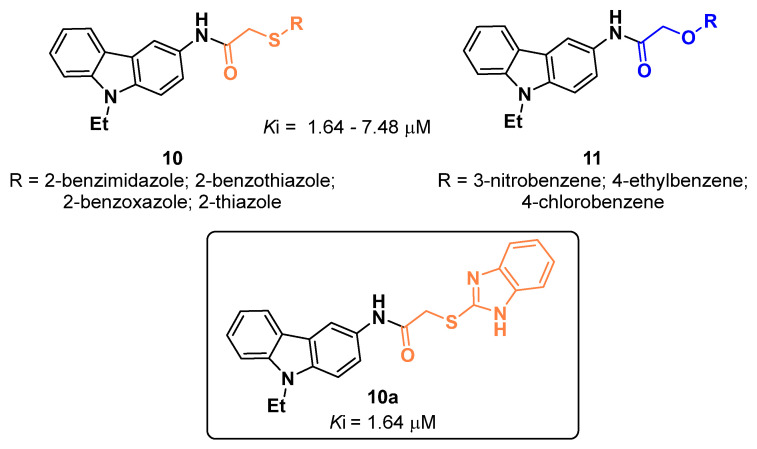
TYRIs bearing the carbazole scaffold.

In 2019, Ghani described the anti-tyrosinase activity of seven carbazole derivatives bearing different aromatic and heteroaromatic moieties (Figure 3) [22]. These compounds (**10**–**11**, Figure 3) inhibited the diphenolase activity in a competitive manner, displaying *K*_i_ values between 1.64 and 7.48 μM, thus showing similar or better potency than kojic acid (**5**, *K*i = 4.43 µM), which was used as a reference. The most active compound **10a** carried a benzimidazole group and, according to the copper binding assay, it might inhibit TYR activity by interacting with the binuclear copper center. 

### 2.2. Compounds Derived from Pyrrol

**Figure 4:**
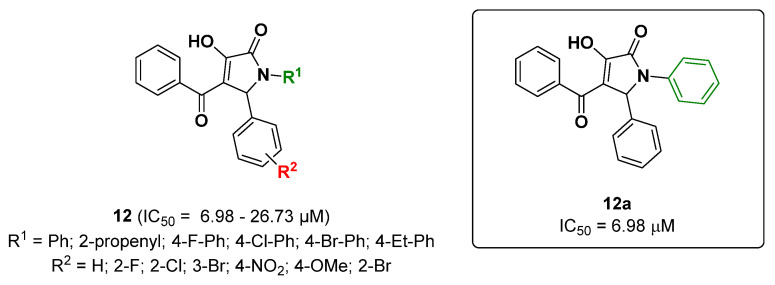
3-Hydroxy-1*H*-pyrrol-2(5*H*)-one derivatives and their inhibitory activity.

In 2022, Mahdavi and co-workers described a series of disubstituted 3-hydroxy-1*H*-pyrrol-2(5*H*)-one derivatives (**12**, Figure 4) as TYRIs [23]. Their inhibitory activity was evaluated on diphenolase activity obtaining IC_50_ values between 6.98 and 26.73 µM, compared to kojic acid (**5**, IC_50_ = 18.56 µM). The most active compound **12a**, with an IC_50_ value of 6.98 µM, is a mixed-type inhibitor. SAR evaluation showed that H or small EWGs in the *ortho* position of R^2^ are favorable when R^1^ is a phenyl group. When a propenyl is present, however, only small EWGs in the *ortho* position of R^2^ improved the activity. In general, phenyl was the most favorable substituent in the R^1^ diversity site.

**Figure 5:**
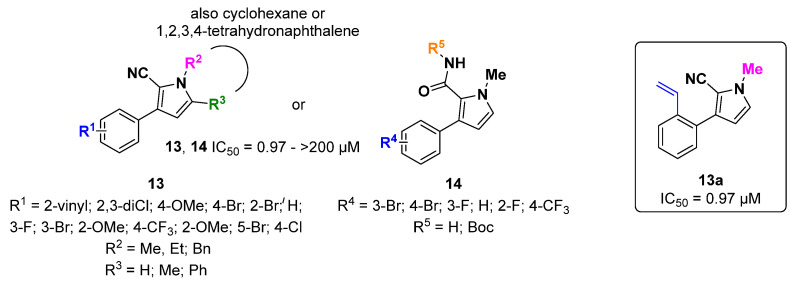
Derivatives of 2-cyanopyrrole and their inhibitory activities.

Zhu et al. synthesized a series of 2-cyanopyrrole derivatives (**13**–**14**, Figure 5) as TYRIs [24]. IC_50_ values from 0.97 to >200 µM were shown on diphenolase activity, compared to kojic acid (**5**, IC_50_ = 28.72 µM). The best result was displayed by derivative **13a** with an IC_50_ value of 0.97 µM, which is a reversible mixed-type inhibitor (Figure 5). It also showed a good inhibitory effect on TYR in B16 melanoma cells (inhibition of 33.48%), which was almost equivalent to kojic acid (**5**, 39.81%).

### 2.3. Indole Derivatives

**Figure 6:**
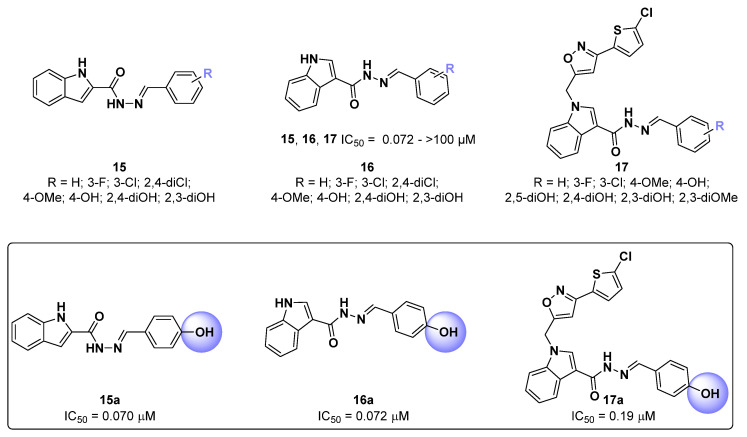
Indole-carbohydrazide derivatives.

In 2022, Mirfazli et al. described a new series of indole derivatives (**15**–**17**, Figure 6) as TYRIs [25]. The 25 indole-carbohydrazide derivatives (**15**–**17**, Figure 6), linked to different aryl substituents, were synthesized and their biological activity was evaluated by using l-dopa (**2**) as a substrate showing an IC_50_ value between 0.072 and >100 µM. Three different classes were designed and the best activity for each class was displayed by the *para* hydroxy-substituted compounds (**15a**, **16a**, and **17a**) with IC_50_ values of 0.070, 0.072, and 0.19 µM, respectively, compared to the positive control kojic acid (**5**, IC_50_ = 9.28 µM). The three most active compounds **15a**, **16a**, and **17a** showed a mixed-type inhibition and did not show cytotoxicity at a concentration of 8 μM. Moreover, they were able to reduce the percentage of melanin content to 68.43%, 72.61%, and 73.47%, respectively. Considering SAR analysis, the presence of the chlorothiophene-isoxazole moiety improved the activity, especially when R is an electron-donating group (EDG). The *para* hydroxy phenyl substitution significantly improved the activity (Figure 6).

**Figure 7:**
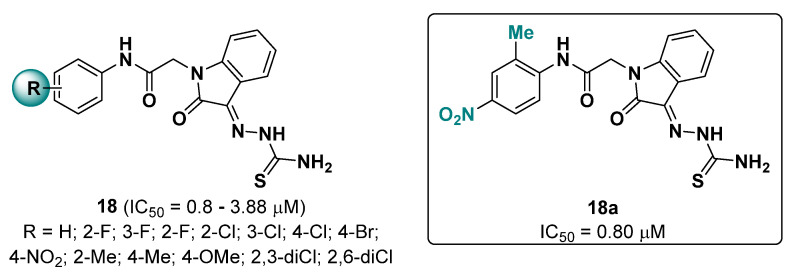
*N*-Phenylacetamide-oxindole-thiosemicarbazide hybrids (**18**) and their activities.

Mahdavi et al. reported a series of *N*-phenylacetamide-oxindole-thiosemicarbazide hybrids (**18**, Figure 7) as TYRIs [26]. All the prepared compounds (**18**, Figure 7) showed TYR inhibitory activity by using l-dopa (**2**) as substrate with IC_50_ values between 0.8 and 3.88 μM, compared to kojic acid (**5**, IC_50_ = 36.32 μM). The most promising compound, **18a**, with an IC_50_ value of 0.8 μM, possesses a 2-methyl-4-nitrophenyl group on the *N*-phenylacetamide moiety (Figure 7). The authors identified **18a** as a competitive inhibitor and docking studies suggested that it interacts with histidine groups with crucial roles in the catalytic pocket. 

### 2.4. Pyrazoles

**Figure 8:**
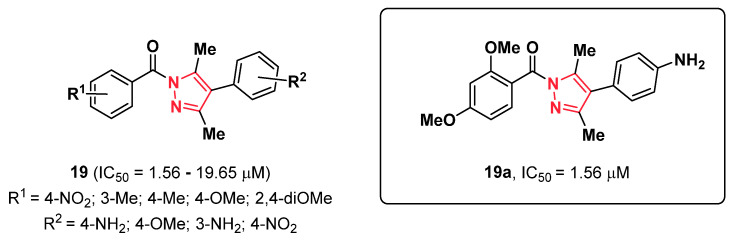
TYRIs bearing an aryl pyrazole moiety.

In 2018, Channar and co-workers reported the anti-tyrosinase activity of a series of aryl pyrazole derivatives (**19**, Figure 8) which inhibited the diphenolase activity with IC_50_ values in the range of 1.56 to 19.65 μM [27]. Among the tested compounds, the most effective inhibitor was derivative **19a** (IC_50_ = 1.56 μM, shown in Figure 8) which was found to be about tenfold more active than kojic acid (**5**, IC_50_ = 16.05 μM). Kinetic analysis revealed that **19a** was a non-competitive inhibitor of TYR. Docking studies suggested that **19a** might occupy the enzyme active site with the 4-methoxy group oriented towards the metal ions. 

**Figure 9:**
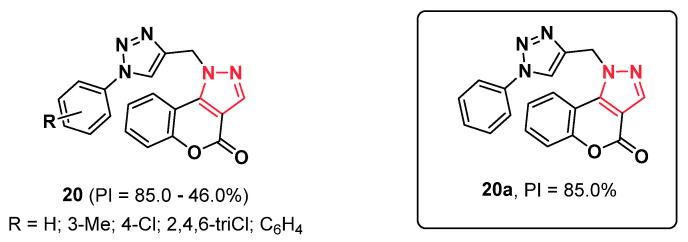
Inhibitory effects of 1,2,3-triazolo-coumarino[4,3-*c*]pyrazole derivatives.

Also in 2018, Chekir et al. reported a class of compounds containing three different heterocyclic moieties, which are individually known for their applications in medicinal chemistry: coumarin, pyrazole, and triazole [28]. The resulting 1,2,3-triazolo-coumarino[4,3-*c*]pyrazoles (**20**, Figure 9) were screened against different enzymes including TYR, by using l-dopa (**2**) as a substrate. The results indicated that these compounds blocked the enzyme activity with percentages of inhibition (PIs) from 85.0 to 46.0%. The most effective inhibitor **20a** showed a PI of 85.0% (Figure 9) thus displaying a comparable anti-tyrosinase activity to that of kojic acid (**5**, PI = 85.2%).

**Figure 10:**
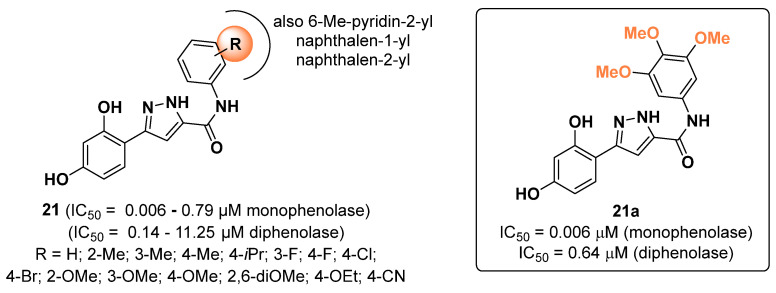
Pyrazole-resorcinol derivatives with promising biological profiles.

Sun and co-workers identified a series of pyrazole-resorcinol derivatives (**21**, Figure 10) as potent TYRIs [29]. The biological inhibition was evaluated both on monophenolase and diphenolase activities obtaining IC_50_ values between 0.006 and 0.79 µM, and 0.14 and 11.25 µM, respectively, compared to kojic acid (**5**, IC_50_ = 21.58 µM monophenolase; IC_50_ = 17.76 µM diphenolase). The most promising results were obtained for derivative **21a** which possesses an IC_50_ value of 0.006 and 0.64 µM on monophenolase and diphenolase activities, respectively, as illustrated in Figure 10. It proved to be a promising inhibitor of TYR, melanogenesis, oxidative stress, and has great potential to be employed as an anti-Parkinsonian drug. Indeed, it can: (a) inhibit melanogenesis in a dose-dependent manner; and (b) scavenge the 2,2-diphenyl-1-picrylhydrazyl (DPPH) free radical and reduce the overproduction of LPS-induced reactive oxidative species (ROS), demonstrating its antioxidative profile. It also showed an excellent neuroprotective effect in vitro and in vivo.

### 2.5. Benzimidazoles

**Figure 11:**
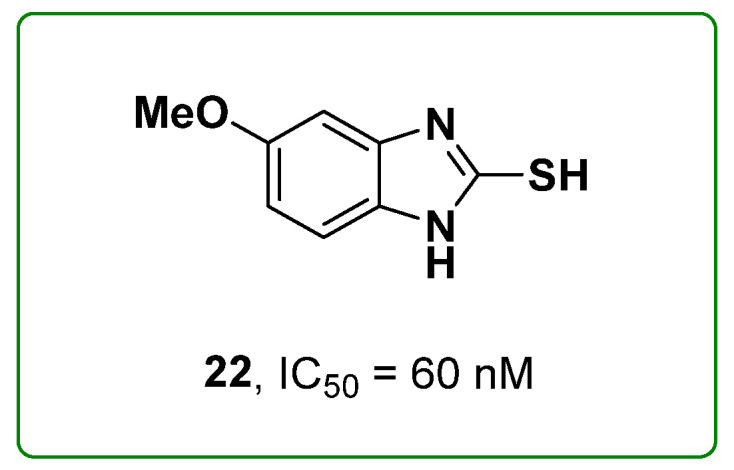
Chemical structure of 5-methoxy-2-mercaptobenzimidazole (**22**).

A good inhibitory activity was observed for 5-methoxy-2-mercaptobenzimidazole (**22**, Figure 11) by Song et al. in 2021 as a reversible and competitive inhibitor of diphenolase activity [30]. It possesses an IC_50_ value of 60 nM which is a promising activity in comparison to arbutin (**6**, IC_50_ = 0.48 mM) and kojic acid (**5**, IC_50_ = 22.16 µM). Molecular docking showed hydrogen bonds and hydrophobic interactions with specific amino acids residues in the A chain of TYR. 

### 2.6. Triazoles

**Figure 12:**
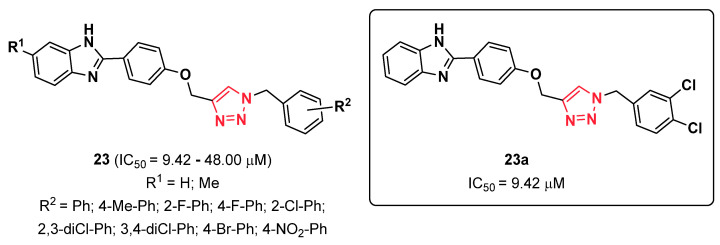
Benzimidazole-1,2,3-triazole hybrids.

Triazoles are considered to be an interesting pharmacophore for developing new bioactive compounds due to their ability to form a wide spectrum of interactions with biological macromolecules, such as pi-stacking, pi-cation, hydrophobic, van der Waals, H-bonds, and coordinative bonds. Moreover, triazole represents a useful linker to join different scaffolds allowing them to obtain bifunctional chemical entities [31,32,33]. Due to its versatility, triazole has also been exploited for the design of new TYRIs. In 2018, Mahdavi and co-workers reported a new class of anti-tyrosinase agents in which the 1,2,3-triazole was combined with the benzimidazole ring [34]. As a result, eighteen derivatives (**23**, Figure 12) were synthesized and tested on the diphenolase activity. Six out of eighteen compounds inhibited TYR with IC_50_ values ranging from 9.42 to 48.00 μM. The inhibition of the most potent compound **23a** (IC_50_ = 9.42 μM) was comparable to that of the reference kojic acid (**5**, IC_50_ = 9.28 μM) (Figure 12). Overall, the biological data pointed out that the presence of the methyl group at the C-6 position of the benzimidazole is unfavorable for activity, while the introduction of halogens in the *para* position of the phenyl ring linked to the triazole portion positively affected the affinity towards TYR. The analysis of the Lineweaver–Burk double reciprocal plots revealed that **23a** acted as mixed-type inhibitor. 

**Figure 13:**
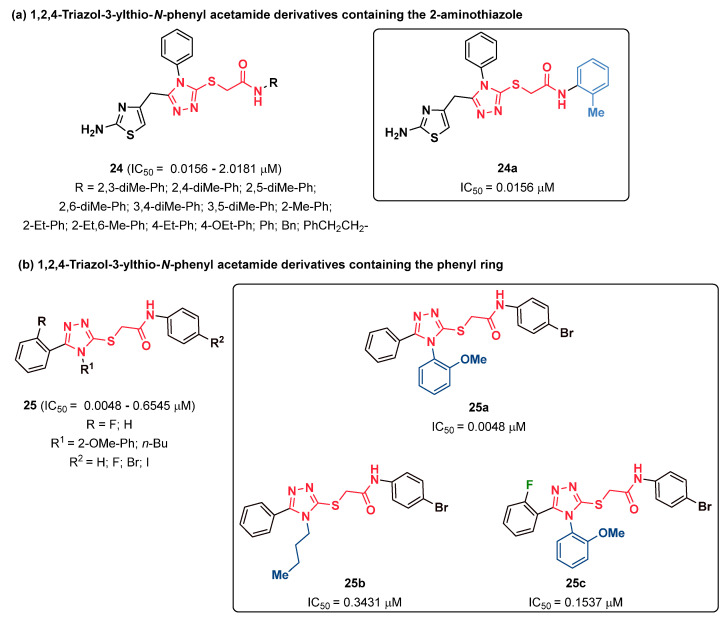
1,2,4-Triazol-3-ylthio-*N*-phenyl acetamide derivatives containing the 2-aminothiazole moiety (**24**) (**a**) and phenyl ring (**25**) (**b**) in the position five of the triazole ring.

In 2019, Butt et al. described the anti-tyrosinase activity of some bi-heterocyclic *N*-substituted acetamides designed by combining the 2-aminothiazole with the 4-phenyl-1,2,4-triazole (**24**, Figure 13a) [35]. All of the compounds exhibited a good inhibitory activity against the diphenolase activity (IC_50_ = 0.0156–2.0181 μM) showing a stronger inhibition than kojic acid (**5**, IC_50_ = 16.832 μM). SAR analysis highlighted that the presence of small substituents in the *ortho* position(s) of the phenylacetamide part generally led to more effective inhibitors. This is illustrated by the most potent derivative **24a**, whose IC_50_ value was 0.0156 μM. Moreover, the introduction of a small flexible linker between the phenyl moiety and the acetamide group also enhanced the affinity towards AbTYR. Kinetic studies showed that **24a** competitively inhibited the enzyme activity. The binding mode of **24a** was investigated in silico, revealing that the two phenyl rings might establish pi-stacking interactions with His259, His85, and His244. Instead, the 2-aminothiazole portion might be involved in an H-bond with Cys83 and aromatic interactions with His85. Another series of TYRIs bearing the 1,2,4-triazol-3-ylthio-*N*-phenyl acetamide was reported in 2020 by the same research group [36]. In vitro screening against AbTYR, by using l-dopa (**2**) as a substrate, highlighted that all of the synthesized compounds (**25**, Figure 13b) effectively inhibited the enzyme, with IC_50_ values ranging from 0.0048 to 0.6545 μM, thus displaying a higher potency than the standard kojic acid (**5**, IC_50_ = 16.832 μM). The most active compound (**25a**) carried a 4-bromophenyl moiety, connected to the acetamide group and the 2-methoxyphenyl group linked to the nitrogen at position four of the triazole and displayed an IC_50_ value of 0.0048 μM. Overall, it was observed that the derivatives bearing the 2-methoxyphenyl ring were more active than the aliphatic-substituted analogs, such as compound **25b**. Moreover, except for the iodo-derivative, the presence of a fluorine atom in the *ortho* position of the phenyl moiety connected to the triazole ring, as in **25c**, generally led to a reduction of the inhibitory effect if compared to the non-fluorinated analogs.

**Figure 14:**
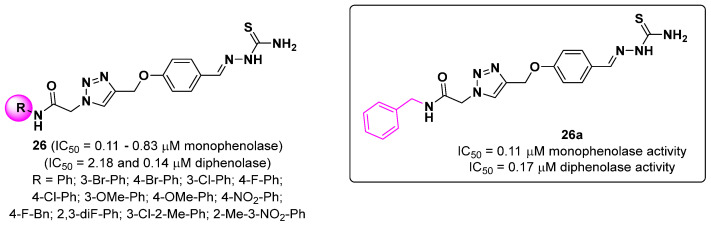
Aryl phenoxy methyl triazoles conjugated with thiosemicarbazides derivatives.

A variety of derivatives possessing thiosemicarbazide functionality were described as TYRIs [37,38,39] and anti-browning agents [40]. Among them Khoshneviszadeh et al. identified a new class of aryl phenoxy methyl triazole conjugated with thiosemicarbazide derivatives (**26**, Figure 14) as TYRIs [41]. All the compounds showed a good activity with IC_50_ values between 0.11 and 0.83 μM using l-tyrosine (**1**) as a substrate, and between 2.18 and 0.14 μM using l-dopa (**2**) as a substrate compared to the positive control kojic acid (**5**, IC_50_ = 9.30 μM). The best result was obtained for compound **26a** with an IC_50_ value of 0.11 μM for monophenolase activity and 0.17 μM for the diphenolase activity. In addition, **26a** appears to be an uncompetitive inhibitor able to significantly reduce the melanin content of B16F10 melanoma human cells (Figure 14). SAR analysis revealed contrasting results for mono- and diphenolase activities.

**Figure 15:**
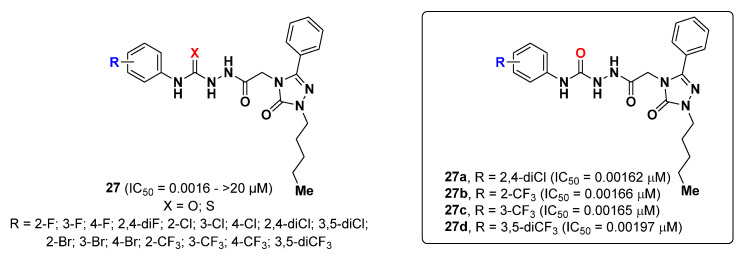
1,2,4-Triazole-thiosemicarbazide hybrid molecules.

Akdemir and co-workers synthesized a new class of 1,2,4-triazole-thiosemicarbazide hybrid molecules (**27**, Figure 15) as TYRIs [42]. The activity of 32 compounds was evaluated on diphenolase activity with IC_50_ values from 0.0016 to >20 µM compared to kojic acid (**5**, IC_50_ = 14.09 µM). Derivatives **27a**–**27d** showed the best activity with IC_50_ values of 0.00162, 0.00166, 0.00165, and 0.00197 µM, respectively (Figure 15). The main interactions of these compounds within the catalytic site of the enzyme were evaluated via molecular docking. The carbonyl group makes a hydrogen bond with the backbone of Val283 and the thiocarbazone nitrogen atoms with the side chain of Ser282. The phenyl group can be directed towards either Phe264 or Val283.

**Figure 16:**
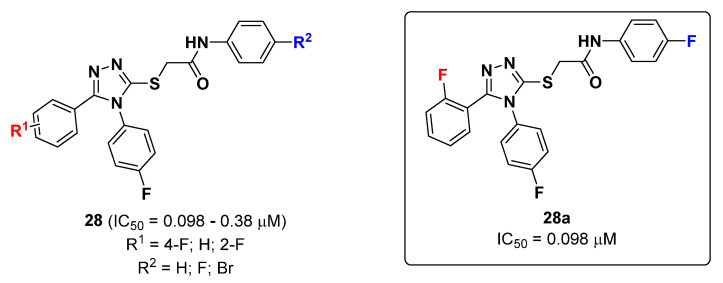
1,2,4-Triazole based compounds (**28**) and their inhibitory activities.

In 2022, Kloczkowski and co-workers identified a series of 1,2,4-triazole based compounds (**28**, Figure 16) as TYRIs [43]. Good activities were observed for almost all of the derivatives, as IC_50_ values were between 0.098 and 0.38 μM on diphenolase activity, compared to the positive control kojic acid (**5**, IC_50_ = 16.83 μM). Compound **28a**, showing an IC_50_ value of 0.098 μM, is a competitive inhibitor and proved to be a good lead compound for the design of further molecules with improved pharmacological characteristics (Figure 16). 

**Figure 17:**
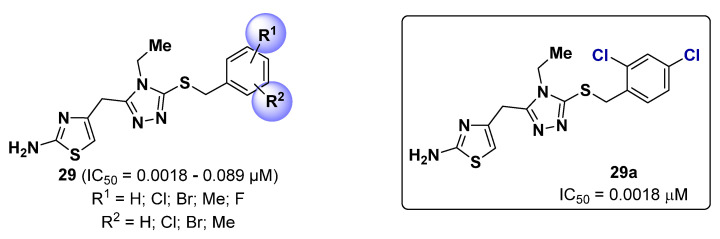
Thiazole–triazole hybrids as TYRIs.

Triazole [44] and thiazole [45,46] derivatives were largely described in the literature as TYRIs. For this reason, Seo and co-workers decided to design a new class of thiazole–triazole hybrid molecules (**29**, Figure 17) as AbTYRIs with IC_50_ values between 0.0018 and 0.089 µM on diphenolase activity [47]. The best activity was observed for derivative **29a** with an IC_50_ value of 0.0018 µM in comparison to the standard kojic acid (**5**, IC_50_ = 16.83 µM). The substitution at the benzyl ring seems to be important for obtaining a higher activity (Figure 17). 

### 2.7. Tetrazoles

**Figure 18:**
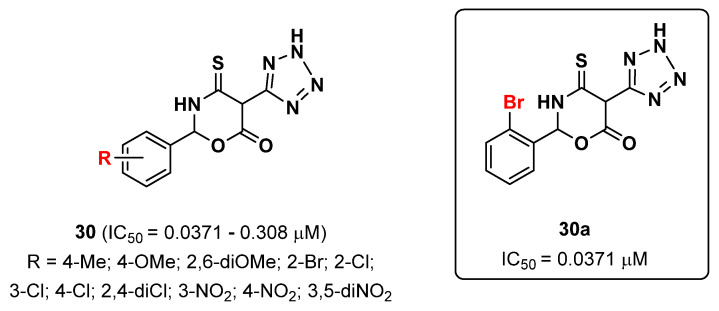
Tetrazole-containing TYRIs **30**.

In 2018, Qamar and co-workers reported a new class of TYRIs bearing the 1,3-oxazine tetrazole scaffold (**30**, Figure 18) [48]. All of the synthesized compounds proved to inhibit l-dopa (**2**) oxidation with IC_50_ values in the range of 0.0371–0.308 μM, displaying a more significant activity if compared to kojic acid (**5**, IC_50_ = 16.832 μM). The most potent compound **30a** has a 2-bromophenyl portion and inhibited TYR activity in a competitive inhibition manner. The binding mode of **30a** was probed with molecular docking which indicated that this compound forms an H-bond with His244 through the oxygen atom, while the bromophenyl moiety establishes hydrophobic contacts with His85. 

### 2.8. Furan Derivatives

**Figure 19:**
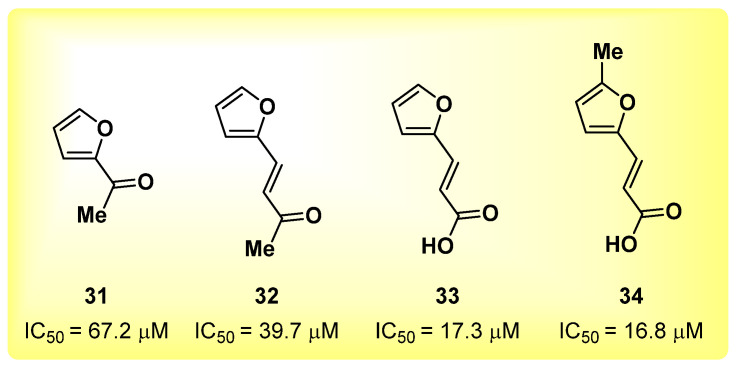
Inhibitory activities of keto and carboxyl furan derivatives **31**–**34** against AbTYR.

In 2019, Barros et al. described the activity of keto and carboxyl furan derivatives (**31**–**34**, Figure 19) against AbTYR [49]. All the compounds were able to inhibit the enzyme with IC_50_ values ranging from 16.8 to 67.2 μM, employing l-dopa (**2**) as a substrate. The most active compounds, **33** and **34**, showed a competitive inhibition mechanism and, according to docking simulations, their highest activity might be attributed to the longer chain linked to the furan ring, which allows the carboxyl group to penetrate deeper into the active site, enabling the interaction with the copper ions. Moreover, the methyl group of **34** established additional hydrophobic interactions that might explain the slightly higher activity over its analogue **33**.

**Figure 20:**
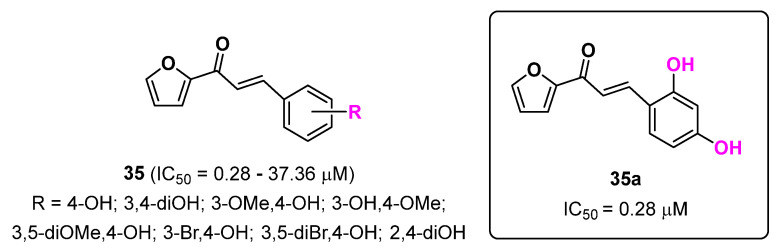
TYRIs combining chalcone and furan moieties.

Jung and co-workers designed a new series of TYRIs by combining chalcone scaffolds with furan moieties [50]. The compounds designed in this fashion (**35** Figure 20) inhibited the diphenolase activity with IC_50_ values in the range 0.28–37.36 μM, except for the 4-hydroxylphenzl, 3-metoxy-4-hydroxyphenyl, 3,5-dimetoxy-4-hydroxyphenyl, and 3-bromo-4-hydroxyphenyl substituted derivatives which showed no activity against AbTYR. The most potent compound **35a** displayed a remarkably higher affinity towards the enzyme than the positive control, kojic acid (**5**, IC_50_ = 33.47 μM). According to kinetic studies, **35a** is a mixed-type inhibitor and it is also able to decrease the enzyme activity in α-MSH and IBMX-stimulated B16F10 melanoma cells, reducing melanin synthesis. Moreover, a reduction of TYR expression in B16F10 cells in the presence of inhibitor **35a** was detected as well.

**Figure 21:**
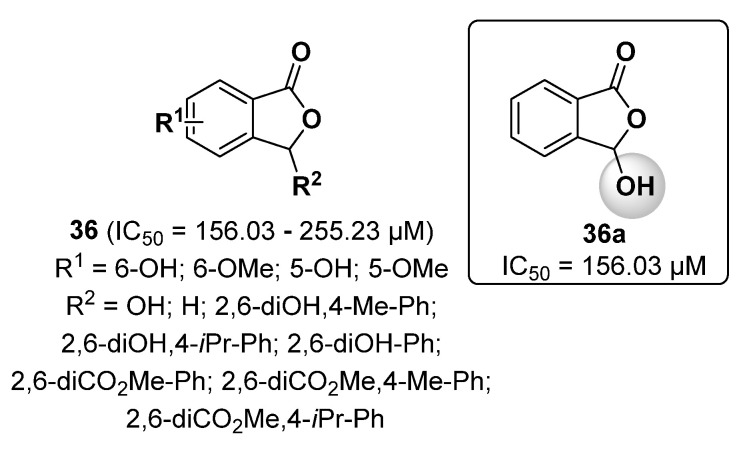
Derivatives of isobenzofuran-1(3*H*)-one (**36a**) and their inhibitory profile.

In 2021, Figueroa-Villar et al. prepared a new class of isobenzofuran-1(3*H*)-ones (**36**, Figure 21) as TYRIs [51]. The authors employed l-tyrosine (**1**) as a substrate to evaluate the percentage of inhibitory activity. Three compounds showed mediocre activity with IC_50_ values between 156.03 and 255.23 µM (Figure 21). The interactions with the enzyme were evaluated via ligand–enzyme NMR studies and docking investigations showing similar binding modes as the positive control, kojic acid (**5**, IC_50_ = 5.71 µM), however this is not reflected in the inhibitory activity. 

**Figure 22:**
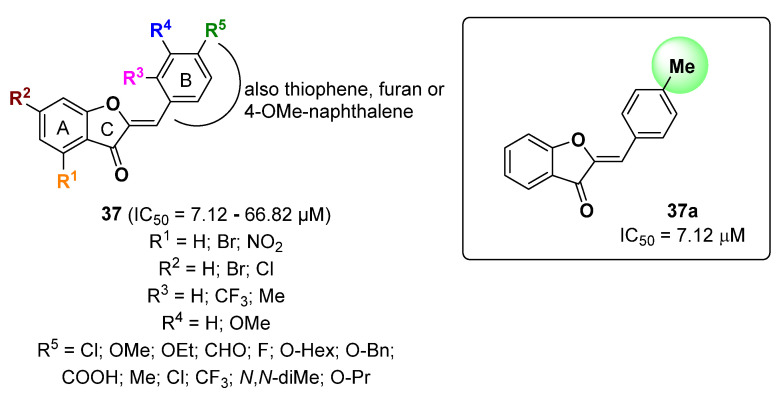
Chemical structures of aurone derivatives **37** and their activities as AbTYRIs.

In 2022, Sadiq and co-workers identified a new series of (*Z*)-2-benzylidenebenzofuran-3(2*H*)-ones (**37**, Figure 22), also called aurones, as AbTYRIs [52]. Their activity was evaluated by using l-dopa (**2**) as a substrate and their inhibition values between 7.12 and 66.82 µM were determined by comparison to kojic acid (**5**, IC_50_ = 16.69 µM) as a positive control. The best result was obtained for derivative **37a** with an IC_50_ value of 7.12 µM which appears to be a non-competitive inhibitor. SAR analysis revealed that, within the series, the benzofuranone moiety is fundamental for TYR inhibition as well as the phenyl ring B. The presence of hydrophilic electron-withdrawing groups (EWGs) on the phenyl ring B decreases the activity, whereas the hydrophobic ones increase it. Considering EDGs, the hydrophilic ones enhance the activity; on the contrary, the hydrophobic ones reduce it. Furthermore, the carbonyl group on the C-ring establishes H-bond interactions with various amino acids and the substitution with hydrophobic groups on the A-ring is favorable for the activity (Figure 22).

### 2.9. Thiophene Derivatives

**Figure 23:**
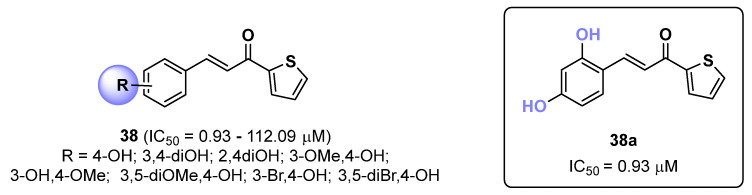
Thiophene derivatives.

In 2018, Kim and co-workers described the anti-tyrosinase activity of eight 3-(substituted phenyl)-1-(thiophen-2-yl)prop-2-en-1-one analogs (**38**, Figure 23) [46]. Among the eight synthesized compounds, five derivatives were able to inhibit the oxidation of l-dopa (**2**) with IC_50_ values ranging from 0.93 to 112.09 μM. The most active derivative **38a** (IC_50_ = 0.93 μM), bearing the 2,4-dihydroxyphenyl moiety, proved to be more potent than the reference compound, kojic acid (**5**, IC_50_ = 0.93 μM), and inhibited TYR activity with a competitive inhibition mode. According to molecular docking studies, **38a** might bind to the catalytic site of TYR by forming two H-bonds with Asn260 and Met280 through its hydroxyl groups and hydrophobic interactions with Met257, Val248, Phe264, Val283, and Ala286 through the thiophene and phenyl rings. Furthermore, compound **38a** reduced melanin content in α-MSH and IBMX-stimulated B16F10 melanoma cells in a dose-dependent manner by decreasing the intracellular TYR activity level. 

### 2.10. Isoxazoles 

**Figure 24:**
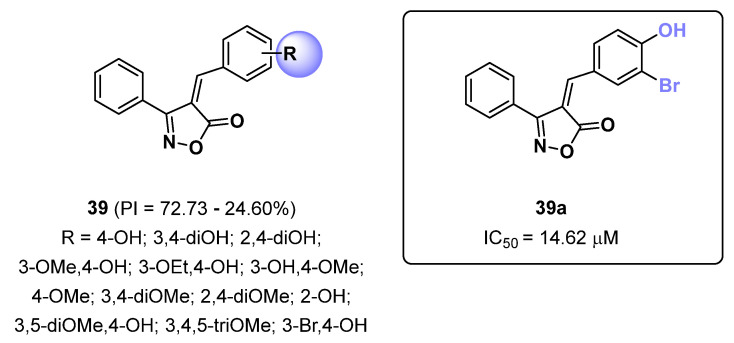
(*Z*)-4-(Substituted benzylidene)-3-phenylisoxazol-5(4*H*)-one derivatives.

Kim et al. reported the TYR inhibitory activity of (*Z*)-4-(substituted benzylidene)-3-phenylisoxazol-5(4*H*)-ones (**39**, Figure 24) with inhibition percentages in the range 72.73–24.60% [53]. The most active inhibitor **39a** showed an IC_50_ value of 14.62 μM being more than twofold more potent than kojic acid (**5**, IC_50_ = 37.86 μM). This compound is a competitive inhibitor of TYR and, according to docking simulation, it might bind the enzyme active site by forming an H-bond with Met280 through its hydroxyl group and hydrophobic contacts with the residues Ala286, Val283, and Phe264. Furthermore, **39a** was able to reduce cellular TYR activity in B16F10 melanoma cells as well as melanin synthesis. 

**Scheme 2:**
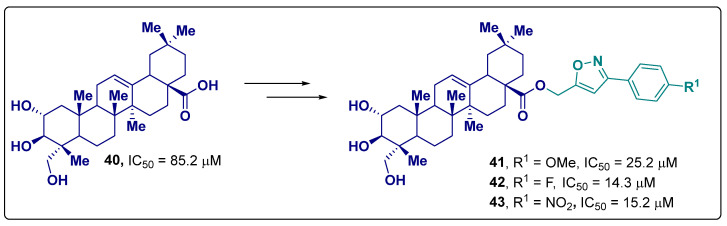
Derivatives of arjunolic acid (**41**–**43**).

Shaik and co-workers described a series of new isoxazole analogs (**41**–**43**, Scheme 2) of arjunolic acid (**40**, Scheme 2) as TYRIs [54]. After extracting the arjunolic acid (**40**) from the stems of *X. granatum* [55], the authors synthesized derivatives **41**–**43** via a two-step procedure. The biological assays revealed an improved inhibitory activity of derivatives **41**–**43** in comparison to the lead compound arjunolic acid (**40**, IC_50_ = 85.2 μM) and the reference inhibitor kojic acid (**5**, IC_50_ = 41.5 μM). The best activity was observed for **42**, possessing a fluorine atom at the *para* position of the phenyl ring with an IC_50_ value of 14.3 μM. 

### 2.11. Oxazolines

**Figure 25:**
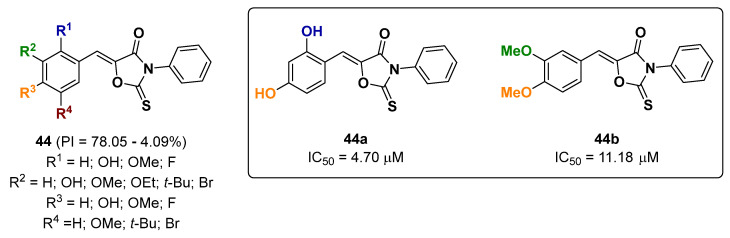
2-Thioxooxazoline-4-one derivatives **44** as tyrosinase inhibitors.

Moon and co-workers reported a series of 2-thioxooxazoline-4-one derivatives (**44**, Figure 25) possessing the β-phenyl-α,β-unsaturated carbonyl scaffold, which is well-known for TYR inhibition [56]. The newly prepared molecules showed an inhibitory activity between 78.05 and 4.09% in comparison to kojic acid (**5**, PI = 58.09%; IC_50_ = 23.18 µM). The two most active compounds, **44a** and **44b**, proved to be competitive inhibitors with IC_50_ values of 4.70 and 11.18 µM, respectively, and both were not cytotoxic towards B16F10 melanoma cells.

### 2.12. Oxadiazoles

**Figure 26:**
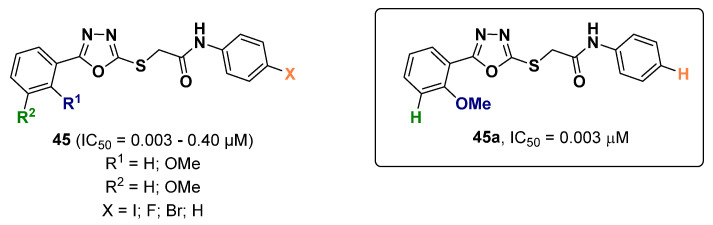
The tyrosinase inhibition potential of 1,3,4-oxadiazoles derivatives **45**.

Lee et al. described a new class of 1,3,4-oxadiazoles (**45**, Figure 26) as TYRIs [57]. All synthesized compounds showed an excellent inhibition of diphenolase activity with IC_50_ values between 0.003–0.40 µM, more pronounced than the reference, which was kojic acid (**5**, IC_50_ = 16.83 µM). Computational studies suggested that the substitution at the acetamide portion is favorable for the activity; In particular, the increment of the size of the halogen atom at the *para*-position increases the interactions with the enzyme improving the inhibition. The presence of the *ortho*-substituted methoxy phenyl group at the 5-position of the 1,3,4-oxadiazole heterocyclic ring creates a good interaction pattern with the enzyme conferring a good inhibition rate (Figure 26). In contradiction, the inhibitor **45a**, which showed the best activity does not possess any substituent at the *para* position of the acetamide portion. Kinetic studies revealed a non-competitive mode of action, and it drastically diminished the α-MSH stimulated melanin synthesis on B16F10 cells.

**Figure 27:**
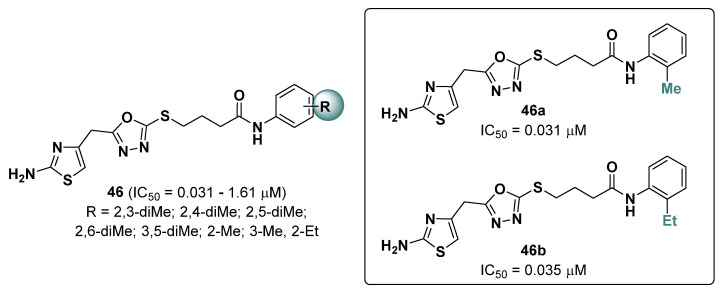
2-Aminothiazole-oxadiazole bi-heterocyclic hybrids **46** and their potential as AbTYR inhibitors.

A new class of 2-aminothiazole-oxadiazole bi-heterocyclic hybrids characterized by a butanamide moiety was reported by Kim and co-workers as TYRIs [58]. The synthesized compounds (**46**, Figure 27) showed IC_50_ values in the range of 0.031–1.61 μM: lower in comparison to the standard, kojic acid (**5**, IC_50_ = 16.83 µM). The two molecules **46a** and **46b** possessing 2-methylphenyl and 2-ethylphenyl moieties, respectively, in the phenyl ring proved to be the most active with IC_50_ values of 0.031 and 0.035 μM, respectively (Figure 27). Docking studies revealed good interactions with the main amino acids of the catalytic pocket.

### 2.13. Thiazoles

**Figure 28:**
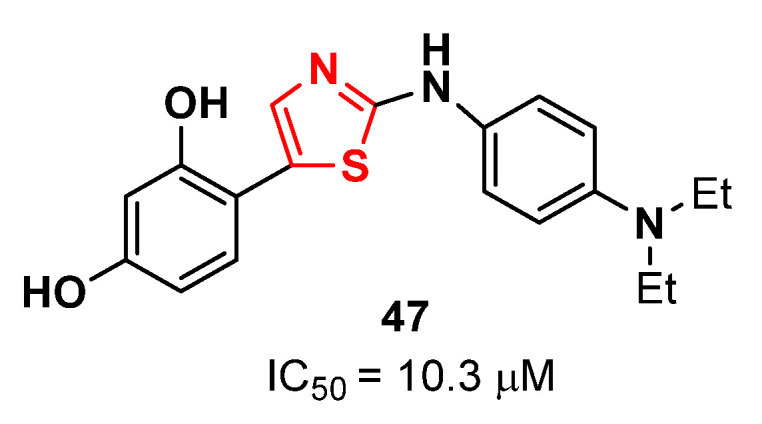
Thiazole derivative **47** which was discovered as TYRI from virtual screening.

In 2018, Li et al. applied a shape-based virtual screening approach to discover new TYRIs [59]. As a result, compound **47**, Figure 28, was identified as an effective TYRI on the diphenolase activity with an IC_50_ value of 10.3 μM. Derivative **47** acted as a competitive inhibitor and, as suggested by docking analysis, it might occupy the enzyme active site with the resorcinol moiety oriented towards the copper ions and stabilized by aromatic and hydrophobic interactions with His263, Ser282, and Val283. The thiazole moiety could form pi-stacking contacts with Phe264 while the *p*-phenylenediamine moiety is involved in hydrophobic interactions with Phe264 and Val248.

**Figure 29:**
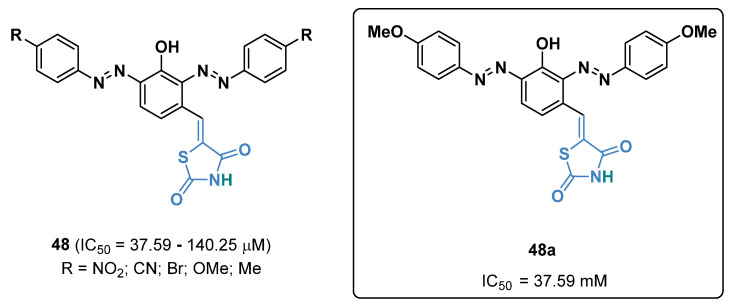
Azo-hydrazone tautomeric dyes bearing a thiazolididinone moiety derivatives.

In the same year, Rezaei and co-workers reported the TYR inhibitory activity of a new class of azo-hydrazone tautomeric dyes bearing a thiazolididinone moiety (**48**, Figure 29) [60]. These derivatives (see Figure 29) were able to inhibit the diphenolase activity of AbTYR with IC_50_ values ranging between 37.59–140.25 μM, thus showing a lower potency if compared to the reference, kojic acid (**5**, IC_50_ = 29.44 μM). In more detail, derivative **48a** proved to be the most active among the synthesized compounds, with an IC_50_ value of 37.59 μM. According to kinetic studies, it is a competitive inhibitor. Docking studies suggested that **48a** might elicit several hydrophobic interactions and H-bonds with the residues of the catalytic pocket, which might be crucial for the binding to the active site of TYR. 

**Figure 30:**
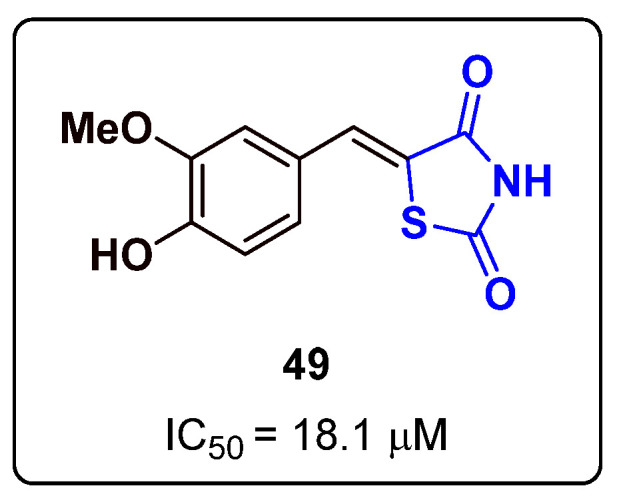
Chemical structure of 5HTM (**49**).

Bang et al. identified the synthetic derivative (*Z*)-5-(3-hydroxy-4-methoxybenzylidene)-2-thioxothiazolidin-4-one (5HTM, **49**, Figure 30) as AbTYR inhibitor in a cell-free based assay employing l-tyrosine (**1**) as a substrate [61]. 5HTM resulted to be more potent than the kojic acid (**5**, IC_50_ = 35.1 μM) displaying an IC_50_ value of 18.1 μM and it acts as competitive inhibitor of AbTYR (Figure 30). Furthermore, the anti-melanogenic effect of 5HTM was evaluated in vivo by using HRM2 hairless mice. After the irradiation with UVB, 5HTM was topically administered on the mouse dorsal skin once per day for two weeks. The outcomes highlighted a reduction of UVB-induced melanogenesis and a skin-lightening effect that was higher than that observed for kojic acid (**5**). Western blot analysis confirmed that the in vivo anti-melanogenic effect was related to the inhibition of the TYR enzyme. 

**Figure 31:**
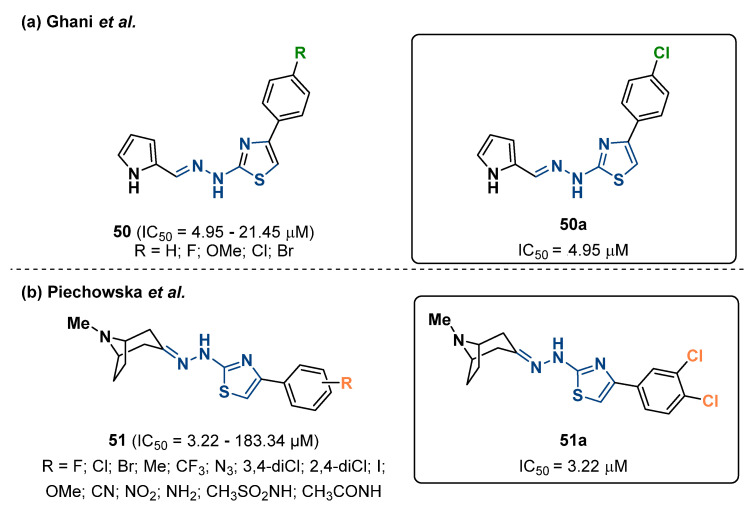
TYRIs containing hydrazinyl thiazole moieties. (**a**) Ghani et al. [22]; (**b**) Piechowska et al. [62,63].

Hydrazinyl thiazoles were exploited over the years as scaffolds for the design of new TYRIs. A successful example is represented by the study of Ghani and co-workers who synthesized a series of hydrazone-bridged thiazole-pyrrole derivatives (**50**, Figure 31a) whose TYR activity was evaluated by using l-dopa (**2**) as a substrate [22]. All of the compounds were able to inhibit TYR activity with IC_50_ values between 4.95 and 21.45 μM. The most potent derivative **50a** showed an affinity towards TYR comparable to that of the positive standard, kojic acid (**5**, IC_50_ = 4.43 μM). Kinetic studies were performed on the tested molecules revealing a competitive inhibition manner. 

Piechowska et al. designed hybrid structures by combining hydrazinyl thiazole scaffolds with a tropinone ring, an alkaloid found in plants from the *Solanaceae* species [62,63]. The resulting compounds (**51**, Figure 31b) proved to inhibit the diphenolase activity of TYR with IC_50_ values within the range 3.22–183.34 µM. In particular, the most active compound (**51a**) carried the 2,4-dichloro substitution pattern on the phenyl ring, which led to an improvement of the inhibitory activity if compared to the kojic (**5**) and ascorbic (**7**) acids used as references, whose IC_50_ values were 72.27 μM and 386.5 μM, respectively. Concerning the inhibition mechanism, compound **51a** is a non-competitive inhibitor of AbTYR.

**Figure 32:**
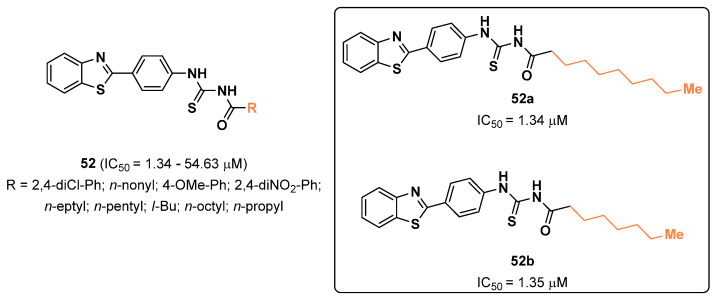
2-Benzothiazole-linked thioureas with inhibitory activity towards TYR.

In 2020 El-Seedi et al. designed a new series of thiourea–heterocyclic hybrids (**52**, Figure 32) by integrating the acyl thiourea with a 2-phenyl benzothiazole moiety [64]. The inhibition of TYR activity was evaluated for all the designed compounds by using l-dopa (**2**) as a substrate. The results indicated that all of the derivatives (**52**) were able to inhibit the enzyme with IC_50_ values between 1.3431–54.6311 μM. In more detail, most of the compounds showed an improved activity in comparison to kojic acid (**5**, IC_50_ = 16.832 μM). It was observed that the molecules carrying a long aliphatic chain exhibited a higher potency as they might be able to form stronger interaction with the enzyme. The best inhibitors (**52a**–**52b**) were subjected to kinetic and docking studies showing a non-competitive mode of action. Docking simulations suggested that **52a** could interact with the TYR binding site by engaging two H-bonds with Glu322 through the thiourea amino groups, while the 2-phenyl benzothiazole system might be involved in pi-stacking interactions with His244 and the copper-coordinating His263. 

**Figure 33:**
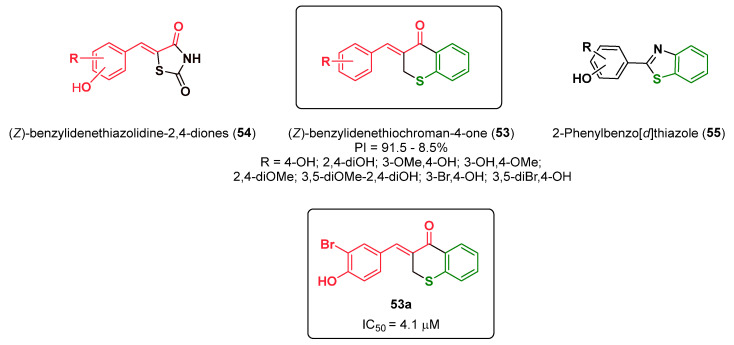
Rational design of TYRIs bearing the benzylidenethiochroman-4-one scaffold.

**Figure 34:**
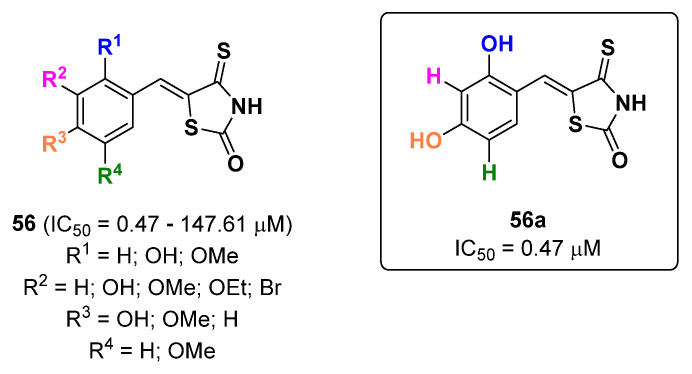
(*Z*)-5-(Substituted benzylidene)-4-thioxothiazolidin-2-one derivatives (**56**) as tyrosinase inhibitors.

Bang and co-workers reported the synthesis of a new series of inhibitors (**53**, Figure 33) designed by combining the scaffolds of two already known classes of TYRIs, the benzylidene thiazolidinedione (**54**, Figure 33) [65] and the phenyl benzothiazole (**55**, Figure 33) [66,67]. The resulting benzylidenethiochroman-4-ones **53** were able to inhibit the monophenolase activity showing inhibition percentages between 91.5 and 8.5%. The IC_50_ value was computed for one of the most active compounds of this series, the (*Z*)-3-(hydroxyl-substituted-benzylidene)thiochroma-4-one (MHY1498, **53a**) using l-dopa (**2**) as a substrate. MHY1498 (**53a**) displayed an IC_50_ value of 4.1 μM, being about fivefold more potent than kojic acid (**5**, IC_50_ = 22.0 μM), which was used as reference. The analysis of the Lineweaver–Burk plot revealed that this compound is a competitive inhibitor. According to docking simulations, hydrophobic interactions seem to play a major role in the binding with the active site of AbTYR. Cell-based assays, performed on α-MSH-stimulated B16F10 melanoma cells, showed that MHY1498 (**53a**) was able to decrease melanin synthesis by reducing TYR activity.

(*Z*)-5-(SubstitutedBenzylidene)-4-thioxothiazolidin-2-one derivatives (**56**, Figure 34) were reported by Moon and co-workers as inhibitors of AbTYR with IC_50_ values between 0.47 and 147.61 μM on monophenolase activity; see Figure 34 [68]. Between the synthesized compounds, seven molecules showed better activity in comparison to the reference, kojic acid (**5**, IC_50_ = 66.30). However, the best activity was obtained for **56a** with an IC_50_ value of 0.47 μM which is a competitive inhibitor. Docking studies carried out by using a hTYR homology model revealed a possible inhibition of hTYR. Taken together, **56a** proved to have better anti-melanogenic effects on B16F10 melanoma cells than kojic acid (**5**).

**Figure 35:**
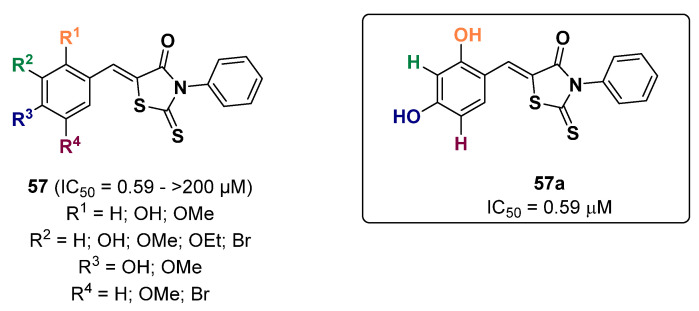
(*Z*)-5-(Substituted benzylidene)-3-phenyl-2-thioxothiazolidin-4-one derivatives **57**, that showed promising inhibitory characteristics.

Later on, the same group described a series of (*Z*)-5-(substituted benzylidene)-3-phenyl-2-thioxothiazolidin-4-one compounds (**57**, Figure 35) as anti-melanogenic molecules [69]. Their inhibitory activity against TYR was evaluated using l-tyrosine (**1**) as a substrate and the IC_50_ values observed are between 0.59 and >200 µM, compared to kojic acid (**5**, IC_50_ = 17.05 µM). The most promising result was obtained for derivative **57a** with an IC_50_ value of 0.59 µM. It is a competitive inhibitor and docking studies carried out on hTYR suggested that it could also be active against hTYR. In vitro studies conducted using B16F10 cells indicated that compound **57a** inhibits cellular TYR and melanin production more than kojic acid (**5**) without being cytotoxic. 

**Figure 36:**
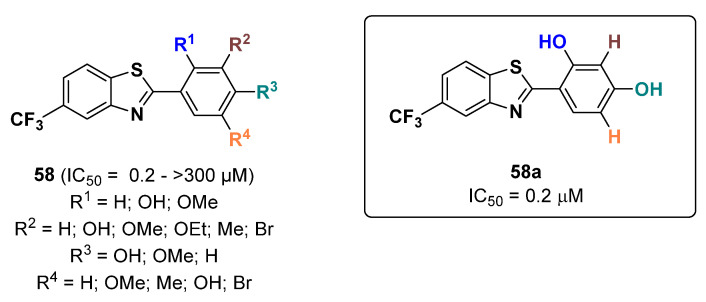
Inhibitory activities towards TYR of 2-(aryl)-5-(trifluoromethyl)benzo[*d*]thiazoles **58**.

**Figure 37:**
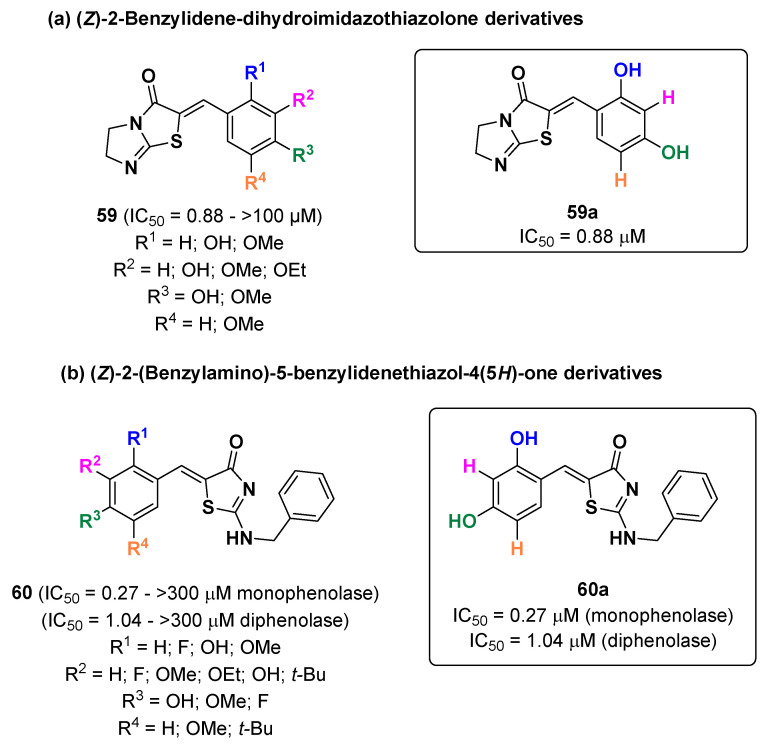
(*Z*)-2-Benzylidene-dihydroimidazothiazolone derivatives (**59**) (**a**). (*Z*)-2-(Benzylamino)-5-benzylidenethiazol-4(5*H*)-one derivatives (**60**) (**b**).

The same group described also a series of 2-(substituted phenyl)-5-(trifluoromethyl)benzo[*d*]thiazoles derivatives (**58**, Figure 36) as TYRIs [70]. IC_50_ values between 0.2 and >300 µM were obtained on monophenolase activity, compared to kojic acid (**5**, IC_50_ = 12.6 µM). The inhibitor that showed the highest inhibitory activity is **58a** with an IC_50_ value of 0.2 µM; **58a** displayed a competitive inhibition mode, and it also inhibited cellular TYR and melanin production on B16F10 cells. In silico studies supported also a potential inhibitory activity on hTYR. 

(*Z*)-2-Benzylidene-dihydroimidazothiazolone derivatives (**59**, Figure 37a), were also reported by Moon et al. as TYRIs with IC_50_ values between 0.88 and >100 µM using l-tyrosine (**1**) as a substrate, compared to kojic acid (**5**, IC_50_ = 84.41 µM) [71]. The best inhibitor (**59a**) was a competitive one with an IC_50_ value of 0.88 µM which showed a higher anti-melanogenic activity in comparison to kojic acid (**5**). In 2023, the same group described a series of (*Z*)-2-(benzylamino)-5-benzylidenethiazol-4(5*H*)-one derivatives (**60**, Figure 37b) as TYRIs [72]. Their inhibitory activity was evaluated both on monophenolase and diphenolase activities, showing IC_50_ values in the range of 0.27 → 300 μM and 1.04 → 300 μM, respectively, compared to kojic acid (**5**, with an IC_50_ of 28.6 μM on monophenolase and an IC_50_ of 0.035 μM on diphenolase). In silico assays revealed a good binding mode of the most active derivative (**60a**, IC_50_ = 0.27 μM and 1.04 μM) with the catalytic site of the enzyme. In both the described series of compounds the presence of a hydroxyl group in *ortho* and *para* position of the phenyl ring led to the best results.

**Figure 38:**
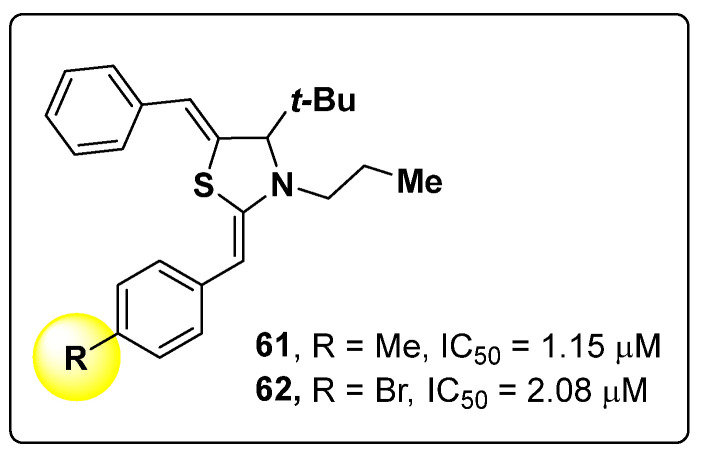
Thiazolidin-2-imine derivatives **61** and **62**, with IC_50_ values in the very low micromolecular range.

Simpson and co-workers described two novel thiazolidin-2-imine derivatives (**61**–**62**, Figure 38) as TYRIs [73]. The two compounds **61** and **62** showed good inhibitory activities of 1.15 and 2.08 μM, respectively. These were higher in comparison to the positive control kojic acid (**5**, IC_50_ = 16.03 μM) on diphenolase activity (Figure 38). Docking studies revealed that both molecules fit well in the catalytic pocket of the enzyme and the high activity can be due to the presence of the phenyl rings that increase the hydrophobic interactions.

### 2.14. Hydroxypyridinones 

**Figure 39:**
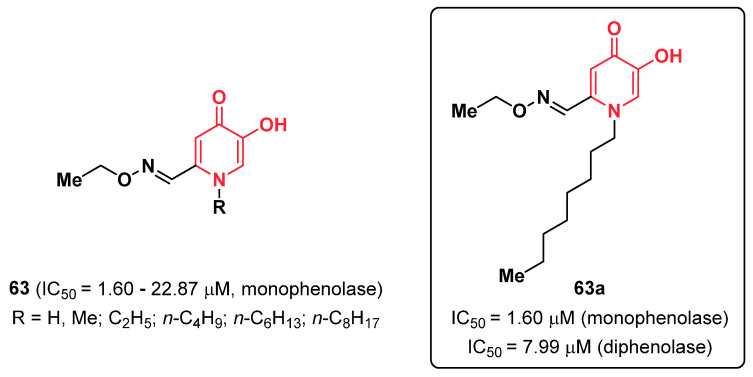
Hydroxypyridinone-based TYRIs (**63**) bearing an oxime moiety.

Hydroxypyridinone is structurally related to kojic acid (**5**), and it also possesses anti-tyrosinase activity [74,75]. In 2018, Zhou’s research group reported a series of hydroxypyridinone derivatives (**63**, Figure 39) containing an oxime ether moiety, as effective TYRIs on monophenolase activity [76]. This class of inhibitors showed IC_50_ values in the range 1.60–22.87 μM. The most potent compound **63a** (IC_50_ = 1.60 μM) (Figure 39) proved to be more than sevenfold more active than kojic acid (**5**, IC_50_ = 12.24 μM). The inhibitory effect of **63a** on the diphenolase activity was evaluated as well. The results pointed out that **63a** inhibited the oxidation of l-dopa (**2**) with an IC_50_ value of 7.99 μM adopting a mixed-type inhibition mode. Docking studies suggested that the 4-oxy and 5-hydroxy moieties of **63a** might be involved in the coordination of the copper ions while the aliphatic chain could be involved in hydrophobic interactions with the surrounding residues. Finally, the anti-browning effect of **63a** on fresh-cut apples was evaluated revealing that this derivative delayed the browning process, and thus represents a promising food preservative. 

**Figure 40:**
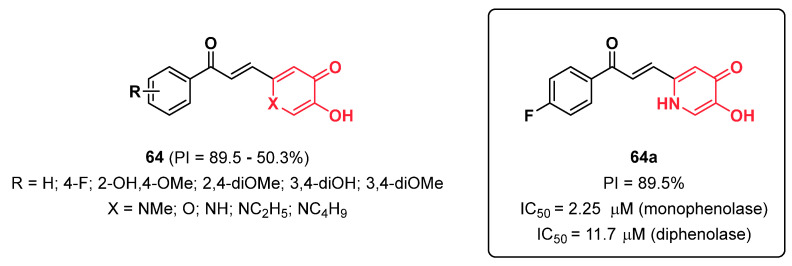
TYRIs bearing a chalcone-hydroxypyridinone scaffold.

In 2020, the same research group applied a hybridization strategy by combining the hydroxypyridinone moiety with the chalcone scaffold, which is also known to have anti-tyrosinase properties [77,78]. All of the synthesized compounds (**64**, Figure 40) were tested on the monophenolase activity showing inhibition percentages between 89.5 and 50.3% while the positive standard, kojic acid (**5**), displayed an inhibition percentage of 75.4%. The IC_50_ values were computed for the most potent compounds, and it was found that the most active derivative (**64a**) displayed IC_50_ values of 2.25 μM and 11.7 μM, on the monophenolase and diphenolase activities, respectively (Figure 40). As seen for the previous hydroxypyridinone derivatives, also in this case docking analysis revealed that **64a** might bind TYR by coordination of the metal ions through the 4-oxy and the 5-hydroxy groups. 

**Figure 41:**
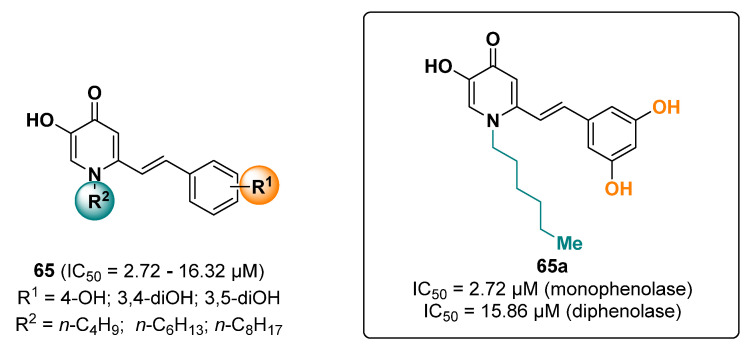
TYR-inhibitory profile of stilbene-hydroxypyridinone hybrids **65**.

In 2022, Zhou’s group described a new series of stilbene–hydroxypyridinone hybrids (**65**, Figure 41) as TYRIs [79]. Their inhibitory activity was evaluated on monophenolase, obtaining IC_50_ values between 2.72 and 16.32 µM in comparison to kojic acid (**5**, IC_50_ = 12.52 µM). For the most active compound **65a**, the diphenolase activity was also tested showing an IC_50_ value of 15.86 µM and it is a competitive-noncompetitive mixed-type inhibitor (Figure 41). Docking studies revealed that the 4-oxo function of the pyridinone ring of compound **65a** coordinates the copper ion, which also showed an anti-browning effect on freshly cut apples as seen for the inhibitors previously reported by this research group.

### 2.15. Quinoline Derivatives

**Figure 42:**
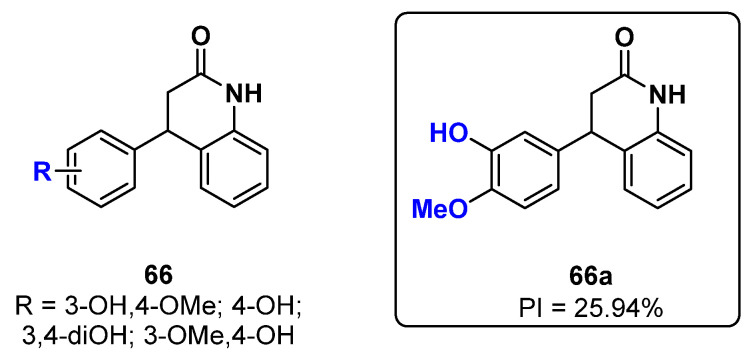
Dihydroquinolinones and their TYR inhibitory behavior.

In 2019, Ullah and co-workers disclosed the anti-tyrosinase activity of a small set of dihydroquinolinone derivatives (**66**, Figure 42) obtained by intramolecular cyclization of the phenyl cinnamide [80]. Among the synthesized molecules, only the compound carrying the 3-hydroxy-4-methoxyphenyl substitution pattern (**66a**) displayed TYR inhibition on the monophenolase activity, with a percentage of 25.94 showing a potency comparable to the potency retrieved for kojic acid (**5**, PI = 21.39%), which was used as a reference in this study. 

**Figure 43:**
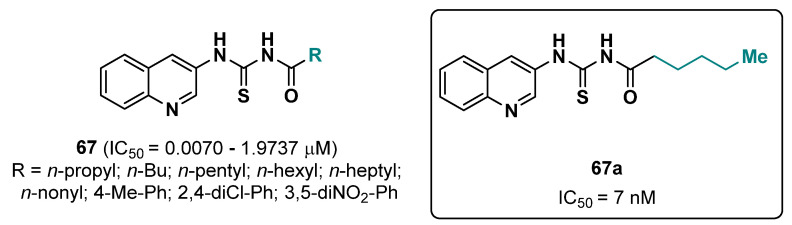
Quinolinyl based acyl thiourea derivatives (**67**), with representative **67a** reaching a single digit nanomolar IC_50_ value.

Acyl thiourea represents a privileged scaffold in medicinal chemistry due to its ability to coordinate metal ions [81,82]. Some acyl thiourea-containing compounds were identified as TYRIs [83]. In this context, in 2019, Mustafa et al. reported a novel class of TYRIs combining the quinoline and acyl thiourea moieties in the same molecular framework [84]. All of the resulting molecules (**67**, Figure 43) proved to significantly inhibit the diphenolase activity showing IC_50_ values in the range of 0.0070 to 1.9737 μM. Among them, the pentyl substituted derivative **67a** exhibited the highest inhibition (IC_50_ = 70 nM) in regard to the other tested compounds and the positive standard kojic acid (**5**, IC_50_ = 16.832 μM). SAR analysis pointed out that the length of the alkyl chain greatly affects the anti-tyrosinase activity as too-short chains poorly interact with the enzyme leading to affinity loss, while too-long chains might exert the same negative effect because of the steric hindrance. Compound **67a** carried a moderate alkyl chain whose length is optimal for the interaction with the enzyme. Similarly, also the presence of a phenyl ring positively influences the TYR inhibitory activity, while substituted phenyl moieties reduce the affinity due to the increment of the steric hindrance. Compound **67a** acted as non-competitive TYRI and, basing on docking studies, it might bind to the enzyme by forming a H-bond with His244 through its sulfur atom, and hydrophobic contacts with Val283 and His263 by means of the aliphatic chain.

### 2.16. Pyrimidine-Based Derivatives

**Figure 44:**
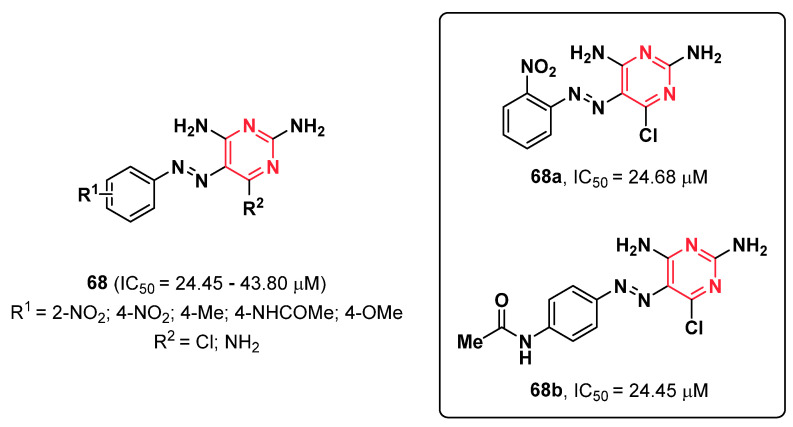
Azo-pyrimidine derivatives that were tested on their inhibitory activity on TYR.

Mirmortazavi et al. designed a novel class of azo-pyrimidine containing a substituted aromatic moiety: see Figure 44 [85]. The synthesized derivatives (**68**, Figure 44) were tested on their diphenolase activity, whereas IC_50_ values in the range of 24.45–43.80 μM were observed. Two effective TYRIs, **68a** and **68b**, were identified which showed inhibitory potencies comparable to that of kojic acid (**5**, IC_50_ = 25.24 μM). Specifically, **68a** and **68b** inhibited TYR with IC_50_ values of 24.68 μM and 24.45 μM, respectively, displaying a non-competitive inhibition mechanism.

**Figure 45:**
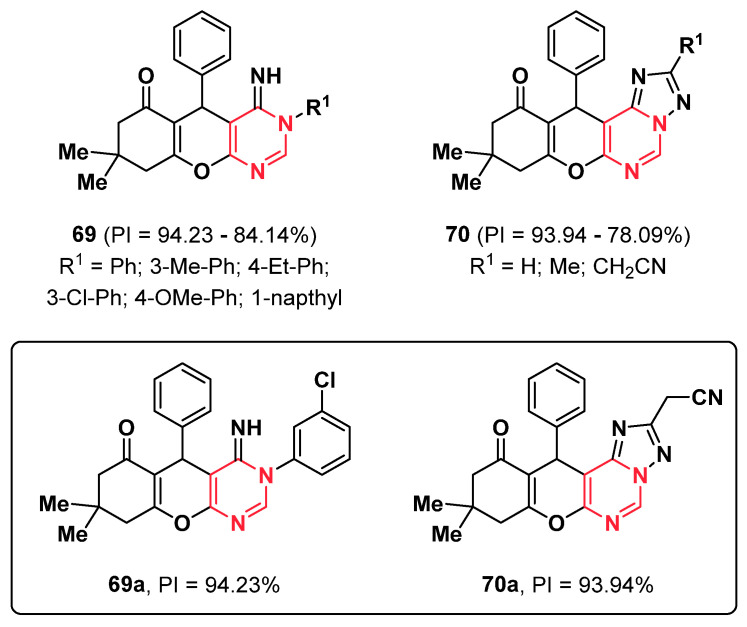
TYRIs bearing the pyranopyrimidine (**69**) and the pyranotriazolopyrimidine (**70**) scaffolds.

Debabbi and co-workers described the syntheses and anti-tyrosinase potencies of two series of compounds featuring different heterocyclic moieties in their structures, which are pyranopyrimidines (**69**, Figure 45) and pyranotriazolopyrimidines (**70**, Figure 45) [86]. The obtained compounds were tested against TYR using l-tyrosine (**1**) as a substrate. Some of the compounds did not show any inhibitory activity but the percentage of inhibition of the active ones ranged between 94.23–84.14% and 93.94–78.09% for **69** and **70**, respectively. **69a** and **70a** proved to be the most effective among the pyranopyrimidine and the pyranotriazolopyrimidine derivatives, respectively (Figure 45). In particular, **69a** inhibited the enzyme activity with an inhibition percentage of 94.23%, while **70a** showed a PI equal to 93.94%. Both compounds proved to be more potent than kojic acid (**5**, PI = 85.50%). SAR analysis revealed that the substituent linked to the phenyl moiety or to the triazole ring strongly influenced the inhibitory activity. The trends observed in the SAR showed that in this position EWGs had positive effects on TYR inhibition.

**Figure 46:**
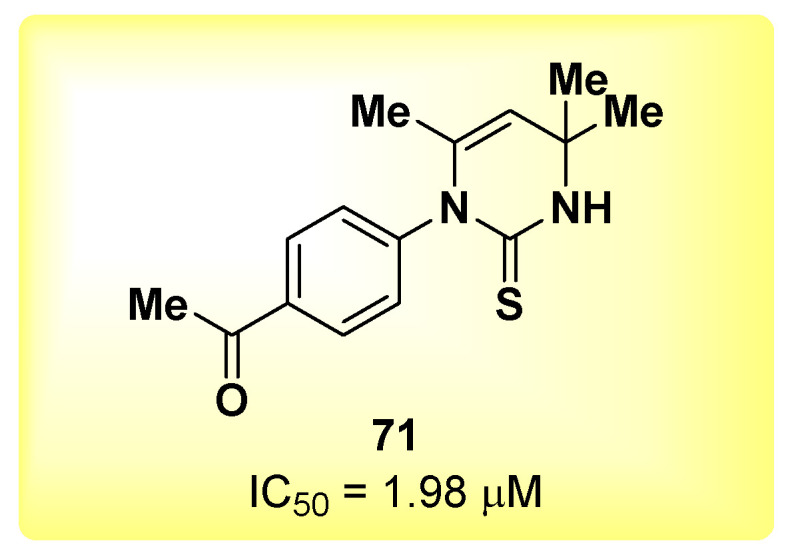
Chemical structure and inhibitory activity of 3,4-dihydropyrimidine-2(1*H*)-thione (**71**).

Acetophenone-based 3,4-dihydropyrimidine-2(1*H*)-thione (**71**, Figure 46) was synthesized by Hökelek et al. Its inhibitory activity against TYR was evaluated on l-dopa oxidation and showed an IC_50_ value of 1.98 µM, compared to kojic acid (**5**, IC_50_ = 15.79 µM) [87]. The binding mode of derivative **71** within the catalytic pocket of TYR was studied via molecular docking, which showed good hydrophobic as well as good hydrophilic interactions.

**Figure 47:**
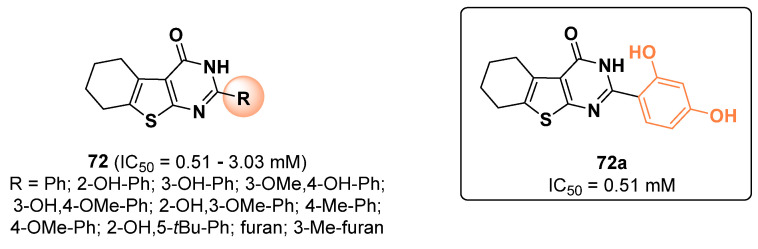
Tetrahydrobenzo[4,5]thieno[2,3-d]pyrimidine-4(3*H*)-one derivatives **72**, which are substituted in the 2-position.

Pozdnyakov and co-workers reported a series of 2-substituted tetrahydrobenzo[4,5]thieno[2,3-d]pyrimidine-4(3*H*)-one derivatives (**72**, Figure 47) as TYRIs [88]. Their inhibitory activity was evaluated, showing IC_50_ values between 0.51 and 3.03 mM in comparison to the positive control kojic acid (**5**, IC_50_ = 0.32 mM). SAR analysis revealed that the presence of hydroxy groups increased the inhibitory activity. This was supported by molecular docking which showed that the metal–ligand interactions with the two copper ions in the catalytic site of the enzyme govern the binding mode.

### 2.17. Oxoquinazoline Derivatives

**Figure 48:**
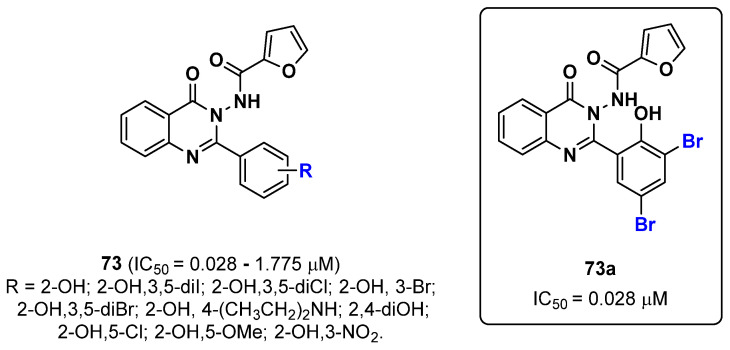
Oxoquinazoline derivatives **73** and their potential in inhibition of TYR.

In 2019, Dige and co-workers reported a new class of 4-oxoquinazolin-3(4*H*)-yl)furan-2-carboxamides (**73**, Figure 48) as effective TYRIs on diphenolase activity, showing IC_50_ values from 0.028–1.775 μM, thus proving to be more potent than kojic acid (**5**, IC_50_ = 16.832 μM) [89]. The data obtained from the in vitro screening highlighted that the presence of EWGs on the phenyl ring is favorable for the anti-tyrosinase activity, while the introduction of EDGs led to loss of affinity. The most potent compound of this series was derivative **73a** which carried two bromine atoms and one hydroxyl group on the phenyl ring. Inhibitor **73a** blocked TYR activity with an IC_50_ value of 0.028 μM and a non-competitive inhibition mechanism. 

### 2.18. Piperazines

**Figure 49:**
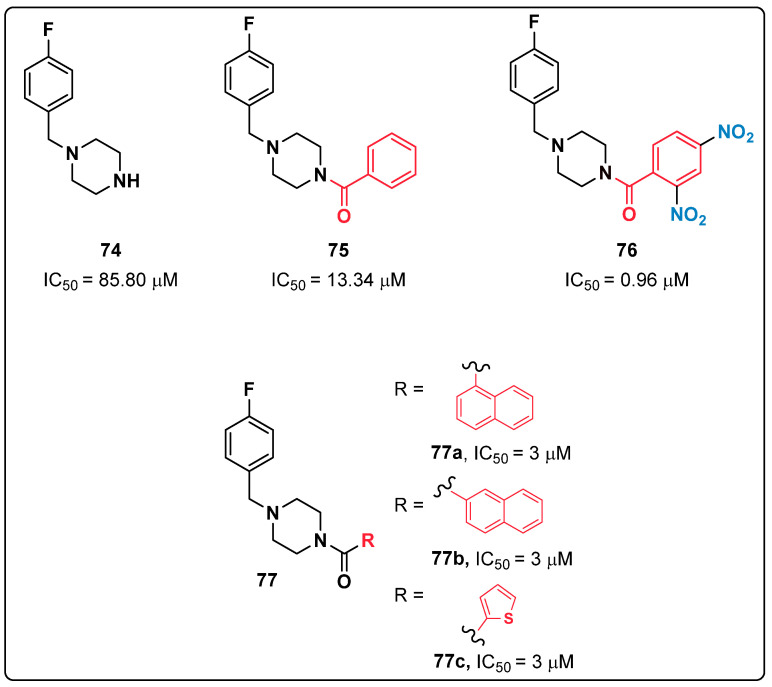
TYRIs bearing the 4-fluorobenzylpiperazine fragment.

De Luca and co-workers reported the anti-tyrosinase activity of some 4-fluorobenzylpiperazine derivatives. Specifically, the authors identified the 4-fluorobenzylpiperazine fragment (**74**, Figure 49) to be the crucial portion for the inhibitory effect on the AbTYR diphenolase activity with an IC_50_ value of 85.80 μM (Figure 49). The substitution of the piperazine ring with a benzoyl moiety (**75**, Figure 49) led to an improvement of the potency allowing to reach an IC_50_ value of 13.34 μM which is comparable to that of kojic acid (**5**, IC_50_ = 17.76 μM) used as a reference [90]. The decoration of the benzoyl moiety with different EWGs and EDGs increased the inhibitory activity towards TYR with the most potent compound **76** (Figure 49), which showed an IC_50_ value of 0.96 μM. Overall, it was observed that the substitutions in the positions 2 and 2,4 of the aroyl moiety are the most favorable for the anti-tyrosinase activity. Derivative **76** revealed to be a competitive inhibitor and was also able to reduce melanin content in α-MSH-stimulated B16F10 cells [91]. Crystallographic studies performed on *Bacillus megaterium* tyrosinase (BmTYR) revealed that **76** binds to the TYR active site with the fluorobenzyl moiety oriented towards the copper ions, while the rest of the molecules engage hydrophobic and H-bond interactions [90]. The replacement of the benzoyl moiety with other heteroaromatic rings (**77**, Figure 49) increased the inhibitory potency if compared to the unsubstituted benzoyl derivative (**75**), despite a reduction of the activity which was observed for this analog, with respect to compound **76** [92]. The best inhibitors of this series were the 1-naphtyl substituted **77a**, the 2-naphtyl analog **77b**, and the 2-thyenil derivative **77c**, which displayed IC_50_ values of 3 μM (Figure 49). 

**Figure 50:**
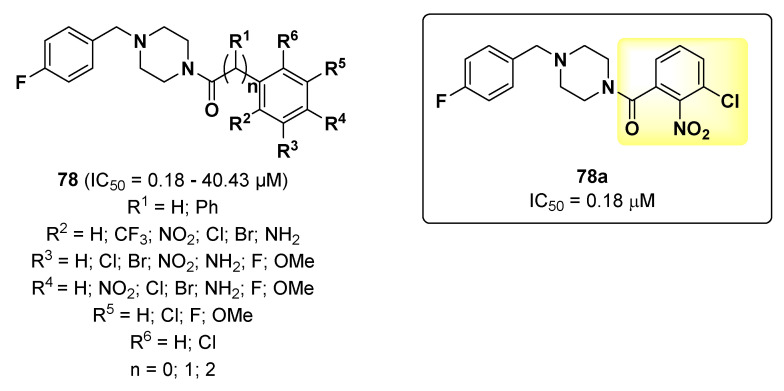
Further 4-fluorobenzylpiperazine derivatives (**78**) which were identified as TYRIs.

As a continuation of the previous studies, De Luca et al. developed further TYRIs bearing the 4-fluorobenzyl piperazine portion as the pharmacophoric feature [93]. Several new compounds (**78**, Figure 50) were synthesized possessing substituents of different nature and their inhibition on diphenolase activity was evaluated. The obtained IC_50_ values (between 0.18 and 40.43 µM) were compared to the reference compound, kojic acid (**5**, IC_50_ = 17.76 μM). SAR studies suggested that the presence of a further phenyl ring (R^1^ substituent) is lowering the inhibitory activity, which may be due to a molecular restriction and/or steric hindrance that reduced the ability to bind the catalytic pocket. Moreover, the introduction of a more flexible linker between the phenyl moiety and the carbonyl group reduced the affinity if compared to respective aroyl analogs. In general, the di-substitution on the phenyl ring led to a better activity especially when -NO_2_ or -CF_3_ groups are in the *ortho* position (Figure 50). The most active compound (**78a**, IC_50_ = 0.18 µM) showed a competitive mode of action performing an anti-melanogenic effect on B16F10 cells without showing cytotoxicity.

**Figure 51:**
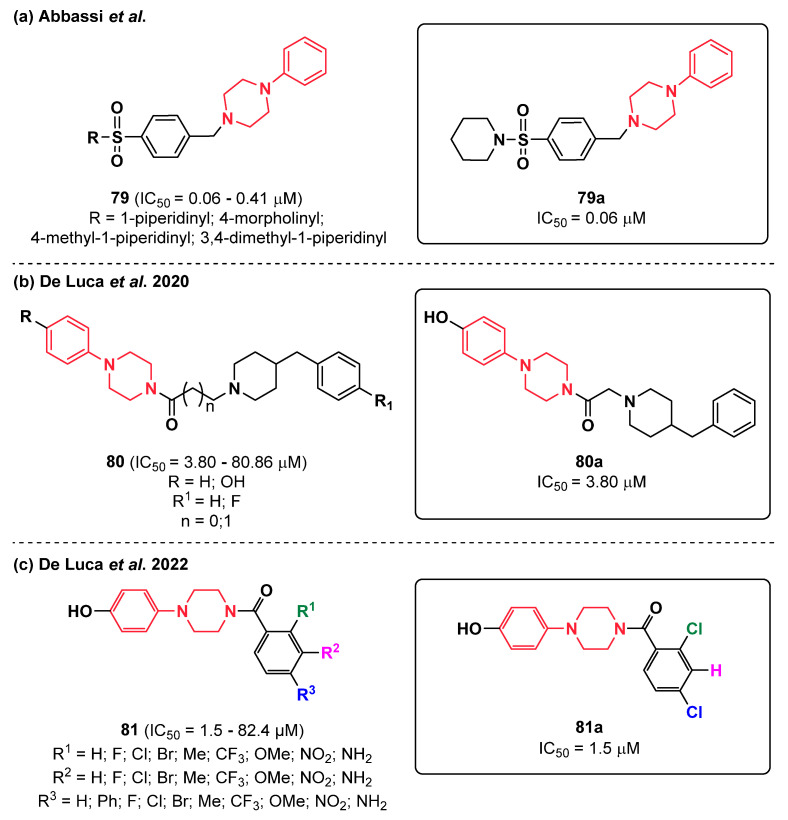
Phenylpiperazine TYRIs (**79**–**81**) from various studies. (**a**) Abbassi et al. [94]; (**b**) De Luca et al. [95]; (**c**) De Luca et al. [96].

In 2020 Abbasi et al. described the in vitro inhibitory effects of bi-heterocyclic sulfonamides containing a phenylpiperazine moiety (**79**, Figure 51a) against TYR employing l-dopa (**2**) as a substrate [94]. These derivatives displayed IC_50_ values in the range of 0.06 to 0.41 μM, thus showing a better potency than kojic acid (**5**, IC_50_ = 16.83 μM), which was used as a positive standard. Among the tested compounds, the derivative bearing the unsubstituted piperidinyl ring **79a**, proved to be the most active with an IC_50_ value of 0.06 μM. SAR analysis revealed that the introduction of methyl groups on the piperidinyl moiety decreased the inhibitory activity as it could cause some repulsion due to steric hindrance, which results in a loss of affinity. According to kinetic studies, compound **79a** is a non-competitive inhibitor of AbTYR. 

In the same year, De Luca and co-workers reported a small series of TYRIs bearing the phenylpiperazine and 4-hydroxylphenylpiperazine portions (**80**, Figure 51b), which proved to inhibit the diphenolase activity with IC_50_ values between 3.80 and 80.86 μM [95]. The results revealed that the hydroxy-substituted derivatives showed a better inhibition than the respective unsubstituted analogs. In particular, the most active compound **80a** was about four times more potent than kojic acid (**5**, IC_50_ = 17.76 μM), displaying an IC_50_ value of 3.80 μM and a non-competitive inhibition mechanism. Docking simulations suggested that the higher activity observed for the hydroxy-substituted derivatives might be attributed to the ability of the hydroxyphenyl moiety to bind to the deep part of the active site with the hydroxy group oriented towards the copper ions. Furthermore, a series of (4-(4-hydroxyphenyl)piperazin-1-yl)arylmethanone derivatives (**81**, Figure 51c) was designed, synthesized, and their inhibitory activity against TYR was evaluated by using l-dopa (**2**) as a substrate showing IC_50_ values between 1.5 and 82.4 µM [96]. The best results were obtained for the derivatives possessing hydrophobic *ortho*-substituents on the aroyl moiety, in particular derivative **81a** with an IC_50_ value of 1.5 µM (Figure 51c). It is a competitive inhibitor with antioxidant activity and possesses anti-melanogenic activity on α-MSH-stimulated B16F10 cells.

**Figure 52:**
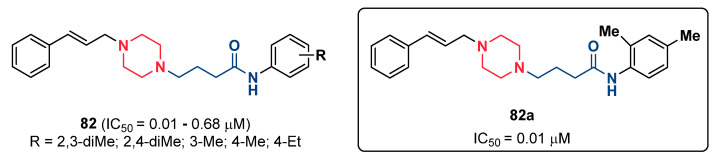
Piperazinyl butanamide-containing TYRIs.

Raza and co-workers designed five *N*-(substituted-phenyl)-4-{(4-[(*E*)-3-phenyl-2-propenyl]-1-piperazinyl} butanamide derivatives (**82**, Figure 52) based on the consideration that several effective TYRIs described in the literature carry an amide or piperazine moiety [97]. Therefore, the authors decided to combine both chemical entities by synthesizing some hybrid molecules, containing both a piperazine and a butanamide portion. All the compounds (**82**) showed a strong inhibition on the diphenolase activity (IC_50_ = 0.01–0.68 μM), superior to that of the positive standard kojic acid (**5**, IC_50_ = 16.84 μM). The most potent compound **82a** (IC_50_ = 0.01 μM) acted as non-competitive inhibitor and showed a depigmenting effect in an in vivo zebrafish assay. Docking analysis revealed that the two aromatic rings of **82a** are mainly involved in pi-stacking interactions with His85, His259, and His263. 

**Figure 53:**
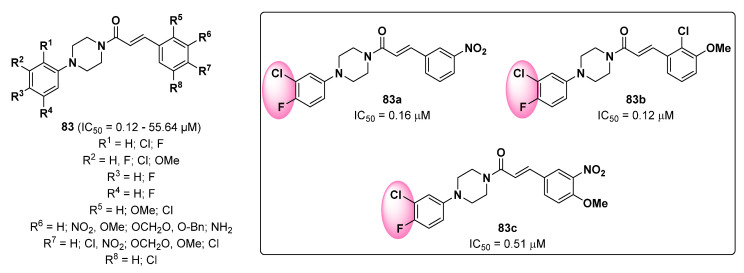
Cinnamic acid derivatives linked to aryl piperazines.

Cinnamic acid derivatives linked to 1-aryl piperazines (**83**, Figure 53) were designed and synthesized by Romagnoli et al. [98]. Their inhibitory activity was evaluated by using l-dopa (**2**) as a substrate with IC_50_ values between 0.12 and 55.64 µM. The presence of a 3-chloro-4-fluorophenyl moiety, as in **83a**, **83b**, and **83c**, at the *N*-1 position of piperazine ring proved to be fundamental for a potent TYR inhibition. Indeed, the two most active inhibitors, **83b** and **83a**, possess this moiety displaying IC_50_ values of 0.12 and 0.16 µM, respectively. On the contrary, the position and number of the substituents on the aryl ring of the cinnamic acid did not affect significantly the anti-tyrosinase activity. The ability to inhibit melanogenesis was also evaluated by using A375 human melanoma cells and an in vivo zebrafish model. The most active compound **83b** significantly reduced the pigmentation of zebrafish at 50 µM but showed 100% mortality in the Fish Embryo Acute Toxicity (FET) test. However, derivative **83c** (IC_50_ value = 0.51 µM) effectively decreases melanogenesis on zebrafish model with no acute toxicity, showing a promising result as an anti-browning candidate.

### 2.19. Quinazolines

**Figure 54:**
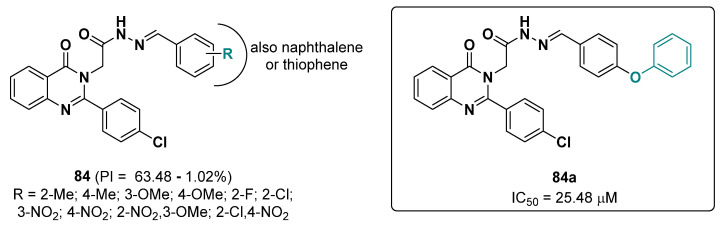
Chlorophenylquinazolin-4(3*H*)-one derivatives **84** with inhibitory potential of TYR.

Iraji and co-workers designed and synthesized a series of chlorophenylquinazolin-4(3*H*)-one derivatives (**84**, Figure 54) containing various aryl acetohydrazides as potential TYRIs [99]. The percentage of TYR inhibition resulted between 63.48 and 1.02%. The best activity was displayed by derivative **84a** with an IC_50_ value of 25.48 µM, compared to kojic acid (**5**, IC_50_ = 9.30 µM).

**Figure 55:**
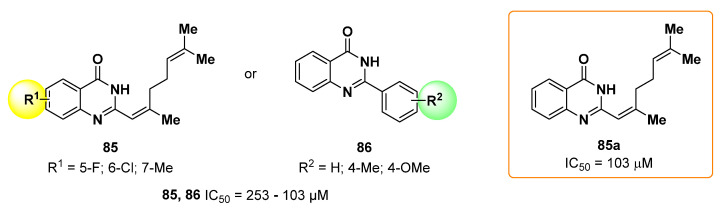
Quinazoline derivatives **85** and **86** in three-digit micromolar range of TYR inhibition.

Also quinazoline derivatives (**85**–**86**, Figure 55) were described as TYRIs by Wang et al. [100]. Some of the compounds did not show any activity whereas the IC_50_ values of the active molecules was between 103 and 253 µM, compared to the standard arbutin (**6**, IC_50_ = 180 µM). The most active derivative **85a** was a mixed-type and reversible TYRI.

### 2.20. Miscellaneous

**Figure 56:**
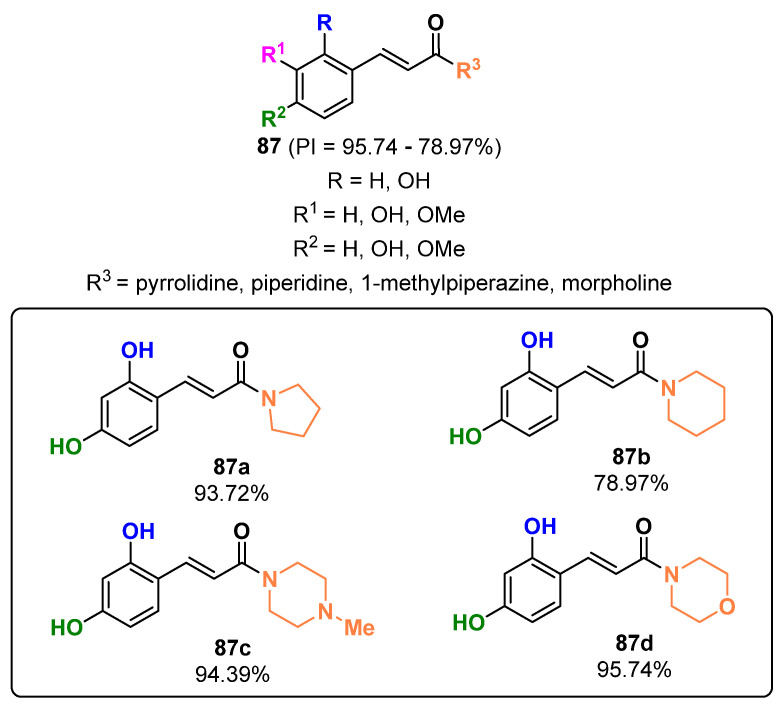
Cinnamide derivatives and their percentage of inhibition of monophenolase.

Cinnamic acid is a naturally occurring compound able to inhibit melanogenesis by inhibiting TYR activity with a low potency (IC_50_ = 2.10–0.21 mM) [101,102,103]. Several attempts have been carried out over the years, to improve the TYR inhibition of this natural active ingredient. One of these strategies included the synthesis of cinnamide analogs bearing a heterocyclic moiety. In this context, Ullah et al. reported the activity of several cinnamide derivatives (**87**, Figure 56) bearing different secondary cyclic amine, including pyrrolidine, piperidine, piperazine and morpholine [104,105]. Independently from the heterocyclic moiety, the most active compounds were those carrying a 2,4-dihydroxyphenyl portion **87a**–**87d**, which showed inhibition percentages against the monophenolase activity ranging between 95.74 and 78.97%, proving to be more active than kojic acid (**5**, PI = 20.57%), which was used as positive control. All four derivatives (**87a**–**87d**) were able to inhibit melanogenesis in α-MSH stimulated B16F10 melanoma cells by reducing TYR activity. Docking suggested that the hydroxyl groups might form H-bonds mainly involving Met280 and Asn260, while the phenyl ring could elicit hydrophobic contacts with Val283, Phe264 and Ala286.

**Figure 57:**
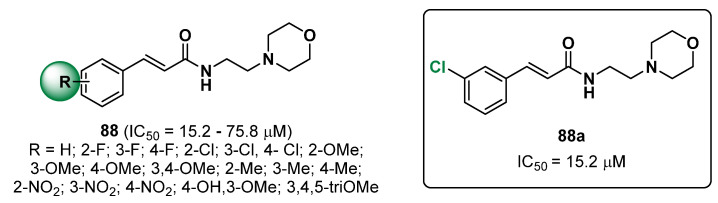
Morpholine containing cinnamides and inhibition of TYR thereof.

In 2019, Ghafary et al. described the synthesis and the biological evaluation of a new series of cinnamide derivatives containing a morpholine moiety (**88**, Figure 57) [106]. In this case, the choice of morpholine was based on the ability of this fragment to establish additional H-bonds and hydrophobic contacts with TYR binding site. Among the synthesized compounds, the mono-substituted derivatives carrying the NO_2_, 4-F, 3- and 4-Cl, 3-Me, the 3,4,5-triOMe and the non-substituted derivatives were able to inhibit the diphenolase activity with IC_50_ values between 15.2 and 75.8 μM. The most potent compound **88a** displayed an affinity similar to that of kojic acid (**5**, IC_50_ = 14.4 μM), which was used as standard, and a mixed-type inhibition mechanism. According to the docking studies, the amide bond of this class of inhibitors engaged H-bonds with some residues of the active site such as Glu256, His244 and His259, while the phenyl and morpholine ring are involved in hydrophobic interactions with the surrounding residues.

### 2.21. Azepines

**Figure 58:**
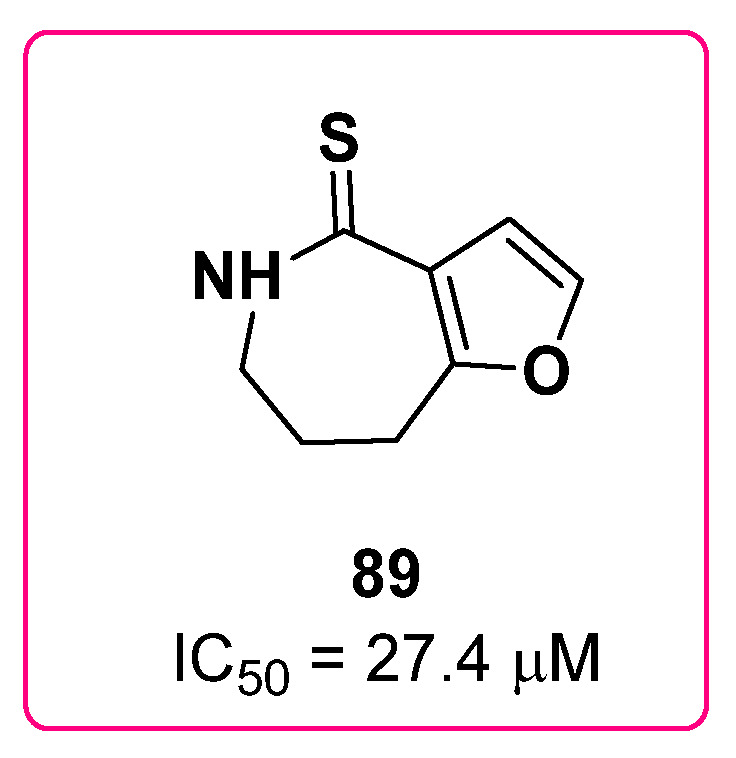
Chemical structure of T4FAT (**89**).

In 2019, Okajima et al. screened a library composed of 2000 compounds leading to the identification of the 5,6,7,8-tetrahydro-4*H*-furo[3,2-c]azepine-4-thione (T4FAT, **89**, Figure 58) as new inhibitor of melanogenesis in FSK-stimulated B16F10 cells [107]. This inhibitor is characterized by a thioamide moiety which revealed to be important for the inhibition of melanin production in cell-based assays. Moreover, it displayed an efficacy about thirty times higher than kojic acid (**5**), used as reference in inhibiting melanogenesis in B16F10 cells. However, when tested in vitro against AbTYR by using l-dopa (**2**) as substrate, T4FAT displayed a non-competitive inhibition mechanism with an IC_50_ value of 27.4 μM, showing a similar potency than kojic acid (**5**, IC_50_ = 35.7 μM).

### 2.22. Chromone Derivatives

**Figure 59:**
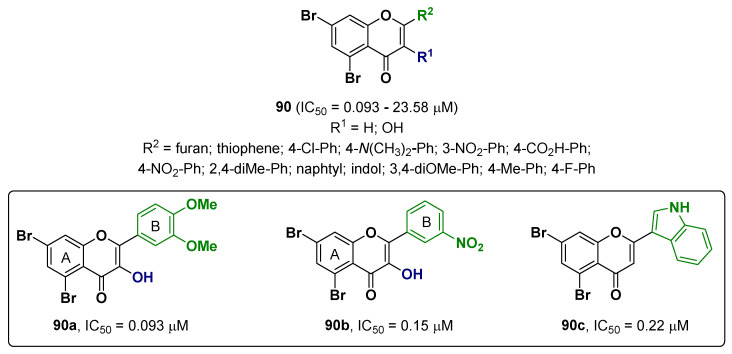
Chromone derivatives and their inhibition of TYR.

Chromone derivatives were reported in the literature as TYRIs by different groups [108,109,110]. A variety of 2-phenylchromones (**90**, Figure 59) was presented by Ahmed and co-workers [111]. The new derivatives were synthesized and their inhibitory activity towards TYR on diphenolase activity was evaluated. They showed IC_50_ values between 0.093 and 23.58 μM in comparison to the standard kojic acid (**5**, IC_50_ = 1.79 μM). Learnings from molecular docking allowed to understand that presence of bromine atoms on ring A increases the inhibitory potential of 2-phenylchromones. EDGs in *ortho*- and *para* positions of the phenyl ring B appear to be also favorable for the activity. An indole group present at the 2-position of the chromone motif since it fills large active pockets of the enzyme (Figure 59).

**Figure 60:**
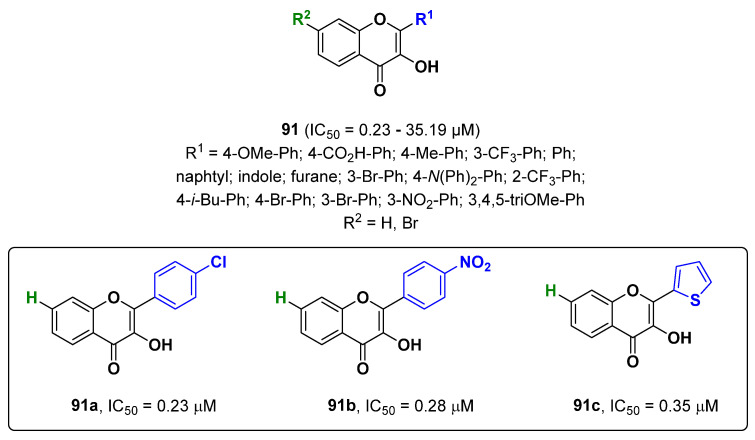
3-Hydroxyflavone derivatives **91** as TYRIs.

In 2021, Mirzaei et al. synthesized a new series of 3-hydroxyflavone derivatives (**91**, Figure 60) and evaluated their capabilities to inhibit TYR on diphenolase activity [110]. Some of the derivatives showed promising activity with IC_50_ values between 0.23 and 35.19 µM compared to kojic acid (**5**, IC_50_ = 1.79 µM). According to the biological data, the presence of substituents in the rings A and B seems favorable for a better activity. In particular, EWGs (i.e., NO_2_, Cl, CF_3_) in the *para* position of ring B gave the better activity meaning that these compounds fit and strongly interact with the active site of the enzyme. Highly hydrophilic (i.e., COOH) and hydrophobic (i.e., CH_3_ and *i*Bu) groups on both aryl rings (A and B) are responsible for lower activities because their interaction with the enzyme are not as pronounced. Furthermore, the replacement of aryl ring B with other heterocyclics, such as thiophene (**91c**, IC_50_ = 0.347 µM), increases the activity due to the ability of the sulfur atom to coordinate with the dinuclear copper in the active site of the enzyme (Figure 60). 

**Figure 61:**
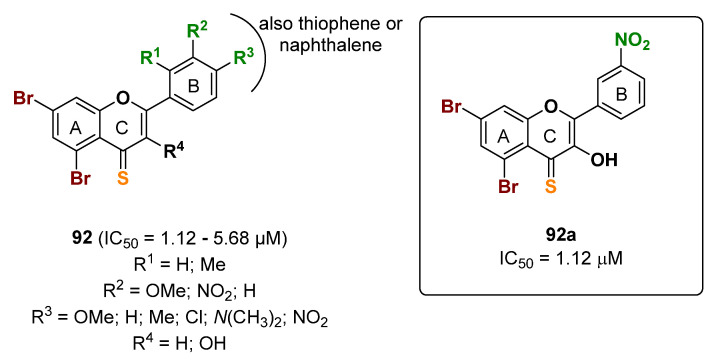
Thioflavones and thioflavonols derivatives and their activities on TYR inhibition.

The same research group, documented in 2022 a new series of thioflavones and thioflavonols (**92**) as potential TYRIs [112]. All the synthesized compounds (**92**, Figure 61) showed good inhibitory activities with IC_50_ values between 1.12 and 5.68 µM on diphenolase activity, compared to the standard kojic acid (**5**, IC_50_ = 12.6 µM). Derivative **92a** is a competitive inhibitor displaying the best inhibition with an IC_50_ value of 1.12 µM. The authors employed the 3-(4,5-dimethylthiazol-2-yl)-2,5-diphenyltetrazolium bromide (MTT) test for A375 human melanoma cells for evaluating the cytotoxicity of the most promising compounds. Derivative **92a** proved to be not cytotoxic. The pharmacophores decorated with the dibromo substitution on the A ring, which is fundamental for the activity, and in the B ring with EDGs, such as methyl, dimethyl amine, methoxy, or thiophene, or EWGs such as nitro or chloro, as shown in Figure 61, proved to be the most active derivatives.

### 2.23. Xanthone Derivatives

**Figure 62:**
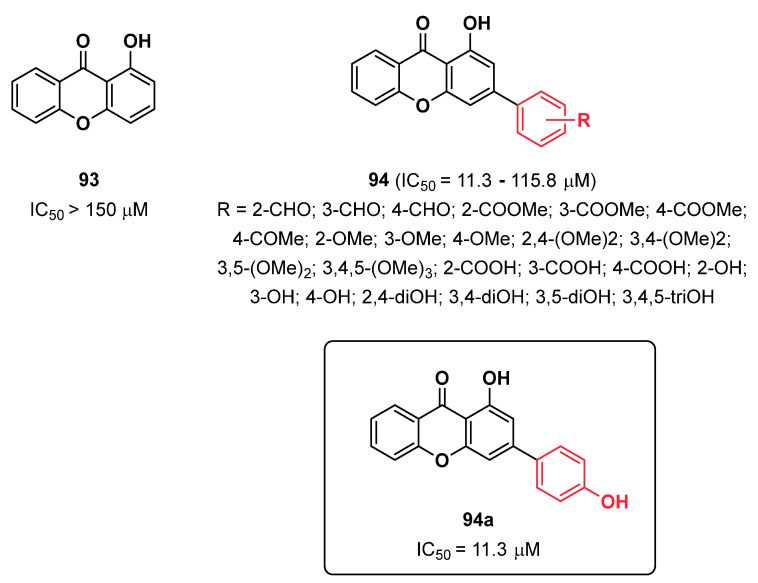
1-hydroxyl xanthone (**93**), and derived 3-aryl substituted xanthone derivatives and their TYR inhibitory potencies.

Xanthones are a class of oxygen containing compounds widely distributed in nature. Due to their multiple pharmacological activities, they emerged as attractive scaffold for the development of new therapeutics [113]. In this regard, the xanthone moiety was also exploited for the design of novel TYRIs. In 2019, Wu and co-workers reported a new series of 3-aryl substituted xanthone derivatives (**94**, Figure 62) that were screened against the diphenolase activity. All the compounds were able to inhibit the l-dopa (**2**) oxidation with IC_50_ values in the range of 11.3 to 115.8 μM [114], thus showing an improved activity if compared to the parent compound, 1-hydroxyl xanthone **93** (Figure 62, IC_50_ > 150 μM). The most potent derivative **94a** (IC_50_ = 11.3 μM) carried a 4-hydroxylphenyl moiety at the 3-position of the xanthone scaffold and displayed a slightly better activity than the positive control, kojic acid (**5**, IC_50_ = 17.3 μM). SAR analysis revealed that the presence of a single hydroxyl group was optimal for the activity, while an affinity loss was detected for the dihydroxy-substituted analogs. Overall, the substitution in *para* position resulted to be the most favorable for the inhibition. According to kinetic studies, compound **93** is a mixed-type inhibitor of TYR. Molecular docking suggested that the 4-hydroxyphenyl moiety might be inserted in the deep part of the active site close to the copper ions and it might engage hydrophobic contacts with Met280, Gly281, Ser282, Val283, and Ala286. Instead, the xanthone portion is oriented towards the surface of the pocket establishing hydrophobic interactions with Val248, Gly249, and Met257. 

**Figure 63:**
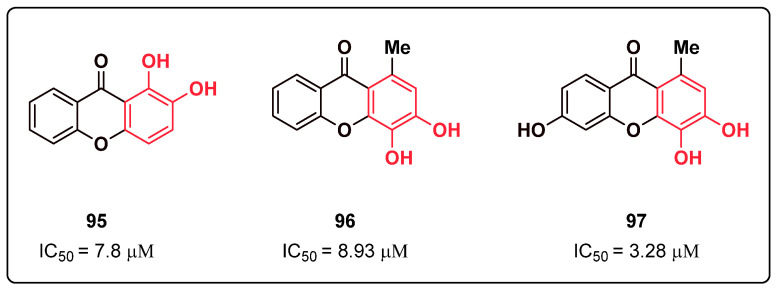
Catechol-containing-xanthone derivatives **95**–**97** and their IC_50_ values on TYR.

In 2020, Resende et al. described the anti-tyrosinase activity of three xanthone derivatives bearing a catechol moiety (**95**–**97**, Figure 63) [115]. These compounds were assayed by using l-tyrosine (**1**) as substrate and showed IC_50_ values in the range of 3.28 to 8.93 μM, thus proving to be more effective than the used reference, kojic acid (**5**, IC_50_ = 12.81 μM). Moreover, derivatives **95**–**97** were also tested in the DPPH antioxidant assay displaying a radical scavenging activity comparable to that of ascorbic acid (**7**) used as positive standard.

**Figure 64:**
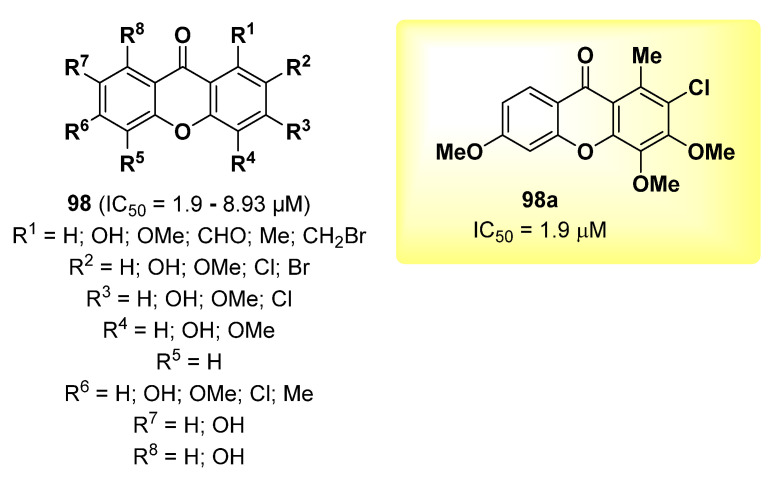
Further xanthone derivatives with promising inhibitory activity of TYR.

Pinto et al. synthesized a series of new xanthone derivatives (**98**, Figure 64) which showed promising activities as TYRIs [116]. Their activity was evaluated based on diphenolase activity with IC_50_ values between 1.9 and 8.93 µM in comparison to kojic acid (**5**, IC_50_ = 12.81 µM). Compound **98a** proved to be the best candidate with an IC_50_ value of 1.9 µM (Figure 64). SAR analysis suggested that bromine substitution slightly decreases the activity. Methyl substitution is favorable when a methoxy groups present in R^3^, R^4^, or R^6^, whereas it is not favorable when a hydroxy group is present. An aldehydic moiety is favorable when R^3^ and R^4^ are either methoxy or bromo methyl groups.

### 2.24. Coumarin Derivatives

**Figure 65:**
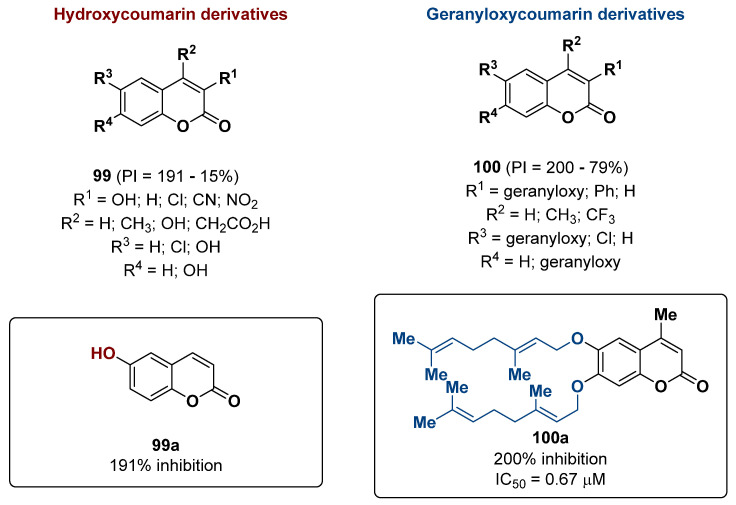
Coumarin derivatives **99** and **100** as TYRIs.

Roh reported in 2021 a series of hydroxy- and geranyloxy coumarin derivatives (**99**–**100**, Figure 65) as TYRIs [117]. The activity was evaluated on diphenolase activity and arbutin (**6**, % inhibition = 100%) was used as positive control. The presented compounds showed a percentage of inhibition between 191 and 15% for the hydroxy derivatives, whereas between 200 and 79% for the geranyloxy substituted coumarins, at a concentration of 0.4 µM (Figure 65). In general, the geranyloxy coumarin derivatives exhibited better activity than the hydroxy coumarin derivatives, suggesting the possibility of an application of these compounds as functional cosmetic ingredients.

**Figure 66:**
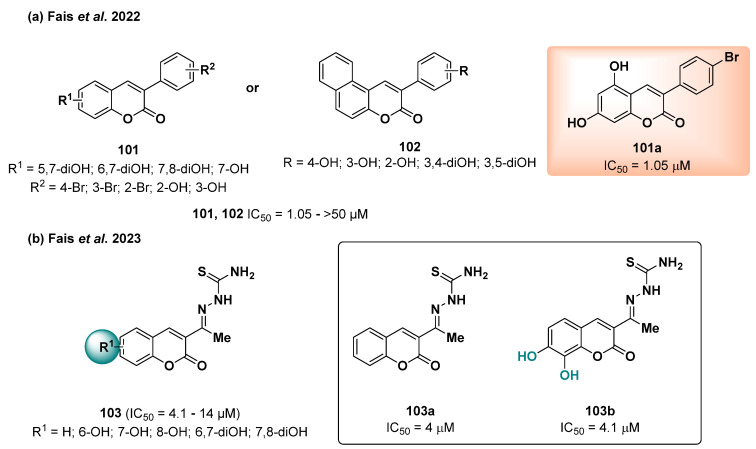
Coumarin derivatives with either aryl groups (**101** and **102**, (**a**) Fais et al. [118]) or thiosemicarbazone (**103**, (**b**) Fais et al. [119]) groups in the 3-position.

Fais and co-workers reported several coumarin derivatives as TYRIs [118]. In 2022, a series of phenyl and naphthyl compounds (**101**–**102**, Figure 66a) were described with IC_50_ values from 1.05 to >50 µM on diphenolase activity, compared to kojic acid (**5**, IC_50_ = 17.9 µM). The highest potency was observed for compound **101a** with an IC_50_ value of 1.05 µM (Figure 66a). SAR analysis revealed that the presence of a bromine atom in *meta*- or *para* position of the phenyl ring, attached to the 3-position of the coumarin, is important for the higher activity if it is combined with at least one hydroxyl group at position seven of the coumarin moiety. In 2023, the same group described a class of hydroxylated coumarin-based thiosemicarbazones (**103**, Figure 66b) as TYRIs and antioxidant agents [119]. All the synthesized compounds (**103**) showed a higher inhibitory activity compared to the positive control, which was kojic acid (**5**) with IC_50_ values between 4.1 and 14 µM. The best activity was observed for derivatives **103a** and **103b** with IC_50_ value of 4 and 4.1 µM, respectively, which are competitive inhibitors (Figure 66b). For compounds bearing a hydroxy group attached to the coumarin ring, a mixed-type inhibition was observed. The most promising compound (**103b**) was found to be not cytotoxic on B16F10 cells. Molecular docking was performed, showing that the presence of a hydroxy group significantly alters the binding mode in the active site of the enzyme. Indeed, the hydroxy groups of compound **103b** are oriented towards the catalytic site, whereas for compound **103a** the thiosemicarbazone moiety might interact with the copper ions.

**Figure 67:**
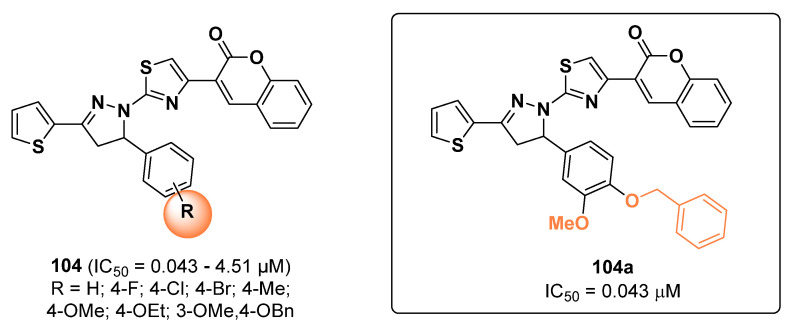
Coumarin-based thiophenyl-pyrazolylthiazole hybrids which show good activity in TYR inhibition.

Kim and co-workers described a new class of coumarin-based thiophenyl-pyrazolylthiazole hybrids (**104**, Figure 67) [120]. All the synthesized compounds possess strong inhibitory activities, with IC_50_ between 0.043 and 4.51 µM on diphenolase activity, whereas kojic acid (**5**, IC_50_ = 18.52 µM) served as reference. The most active compound **104a** has an IC_50_ of 0.043 µM and is a non-competitive inhibitor. Furthermore, all the compounds (**104**) showed a good antioxidant activity against DPPH and were found to be not cytotoxic on B16F10 melanoma cells.

### 2.25. Kojic Acid Derivatives

**Figure 68:**
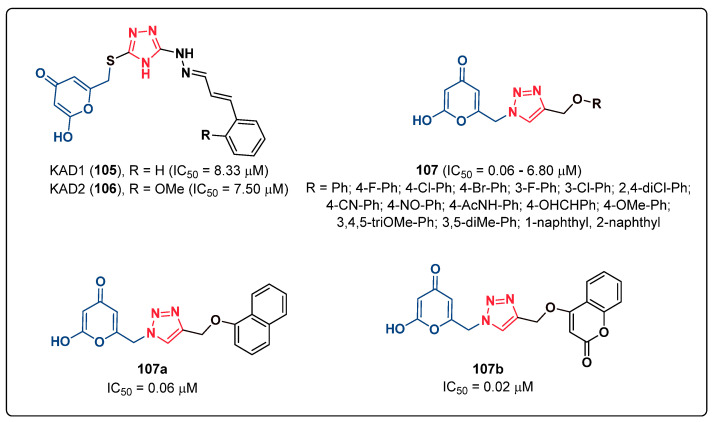
Triazole-based kojic acid derivatives **105**–**107** and their inhibitory profile on TYR.

Kojic acid (**5**) is a fungal metabolite produced by *Aspergillus* and *Penicillium* species. It is a well-known TYRI, often used as positive control to estimate the inhibitory potential of novel anti-tyrosinase agents [121]. Kojic acid (**5**) is able to inhibit the monophenolase activity in a competitive manner by chelating the copper center. Moreover, it also blocks the diphenolase activity of the enzyme with a mixed-type inhibition mechanism [122]. It demonstrated also promising antioxidant capacity with photoprotective, anti-inflammatory, and pain-relieving actions [123]. However, it has some side effects since it is an irritating agent with high cytotoxicity and instability in storage [124]. In order to improve its stability, absorption, and hypopigmented effect, different structural modifications were performed [125], and several derivatives were designed to enhance its inhibitory effect on TYR. One of the adopted strategies included the linkage of kojic acid (**5**) with several aryl and heteroaryl moieties by a triazole linker. Within this context, Chen et al. described the synthesis and the biological evaluation of two kojic acid derivatives, KAD1 (**105**, Figure 68) and KAD2 (**106**, Figure 68) containing the phenyl allylidene hydrazine portion linked to kojic acid (**5**) by a 1,2,4-triazole-3-thiol ring. KAD1 and KAD2 inhibited the diphenolase activity with IC_50_ values of 8.33 μM and 7.50 μM, respectively, showing a higher potency than the reference kojic acid (**5**, IC_50_ = 19.50 μM) [126]. Kinetic studies revealed that both compounds act as mixed-type inhibitors [127]. Moreover, KAD2 reduced melanogenesis in B16F10 cells decreasing TYR activity and reducing the expression of different proteins involved in the melanogenic process such as TYR, TRP1, TRP2, p-PKA and p-CREB. It is noteworthy that KAD2 also suppressed melanogenesis and TYR activity in zebrafish used as vertebrate model to study the depigmenting effect of anti-melanogenic compounds [126]. 

Ashooriha and co-workers reported the anti-tyrosinase activity of some 1,2,3-triazole-based kojic acid derivatives in which the triazole is conjugated with different aryloxy moieties (**107**, Figure 68) [128]. All the resulting compounds (**107**) proved to be active against the diphenolase activity with IC_50_ values in the range of 0.06–6.80 μM, thus showing a greater inhibition than kojic acid (**5**, IC_50_ = 9.28 μM). SAR analysis unveiled that the substitution pattern of the aryloxy portion affected the inhibitory activity against AbTYR. The most active derivative was the 1-naphthyloxy analog **107a** which was characterized by an IC_50_ value of 0.06 μM. According to the Lineweaver–Burk plot analysis, **107a** inhibited TYR mostly in an uncompetitive manner. Moreover, **107a** showed metal chelating ability especially towards Cu^2+^ ions. Docking studies suggested that **107a** might bind to the enzyme active site with the 3-hydroxy-4-pyrone moiety situated close to the copper ions, while the triazole portion might engage H-bonds with His244. Instead, the naphthyl could establish hydrophobic contacts with Asn81, Cys83 and Thr324. In 2020, the same research group designed a new series of triazolo-based kojic acid analogs conjugated with different natural products endowed with anti-tyrosinase activity such as umbelliferone, sesamol, thymol, carvacrol, eugenol, isoeugenol, vanillin and isovanillin [129]. All the derivatives were tested against tyrosinase using l-dopa (**2**) as substrate, displaying IC_50_ values between 0.02 and 3.75 μM, thus being more potent than kojic acid (**5**, IC_50_ = 9.28 μM). The derivative carrying the 4-hydroxycoumarin (**107b**), see Figure 68, was the most active and it exhibited a mixed-type inhibition mechanism. Furthermore, **107b** decreased the melanin content in B16F10 melanoma cells, without relevant toxicity. Copper binding studies highlighted the ability of **107b** to interact with copper ions. Similar to **107a**, docking simulations showed that the pyranone moiety is located close to the copper center with the carbonyl group coordinating the metal ions, while the triazole moiety might form H-bonds with His244. Finally, the coumarin portion might be involved in hydrophobic interactions with Glu322, Cys83, Asn81 and Thr324. 

**Figure 69:**
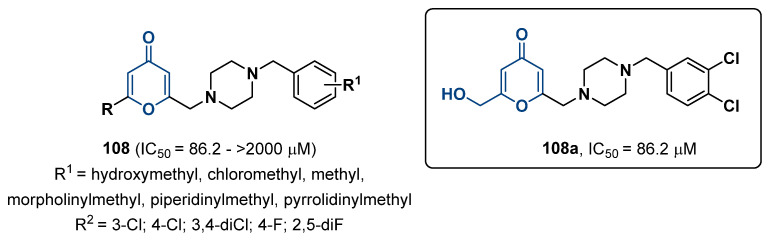
Kojic acid derivatives containing a benzylpiperazine portion (**108**) and their TYR inhibition.

Karakaya et al. synthesized and tested several kojic acid derivatives (**108**, Figure 69) containing a substituted benzylpiperazine portion [59]. The authors also explored the 6-position of the pyranone ring by replacing the hydroxymethyl group with chloromethyl, methyl, morpholinyl methyl, piperidinyl methyl, and pyrrolidinyl methyl moieties. The tyrosinase inhibitory activity of the obtained derivatives was determined by an assay with l-dopa (**2**) as substrate. The most active compound (**108a**) of the series had a hydroxymethyl group in the 6-position, while the phenyl ring was decorated with chlorine atoms in the *para*- and one of the *meta*-positions. This compound inhibited TYR with an IC_50_ value of 86.2 μM, thus proving to be almost five-fold more active than the reference compound kojic acid (**5**, IC_50_ = 418.2 μM) (Figure 69). SAR analysis revealed that the substituents on the phenyl ring affected the inhibitory activity on TYR, whereas the 3,4-dichloro substitution pattern proved to be the most favorable of the tested patterns. Docking analysis predicted that compound **108** might interact with the active site of TYR by coordinating the metal ions through the hydroxymethyl moiety, while the 2,4-dichlorobenzylpiperazine portion could be involved in H-bonds and hydrophobic interactions.

**Figure 70:**
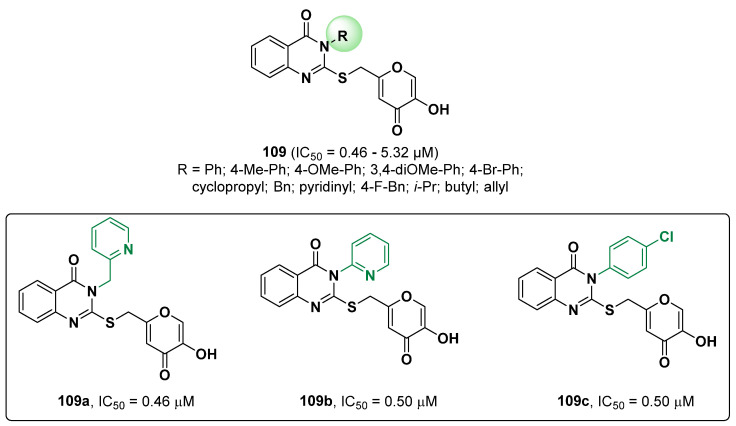
Thioquinazolinones conjugated to kojic acid derivatives (**109**) showing medium 3-digit nanomolar activity as TYRIs.

Khoshneviszadeh and co-workers reported a variety of thioquinazolinones conjugated to kojic acid derivatives (**109**, Figure 70) as potential anti-melanogenesis agents and TYRIs [130]. The inhibitory activity was evaluated using l-dopa (**2**) as substrate with IC_50_ values between 0.46 and 5.32 µM showing an improved activity in comparison to the positive control, which was kojic acid (**5**, IC_50_ = 9.30 µM). Considering the SAR, the presence of EDGs in the aromatic portions attached to the nitrogen had negative effects on TYR inhibition, but the enhancement of hydrophilicity had more positive roles. On the other hand, EWGs substitution increased the activity, especially in *para* position. A better potency was exerted by the chlorine substituent in comparison to the bromine one. Elongation of the alkyl linker between the quinazolinone and aromatic portions attached to the nitrogen improved the activity in most cases (Figure 70). Derivatives **109a** and **109b** showed also good potency against the B16F10 cell line to reduce the melanin content with limited toxicity against malignant cells.

**Figure 71:**
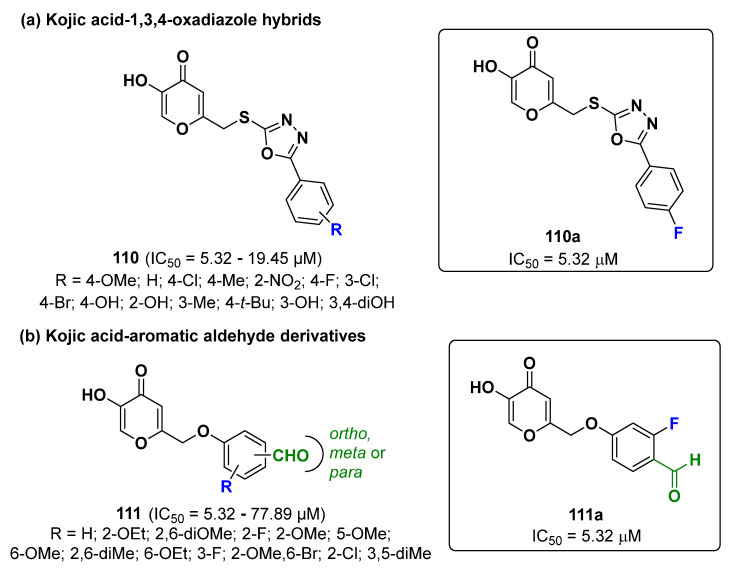
Kojic acid derivatives.

Peng et al. reported in 2023 a series of new kojic acid-1,3,4-oxadiazole hybrids (**110**, Figure 71a) as TYRIs [131]. The IC_50_ values obtained for all the synthesized compounds (**110**) range from 5.32 to 19.45 µM using l-dopa (**2**) as substrate, compared to kojic acid (**5**, IC_50_ = 49.77 µM). Studies regarding the mechanism of action were carried out on the most active compound **110a**, showing that it is not only able to bind the copper ions in the active region of TYR but also to change its secondary structure. It also showed anti-browning effect and no cytotoxicity. These observations can serve as impetus for further studies based on this promising pharmacological profile. The same group described a class of compounds, which combines kojic acid (**5**) with aromatic aldehydes (**111**, Figure 71b) as TYRIs, showing IC_50_ values between 5.32 and 77.89 µM on diphenolase activity, compared to kojic acid (**5**, IC_50_ = 48.05 µM) [132]. Compound **111a** is a non-competitive inhibitor with an IC_50_ value of 5.32 µM possessing good anti-browning effect (Figure 71b).

The implementation of heterocyclic moieties proved to be well tolerated in potent AbTYRIs. Important for the activity seem to be the substitution patterns of several aryl rings with substituents of different nature. The best activities were observed when hydroxyl-, methoxy-, nitro-, -methyl groups, or halogens were present in the molecules. Overall, there was no functional group that proved to be the best substituent in general for AbTYR inhibition, but from each case some learnings can be drawn. These insights should be taken into consideration when novel inhibitors are designed.

## 3. Human Tyrosinase Inhibitors

**Figure 72:**
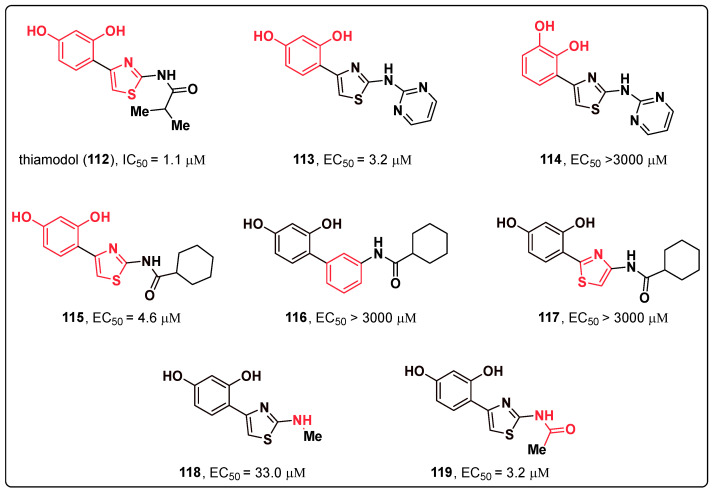
hTYRIs (**112**–**119**) bearing the thiazolyl resorcinol moiety.

Over the years, AbTYR has been widely employed to evaluate the depigmenting activity of potential skin whitening agents. However, many studies highlighted the differences between the AbTYR and hTYR isoforms which require diverse structural motifs for their inhibition [133]. In comparison to AbTYR, the number of reported hTYRIs is limited [134]. A study, conducted by Mann and co-workers, pointed out that some well-known TYRIs employed in topical formulations had lower affinities towards hTYR if compared to the fungal isoform [135]. To overcome these limitations, the same research group screened a library of 50,000 compounds by using the recombinant hTYR. Thiamidol (**112**, *iso*-butylamido thiazolyl resorcinol, Figure 72) proved to be most potent hTYRI identified in this screening campaign with an IC_50_ value of 1.1 μM. Thiamidol was tested on AbTYR as well, showing a lower affinity as displayed by the corresponding IC_50_ value of 108 μM. Furthermore, thiamidol was able to inhibit melanogenesis in melanocyte cultures (IC_50_ = 0.9 µM) revealing to be more effective than hydroquinone (IC_50_ = 16.3 μM) used as reference. Clinical studies disclosed the in vivo efficacy of this compound. The application twice per day in a formulation containing the 2% of the active ingredient, led to a significant whitening effect of age spots after four weeks. The putative binding mode of thiamidol into hTYR active site was probed by computational studies performed on a homology model of the protein. The outcomes revealed that the resorcinol moiety is arranged in close proximity to the metal center with the hydroxyl group in the *ortho* position, engaging H-bonds with Ser380. The thiazole ring is mainly involved in hydrophobic interactions while another H-bond was observed between the carbonyl group and Ser375. Later that year, the same research group, reported new insights into the SAR of thiazolyl resorcinol derivatives as hTYRIs [136]. Specifically, the authors synthesized and tested a new series of compounds bearing the aforementioned scaffold that features an amine or amide moiety. The biological data revealed that the thiazolyl resorcinol moiety is crucial for the activity. The replacement of resorcinol with other hydroxylation patterns impaired the activity, as shown for derivative **114** (Figure 72) in which the introduction of the catechol ring led to a loss of activity in comparison to the resorcinol analogue **113** (Figure 72). Similarly, the substitution of the thiazole with other aromatic or heteroaromatic rings as well as inverting the position of the thiazole had a detrimental effect on the inhibition, as exemplified by compounds **116** (Figure 72) and **117** (Figure 72), respectively, for which an affinity reduction was observed when compared to analog **115**, bearing the dihydroxyphenyl moiety on the 4-position of the thiazole ring. Substitutions at the 2-amino group were tolerated better. Overall, it was observed that the amides were more active than the corresponding amines, as seen for derivatives **118** (Figure 72) and **119** (Figure 72). Moreover, small substituents positively affected the activity, while large and hydrophobic groups usually decreased the inhibition respect to more hydrophilic substituents of the same size.

**Figure 73:**
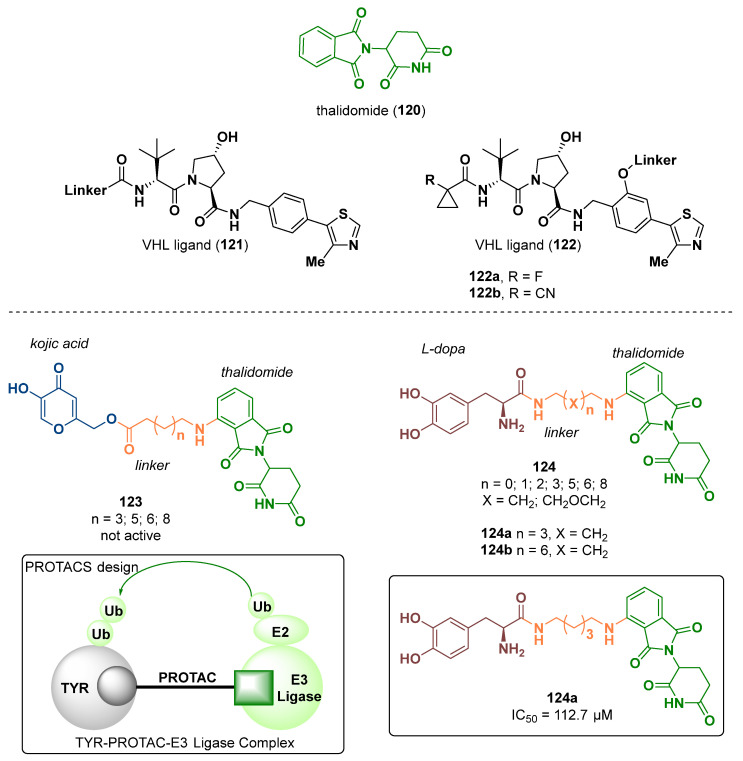
PROTACs as human tyrosinase degraders.

In 2021, Tang et al. identified a series of proteolysis-targeting chimeras (PROTACs) as degraders of hTYR [137] (Figure 73). PROTACs are chemical compounds which provoke the degradation of specific proteins by the ubiquitin-proteasome system (UPS) [138]. They are characterized by having a portion that binds to a protein of interest, a linker and an E3 ubiquitin ligase binder. Considering the mechanism of action of a tyrosinase-targeting PROTAC, it binds to the E3 ligase and the tyrosinase at the same time. In this way the ubiquitination of the targeted TYR occurs by E3 ligase, which transfers ubiquitin units to the protein. The transfer is favored due to the close spatial arrangement. The ubiquitinylated protein is then identified by UPS and degraded. The well-established thalidomide (**120**) was used as E3 ligase binding portion, targeting cereblon [139]. Another popular and widely explored E3 Ligase would be the von Hippel-Lindau E3 ligase, which can be targeted by implementation of VHL ligands (**121**, **122**) [140,141]. Kojic acid (**5**) and thalidomide (**120**, Figure 73) were chosen as main constituents of PROTACs connected by linkers of different length. However, the designed compounds (**123**, Figure 73) did not show promising activity and the authors decided to replace kojic acid (**5**) with l-dopa (**2**). A new series of derivatives (**124**, Figure 73) was synthesized, and the best result was obtained for the compounds containing 5 (**124a**) and 8 (**124b**), respectively, methylene groups in the linker portion of the molecule, see Figure 73. Furthermore, hydrophobic linkers were found to be fundamental for the activity. The authors evaluated both the tyrosinase-degradation level by using human A375 cells and the inhibitory activity on hTYR. The degrader **124a** was selected because of the best tyrosinase-degrading ability and it showed a DC_50_ value (the concentration for 50% protein degradation) of 50 µM, the maximal level of degradation (Dmax) of 61% at concentration of 100 µM, and an IC_50_ value of 112.7 µM on hTYR. Thus, it could both degrade and inhibit TYR. The cytotoxicity of **124a** was also evaluated by using A375 and HEK293 cells. The half-cytotoxic concentration (CC_50_) against both cells resulted to be higher than 700 µM. Additional testing revealed that **124a** is less cytotoxic than the reference, hydroquinone (**8**), proving to be safe for further applications. Indeed, it can reduce melanin production in human cell lines, showing a better efficacy in comparison to kojic acid (**5**) and l-dopa (**2**). In vivo studies were carried out for compound **124a,** using a zebrafish model [142], which showed a depigmenting ability 3.3 and 5.1 times higher of positive compounds kojic acid (**5**) and l-dopa (**2**), respectively.

**Figure 74:**
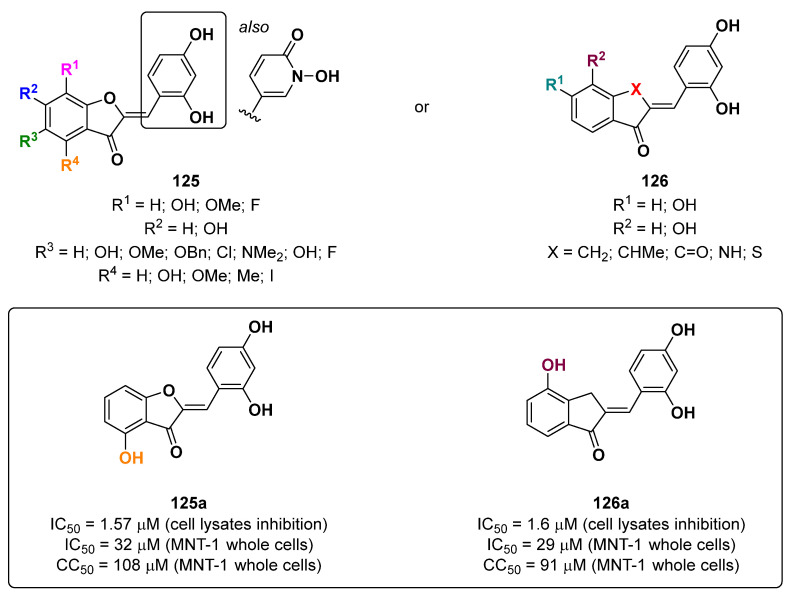
Resorcinol-based hemiindigoid derivatives.

In 2023, Haudecoeur et al. reported a class of resorcinol-based hemiindigoid derivatives (**125**–**126**, Figure 74) as hTYRIs [143]. Their cell-based activity was evaluated on both human melanoma cell lysates and purified hTYR assays. Considering the human melanoma MNT-1 cell lysates inhibition, IC_50_ values between >100 and 1.57 µM were obtained, compared to the positive control rucinol (**9**, IC_50_ = 8.3 µM). For the two most active compounds **125a** (IC_50_ = 1.57 µM) and **126a** (IC_50_ = 1.6 µM), the MNT-1 whole cells melanogenesis inhibition, the cytotoxicity, and the kinetic parameters using a purified hTYR inhibition assay were measured and compared to kojic acid (**5**, IC_50_ = 15,000 µM; EC_50_ = 34,000 µM). Derivative **125a** showed an IC_50_ value of 32 µM and a EC_50_ value of 108 µM on MNT-1 whole cells assay, whereas **126a** presents an IC_50_ value of 29 µM and a EC_50_ value of 91 µM (Figure 74). Thus, for two derivatives, **125a** and **126a**, an excellent inhibition effect was observed in both human melanoma cell lysates and purified hTYR assays, showing promising results for further studies.

Considering the SAR of the reported hTYRIs, the presence of nitrogen-, sulfur-, and oxygen-containing heterocycles led to promising results for the development of more potent inhibitors. The substitution of phenyl rings with one or two hydroxyl groups seems to give the best potencies within the respective sets of inhibitors. 

## 4. Conclusions

In conclusion, a comprehensive review of the recently described synthetic TYRIs bearing a heterocyclic structure is reported. TYR is a copper-containing enzyme which is widely present in nature. It catalyzes two consecutive oxidation steps in the biosynthesis of melanin. However, anomalies in the production of melanin can lead to serious diseases. In humans, the abnormal lack of melanin could be responsible of albinism; on the other hand, an excessive accumulation of melanin can cause disorders related to hyperpigmentation. For these reasons, TYRIs are potential skin-whitening agents and antimelanogenic substances for melanoma treatment. Due to the side effects related to the actual substances used in skin disorders, e.g., kojic acid (**5**) and arbutin (**6**), the development of new molecules with better pharmacological profile is needed. Among the inhibitors described so far, many possess heterocyclic functionalities which are fundamental for the biological activity. Thus, this review based around heterocycle-bearing TYRIs can be considered useful and inspiring for the scientific community in order to design new generations of molecules able to inhibit or even degrade tyrosinase. It can be expected that, based on learnings that can be drawn from this review, novel TYRIs will be presented to the scientific community and hopefully to patients in need of them.

## Data Availability

Not applicable.

## Supplementary Materials

The following supporting information can be downloaded at: https://www.mdpi.com/article/10.3390/ijms24109097/s1.
