# Supplementary material for: Heterocyclic Compounds as Synthetic Tyrosinase Inhibitors: Recent Advances
Source: Int J Mol Sci. 2023 May 22;24(10):9097. doi: 10.3390/ijms24109097 (PMC10218907; doi:10.3390/ijms24109097)
Supplement: Supplementary file 1 [file ijms-24-09097-s001.zip › ijms-2389511-supplementary.pdf]

Table S1. Carbazole derivatives, see **Figure 3** (TYRIs bearing the carbazole scaffold).

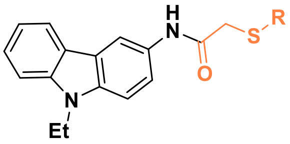

10

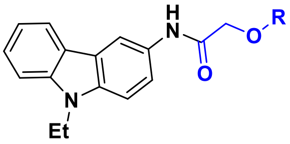

11

Compound	R	*K*i (µM ± SEM)
**10a**	2-benzimidazole	1.64
**10b**	2-thiazole	3.46
**10c**	2-benzoxazole	4.34
**10d**	2-benzothiazole	6.87
**11a**	3-nitrobenzene	5.41
**11b**	4-ethylbenzene	6.73
**11c**	4-chlorobenzene	7.48
kojic acid (**5**)		4.43

Table S2. 3-Hydroxy-1*H*-pyrrol-2(5*H*)-one derivatives, see **Figure 4** (3-Hydroxy-1*H*-pyrrol-2(5*H*)-one derivatives and their inhibitory activity).

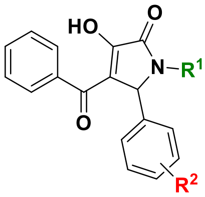

12

Compound	R^1	R^2	IC_{50} (µM ± SE)
**12a**	phenyl	H	6.98
**12b**	phenyl	2-F	8.12
**12c**	phenyl	2-Cl	-
**12d**	phenyl	3-Br	-
**12e**	phenyl	4-NO_2	-
**12f**	phenyl	4-OMe	-
**12g**	2-propenyl	2-F	9.77
**12h**	2-propenyl	2-Cl	-
**12i**	2-propenyl	4-Cl	-
**12j**	2-propenyl	2-Br	-
**12k**	2-propenyl	3-Br	-
**12l**	2-propenyl	4-NO_2	26.73
**12m**	2-propenyl	4-OMe	-
**12n**	2-F-phenyl	H	-
**12o**	4-Cl-Phenyl	H	-
**12p**	4-Cl-Phenyl	4-OMe	-
**12q**	4-Br-Phenyl	H	-
**12r**	4-ethyl-phenyl	H	-
**12s**	4-ethyl-phenyl	2-F	-
kojic acid (**5**)			18.56

Table S3. 2-Cyanopyrrole derivatives, see **Figure 5** (Derivatives of 2-cyanopyrrole and their inhibitory activities).

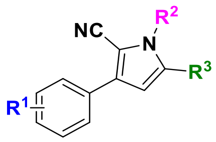

13

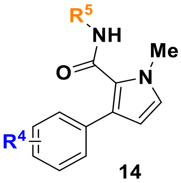

14

Compound	R^1	R^2	R^3	R^4	R^5	IC_{50} (μM)
**13a**	2-vinyl	Me	H	-	-	0.97
**13b**	2-OMe; 5-Br	Me	H	-	-	2.11
**13c**	2,3-diCl	Me	H	-	-	4.83
**13d**	H	Me	H	-	-	7.42
**13e**	4-Br	Me	H	-	-	8.17
**13f**	2-Br	Me	H	-	-	8.47
**13g**	2-F	Me	H	-	-	8.72
**13h**	4-CF_3	Me	H	-	-	12.44
**13i**	3-Br	Me	H	-	-	16.52
**13j**	3-F	Me	H	-	-	21.43
**13k**	2-OMe	Me	H	-	-	23.57
**13l**	4-OMe	Me	H	-	-	89.15
**13m**	H	Et	Me	-	-	4.46
**13n**	4-Cl	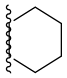		-	-	5.06
**13o**	4-Cl	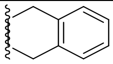		-	-	5.57
**13p**	4-Cl	benzyl	H	-	-	80.46
**13q**	4-Cl	Me	phenyl	-	-	87.32
**14a**	-	-	-	3-Br	H	>200
**14b**	-	-	-	3-Br	CO_2*t*-Bu	>200
**14c**	-	-	-	4-Br	H	>200
**14d**	-	-	-	4-Br	CO_2*t*-Bu	>200
**14e**	-	-	-	3-F	CO_2*t*-Bu	>200
**14f**	-	-	-	H	CO_2*t*-Bu	>200
**14g**	-	-	-	4-CF_3	CO_2*t*-Bu	>200
**14h**	-	-	-	2-F	CO_2*t*-Bu	>200
kojic acid (**5**)						28.72

Table S4. Indole-carbohydrazide derivatives, see **Figure 6** (Indole-carbohydrazide derivatives).

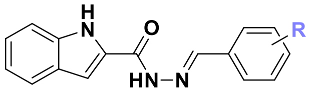

15

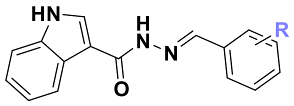

16

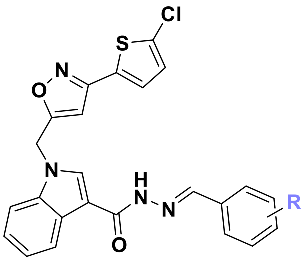

17

Compound	R	IC_{50} (μM)
**15a**	4-OH	0.070
**15b**	2,4-diCl	61.52

**15c**	3-F	69.66
**15d**	4-OMe	72.94
**15e**	3-Cl	78.52
**15f**	H	79.43
**15g**	2,3-diOH	80.72
**15h**	2,4-diOH	-
**16a**	4-OH	0.072
**16b**	2,4-diCl	94.84
**16c**	3-F	-
**16d**	3-Cl	-
**16e**	4-OMe	-
**16f**	H	-
**16g**	2,4-diOH	-
**16h**	2,3-diOH	-
**17a**	4-OH	0.19
**17b**	4-OMe	2.96
**17c**	2,4-diOH	3.30
**17d**	2,3-diOH	3.40
**17e**	2,5-diOH	4.62
**17f**	3-Cl	47.42
**17g**	H	95.06
**17h**	3-F	-
**17i**	2,3-diOMe	-
kojic acid (**5**)		9.28

Table S5. *N*-Phenylacetamide-oxindole-thiosemicarbazide hybrids, see **Figure 7** (*N*-Phenylacetamide-oxindole-thiosemicarbazide hybrids (**18**) and their activities).

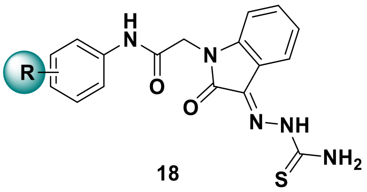

18

Compound	R	IC_{50} (μM)
**18a**	2-Me; 4-NO_2	0.80
**18b**	2-F	0.84
**18c**	4-Br	0.89
**18d**	4-F	0.97
**18e**	4-Me	1.06
**18f**	4-Cl	1.21
**18g**	3-F	1.44
**18h**	2-Me	1.73
**18i**	4-NO2	2.14
**18l**	2-Cl	2.17
**18m**	H	2.30
**18n**	2,3-diCl	2.47
**18o**	3-Cl	2.85
**18p**	4-OMe	3.40
**18q**	2,6-diCl	3.88
kojic acid (**5**)		36.32

Table S6. Aryl pyrazole derivatives, see **Figure 8** (TYRIs bearing an aryl pyrazole moiety).

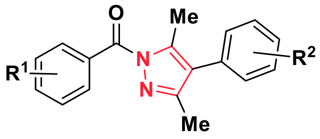

19

Compound	R^1	R^2	IC_{50} (μM)
**19a**	2,4-diOMe	4-NH_2	1.56
**19b**	3-Me	4-NO_2	2.12
**19c**	4-NO_2	4-OMe	2.31
**19d**	2,4-diOMe	4-NO_2	3.37
**19e**	4-OMe	4-NH_2	6.46
**19f**	4-Me	4-NH_2	7.75
**19g**	4-NO_2	3-NH_2	7.89
**19h**	3-Me	4-OMe	10.07
**19i**	4-OMe	4-NO_2	15.13
**19j**	4-Me	4-OMe	16.59
**19k**	3-Me	4-NH_2	19.53
**19l**	4-NO_2	4-NH_2	19.65
kojic acid (**5**)			16.05

Table S7. 1,2,3-Triazolo-coumarino[4,3-*c*]pyrazole derivatives, see **Figure 9** (Inhibitory effects of 1,2,3-triazolo-coumarino[4,3-*c*]pyrazole derivatives).

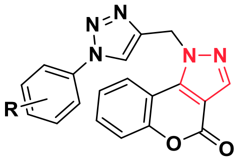

20

Compound	R	PI (%)
**20a**	H	85.0
**20b**	2,4,6-triCl	72.0
**20c**	3-Me	66.0
**20d**	4-Cl	46.0
**20e**	phenyl	-
kojic acid (**5**)		85.2

Table S8. Pyrazole-resorcinol derivatives, see **Figure 10** (Pyrazole-resorcinol derivatives with promising biological profiles).

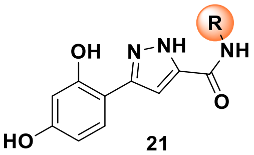

21

Compound	R	IC_{50} monophenolase (μM)	IC_{50} diphenolase (μM)
**21a**	3,4,5-triOMe-phenyl	0.006	0.64

**22b**	4-CO_2Me-phenyl	0.02	0.45
**22c**	4-OMe-phenyl	0.03	-
**22d**	4-F-phenyl	0.03	0.97
**22e**	naphthalen-2-yl	0.04	2.88
**22f**	2-Me-phenyl	0.06	3.55
**22g**	4-Cl-phenyl	0.06	1.69
**22h**	naphthalen-1-yl	0.07	6.19
**22i**	2-OMe-phenyl	0.07	11.25
**22j**	2,6-diOMe-phenyl	0.08	10.70
**22k**	4-Br-phenyl	0.11	4.21
**22l**	4-Me-phenyl	0.11	2.23
**22m**	4-CN-phenyl	0.12	6.51
**22n**	4-*i*Pr-phenyl	0.14	0.14
**22o**	3-OMe-phenyl	0.26	10.14
**22p**	3-Me-phenyl	0.33	0.45
**22q**	phenyl	0.33	5.23
**22r**	6-methylpyridin-2-yl	0.73	6.86
**22s**	3-F-phenyl	0.79	7.23
kojic acid (**5**)		21.58	17.76

Table S9. Benzimidazole-1,2,3-triazole derivatives, see **Figure 12** (Benzimidazole-1,2,3-triazole hybrids).

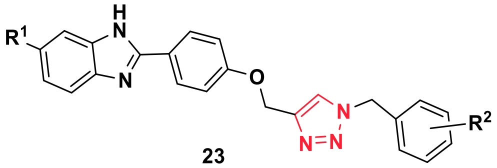

23

Compound	R^1	R^2	IC_{50} (μM)
**23a**	H	3,4-diCl	9.42
**23b**	H	4-Br	10.34
**23c**	H	2-F	45.22
**23d**	H	4-F	46.89
**23e**	H	4-NO_2	48.00
**23f**	H	Phenyl	>50
**23g**	H	4-Me	>50
**23h**	H	2-Cl	>50
**23i**	H	2,3-diCl	>50
**23j**	Me	4-F	29.31
**23k**	Me	H	43.30
**23l**	Me	2-Cl	>50
**23m**	Me	3-Cl	>50
**23n**	Me	2,3-diCl	>50
**23o**	Me	3,4-diCl	>50
**23p**	Me	4-Br	>50
**23q**	Me	4-NO_2	>50
**23r**	Me	4-Me	>50
koji acid (**5**)			9.28

Table S10. 1,2,4-Triazol-3-ylthio-*N*-phenyl acetamide derivatives, see **Figure 13a** (1,2,4-Triazol-3-ylthio-*N*-phenyl acetamide derivatives (**24**) containing the 2-aminothiazole moiety (a) in the position five of the triazole ring).

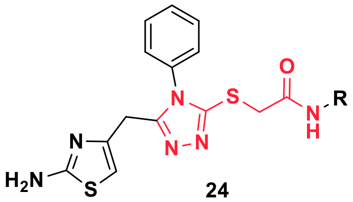

24

Compound	R	IC$_{50}$ (μM)
**24a**	2-Me-phenyl	0.0156
**24b**	ethyl phenyl	0.0161
**24c**	2,6-diMe-phenyl	0.0513
**24d**	2,4-diMe-phenyl	0.0547
**24e**	2,5-diMe-phenyl	0.0589
**24f**	2-Et-phenyl	0.0652
**24g**	3,4-diMe-phenyl	0.1133
**24h**	4-Et-phenyl	0.1224
**24i**	phenyl	0.1330
**24j**	4-OEt-phenyl	0.1726
**24k**	2-Et,6-Me-phenyl	0.1943
**24l**	3,5-diMe-phenyl	0.2465
**24m**	benzyl	1.0108
**24n**	2,3-diMe-phenyl	2.0181
kojic acid (**5**)		16.8320

Table S11. 1,2,4-Triazol-3-ylthio-*N*-phenyl acetamide derivatives, see **Figure 13b** (1,2,4-Triazol-3-ylthio-*N*-phenyl acetamide derivatives containing a phenyl ring (**25**) (b) in the position five of the triazole ring).

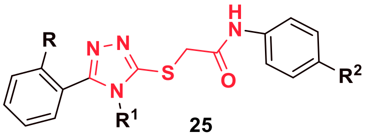

25

Compound	R	R^1	R^2	IC$_{50}$ (μM)
**25a**	H	2-OMe-phenyl	Br	0.0048
**25b**	H	2-OMe-phenyl	H	0.0238
**25c**	H	2-OMe-phenyl	F	0.0802
**25d**	H	2-OMe-phenyl	I	0.6545
**25e**	F	2-OMe-phenyl	I	0.0605
**25f**	F	2-OMe-phenyl	Br	0.1537
**25d**	F	2-OMe-phenyl	F	0.1691
**25h**	F	2-OMe-phenyl	H	0.5425
**25i**	H	*n*-Bu	H	0.2144
**25j**	H	*n*-Bu	I	0.2179
**25k**	H	*n*-Bu	Br	0.3431
**25l**	H	*n*-Bu	F	0.3696
kojic acid (**5**)				16.8320

Table S12. Triazoles derivatives, see **Figure 14** (Aryl phenoxy methyl triazoles conjugated with thiosemicarbazides derivatives).

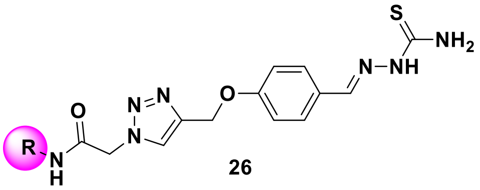

26

Compound	R	IC_{50} monophenolase (μM)	IC_{50} diphenolase (μM)
**26a**	benzyl	0.11	0.17
**26b**	4-OMe-phenyl	0.12	0.52
**26c**	4-F-phenyl	0.14	0.60
**26d**	3-OMe-phenyl	0.22	0.84
**26e**	2-Me,3-NO_2-phenyl	0.25	0.56
**26f**	4-Br-phenyl	0.35	0.21
**26g**	2,3-diCl-phenyl	0.42	0.42
**26h**	4-NO_2-phenyl	0.42	0.68
**26i**	2-Me,3-Cl-phenyl	0.46	0.26
**26j**	4-Cl-phenyl	0.50	0.14
**26k**	3-Br-phenyl	0.51	0.31
**26l**	3-Cl-phenyl	0.51	0.50
**26m**	phenyl	0.79	2.18
**26n**	4-F-benzyl	0.83	0.43
kojic acid (**5**)		9.28	9.30

Table S13. 1,2,4-Triazole-thiosemicarbazide derivatives, see **Figure 15** (1,2,4-Triazole-thiosemicarbazide hybrid molecules).

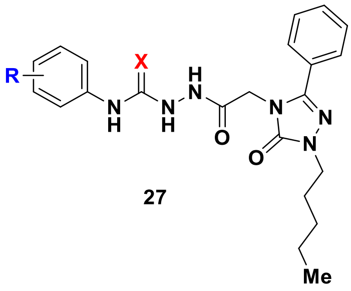

27

Compound	R	X	IC_{50} (μM)
**27a**	2,4-diCl	O	0.00162
**27b**	2-CF_3	O	0.00166
**27c**	3-CF_3	O	0.00165
**27d**	3,5-diCF_3	O	0.00197
**27e**	2-F	O	>20
**27f**	3-F	O	>20
**27g**	4-F	O	>20
**27h**	2,4-diF	O	>20
**27i**	2-Cl	O	>20
**27j**	3-Cl	O	>20
**27k**	4-Cl	O	>20
**27l**	3,5-diCl	O	>20
**27m**	2-Br	O	>20
**27n**	3-Br	O	>20

**27o**	4-Br	O	>20
**27p**	4-CF_3	O	>20
**27q**	4-F	S	0.0145
**27r**	2,4-diF	S	0.019
**27s**	4-Br	S	0.0199
**27t**	3,5-diCl	S	0.17
**27u**	4-Cl	S	0.1885
**27v**	4-CF_3	S	1.49
**27w**	2-Cl	S	4.68
**27x**	3-Cl	S	5.4
**27y**	2-CF_3	S	5.62
**27z**	3-F	S	15.2
**27aa**	2,4-diCl	S	>20
**27bb**	2-F	S	>20
**27cc**	2-Br	S	>20
**27dd**	3-Br	S	>20
**27ee**	3-CF_3	S	>20
**27ff**	3,5-diCF_3	S	>20
kojic acid (**5**)			14.09

Table S14. 1,2,4-Triazole based compounds, see **Figure 16** (1,2,4-Triazole based compounds (**28**) and their inhibitory activities).

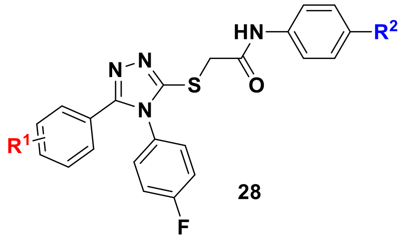

28

Compound	R^1	R^2	IC_{50} (μM)
**28a**	2-F	4-F	0.098
**28b**	2-F	H	0.111
**28c**	4-F	H	0.124
**28d**	H	4-Br	0.142
**28e**	H	H	0.219
**28f**	H	4-F	0.379
**28g**	4-F	4-F	-
**28h**	4-F	4-Br	-
**28i**	2-F	4-Br	-
kojic acid (**5**)			16.832

Table S15. Thiazole-triazole based compounds, see **Figure 17** (Thiazole-triazole hybrids as TYRIs).

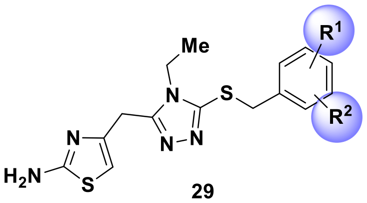

29

Compound	R^1	R^2	IC_{50} (μM)

**29a**	2-Cl	4-Cl	0.0018
**29b**	H	3-Br	0.0021
**29c**	2-Cl	H	0.0059
**29d**	H	4-Cl	0.0066
**29e**	H	4-F	0.0115
**29f**	3-Cl	4-Cl	0.0142
**29g**	2-Br	H	0.0157
**29h**	H	4-Br	0.0243
**29i**	H	3-Me	0.0341
**29j**	2-Me	H	0.0713
**29k**	H	4-Me	0.0782
**29l**	H	H	0.0896
kojic acid (**5**)			16.8320

Table S16. Tetrazole based compounds, see **Figure 18** (Tetrazole-containing TYRIs **30**).

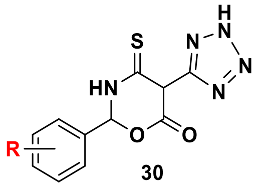

30

Compound	R	IC_{50} (μM)
**30a**	2-Br	0.0371
**30b**	2,4-diCl	0.0472
**30c**	3-NO_2	0.0601
**30d**	3-Cl	0.0715
**30e**	4-OMe	0.0728
**30f**	3,5-diNO_2	0.0758
**30g**	4-NO_2	0.0815
**30h**	2-Cl	0.0883
**30i**	2,6-diOMe	0.1992
**30j**	4-Cl	0.2941
**30k**	4-Me	0.3080
kojic acid (**5**)		16.832

Table S17. Chalcone derivatives, see **Figure 20** (TYRIs combining chalcone and furan moieties).

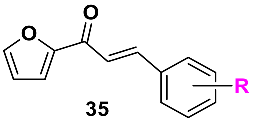

35

Compound	R	IC_{50} (μM)
**35a**	2,4-diOH	0.28
**35b**	3,4-diOH	24.86
**35c**	3,5-diBr,4-OH	27.17
**35d**	3-OH,4-OMe	37.36
**35e**	3-OMe,4-OH	>200
**35f**	3,5-diOMe,4-OH	>200
**35g**	3-Br,4-OH	>200
**35h**	4-OH	>200

kojic acid (**5**)		33.47

Table S18. Isobenzofuran-1(3*H*)-one derivatives, see **Figure 21** (Derivatives of isobenzofuran-1(3*H*)-one (**36a**) and their inhibitory profile).

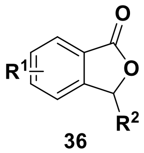

36

Compound	R^1	R^2	IC_{50} (µM)
**36a**	H	OH	156.03
**36b**	H	2,6-diCO_2Me-phenyl	194.01
**36c**	H	2,6-diOH,4-*i*Pr-phenyl	255.23
**36d**	5-OMe	H	-
**36e**	5-OH	H	-
**36f**	H	2,6-diOH,4-Me-phenyl	-
**36g**	H	2,6-diOH-phenyl	-
**36h**	H	2,6-diCO_2Me,4-Me-phenyl	-
**36i**	H	2,6-diCO_2Me,4-*i*Pr-phenyl	-
**36j**	6-OMe	H	-
**36k**	6-OH	H	-
kojic acid (**5**)			5.71

Table S19. Aurone derivatives, see **Figure 22**(Chemical structures of aurone derivatives **37** and their activities as AbTYRIs).

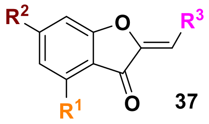

37

Compound	R^1	R^2	R^3	IC_{50} (µM)
**37a**	H	H	4-Me-phenyl	7.12
**37b**	H	H	3-OMe,4-OPr-phenyl	9.94
**37c**	H	H	5-OMe-2-naphtyl	10.12
**37d**	H	H	3-OMe,4-OBu-phenyl	10.56
**37e**	H	H	4-OMe-phenyl	10.65
**37f**	H	H	4-OEt-phenyl	12.52
**37g**	H	H	3-OMe,4-OHex-phenyl	13.35
**37h**	H	H	4-COOH-phenyl	14.23
**37i**	H	H	4-CHO-phenyl	15.24
**37k**	H	H	3-OMe,4-OBn-phenyl	20.82
**37l**	H	H	4-Cl-phenyl	34.31
**37m**	H	H	2-CF_3-phenyl	44.21
**37n**	H	H	2-thiophenyl	54.51
**37o**	H	H	4-F-phenyl	66.82
**37p**	Br	Br	4-Cl-phenyl	16.54
**37q**	Br	Br	4-CF_3-phenyl	19.76
**37r**	Br	Br	2-furanyl	39.45
**37s**	NO_2	Cl	3-OMe,4-OBn-phenyl	8.25
**37t**	NO_2	Cl	3-OMe,4-OPr-phenyl	9.55

**37u**	NO_2	Cl	2,4-diMe-phenyl	10.34
**37v**	NO_2	Cl	4-OMe-phenyl	13.10
**37w**	NO_2	Cl	4-Cl-phenyl	15.23
**37x**	NO_2	Cl	4-F-phenyl	16.33
**37y**	NO_2	Cl	4-N(CH_3)$_2$-phenyl	25.31
**37z**	NO_2	Cl	2-thiophenyl	26.90
kojic acid (**5**)				16.69

Table S20. Thiophene derivatives, see **Figure 23** (Thiophene derivatives).

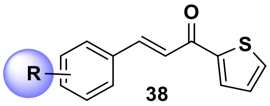

Compound	R	IC_{50} (μM)
**38a**	2,4-diOH	0.93
**38b**	3-OH,4-OMe	28.72
**38c**	4-OH	60.05
**38d**	3,4-diOH	103.44
**38e**	3-Br,4-OH	112.09
**38f**	3-OMe,4-OH	>200
**38g**	2,5-diBr,4-OH	>200
**38h**	3,5-diOMe,4-OH	>200
kojic acid (**5**)		0.93

Table S21. Isoxazole derivatives, see **Figure 24** ((*Z*)-4-(Substituted benzylidene)-3-phenylisoxazol-5(4*H*)-one derivatives).

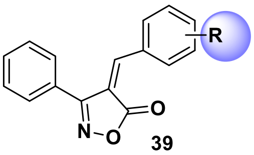

Compound	R	PI (%)	IC_{50} (μM)
**39a**	3-Br,4-OH	70.22	14.62
**39b**	2,4-diOH	72.73	-
**39c**	3,5-diOH,4-OH	47.29	-
**39d**	3-OEt,4-OH	44.52	-
**39e**	3-OH,4-OMe	42.03	-
**39f**	3,4-diOMe	40.02	
**39g**	4-OMe	28.45	-
**39h**	4-OH	24.60	-
**39i**	3-OMe,4-OH	-	-
**39j**	3,4-diOH	-	-
**39k**	3,4,5-triOMe	-	-
**39l**	2,4-diOH	-	-
**39m**	2-OH	-	-
kojic acid (**5**)		-	58.44

Table S22. 2-Thioxooxazoline-4-one derivatives, see **Figure 25** (2-Thioxooxazoline-4-one derivatives **44** as tyrosinase inhibitors).

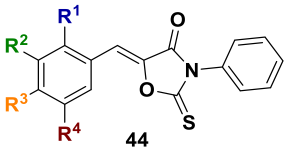

44

Compound	R[1]	R[2]	R[3]	R[4]	PI (%)	IC_{50} (μM)
**44a**	OH	H	OH	H	78.05	4.70
**44b**	OH	H	H	H	71.12	11.18
**44c**	H	OH	OH	H	30.57	-
**44d**	H	Br	OH	H	24.96	-
**44e**	H	OH	OMe	H	13.39	-
**44f**	H	H	OH	H	10.25	-
**44g**	H	OMe	OH	OMe	10.22	-
**44h**	H	*t*-Bu	OH	*t*-Bu	8.53	-
**44i**	H	OMe	OMe	H	7.47	-
**44j**	OMe	H	OMe	H	4.09	-
**44j**	H	Br	OH	Br	-	-
**44l**	F	H	F	H	-	-
**44m**	H	OMe	OH	H	-	-
**44n**	H	OEt	OH	H	-	-
**44o**	H	H	OMe	H	-	-
**44p**	H	OMe	OMe	OMe	-	-
kojic acid (**5**)					58.09	23.18

Table S23. 1,3,4-Oxadiazoles derivatives, see **Figure 26** (The tyrosinase inhibition potential of 1,3,4-oxadiazoles derivatives **45**).

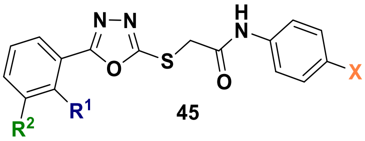

45

Compound	R[1]	R[2]	X	IC_{50} (μM)
**45a**	OMe	H	H	0.003
**45b**	OMe	H	F	0.018
**45c**	H	OMe	H	0.019
**45d**	OMe	H	I	0.023
**45e**	H	OMe	F	0.027
**45f**	H	H	Br	0.039
**45g**	OMe	H	Br	0.046
**45h**	H	H	I	0.085
**45i**	H	OMe	I	0.088
**45j**	H	OMe	Br	0.145
**45k**	H	H	H	0.231
**45l**	H	H	F	0.396
kojic acid (**5**)				16.832

Table S24. 2-Aminothiazole-oxadiazole derivatives, see **Figure 27** (2-Aminothiazole-oxadiazole bi-heterocyclic hybrids **46** and their potential as AbTYR inhibitors).

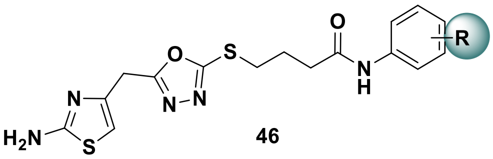

46

Compound	R	IC_{50} (µM)
**46a**	2-Me	0.0311
**46b**	2-Et	0.0349
**46c**	2,5-diMe	0.1987
**46d**	3,5-diMe	0.4611
**46e**	2,4-diMe	0.6524
**46f**	2,3-diMe	0.7379
**46g**	3-Me	1.0103
**46h**	2,6-diMe	1.6139
kojic acid (**5**)		16.8319

Table S25. Thiazolidinone derivatives, see **Figure 29** (Azo-hydrazone tautomeric dyes bearing a thiazolidinone moiety derivatives).

48

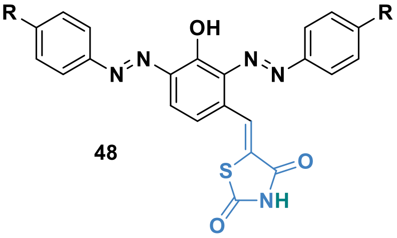

Compound	R	IC_{50} (µM)
**48a**	OMe	37.59
**48b**	NO_2	42.31
**48c**	Me	45.83
**48d**	CN	61.92
**48e**	Br	140.25
kojic acid (**5**)		29.44

Table S26. Thiazole derivatives, see **Figure 31a** (TYRIs containing hydrazinyl thiazole moieties).

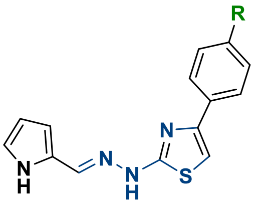

50

Compound	R	IC_{50} (µM)
**50a**	F	21.45
**50b**	Br	6.06
**50c**	OMe	5.94
**50d**	H	5.81
**50e**	Cl	4.95

kojic acid (**5**)		4.43

Table S27. Thiazole derivatives, see **Figure 31b** (TYRIs containing hydrazinyl thiazole moieties).

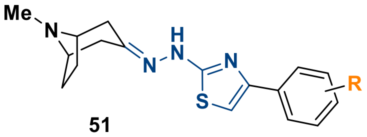

51

Compound	R	IC_{50} (μM)
**51a**	3,4-diCl-phenyl	3.22
**51b**	2,4-diCl-phenyl	3.51
**51c**	4-CF_3-phenyl	33.74
**51d**	N_3-phenyl	72.30
**51e**	4-Br-phenyl	84.97
**51f**	4-Me-phenyl	101.2
**51g**	4-NH_2-phenyl	118.06
**51h**	NHCOMe-phenyl	120.71
**51i**	4-F-phenyl	123.1
**51j**	4-Cl-phenyl	132.7
**51k**	4-NO_2-phenyl	140.21
**51l**	4-I-phenyl	151.44
**51m**	4-$NHSO_2Me$-phenyl	152.04
**51n**	4-CN-phenyl	153.04
**51o**	4-OMe-phenyl	165.38
**51p**	2*H*-chromen-2-one	183.34
Ascorbic acid (**7**)		386.5
kojic acid (**5**)		72.27

Table S28. 2-Benzothiazole-linked thiourea derivatives, see **Figure 32** (2-Benzothiazole-linked thioureas with inhibitory activity towards TYR).

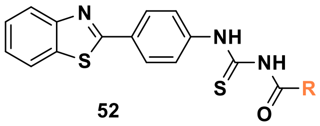

52

Compound	R	IC_{50} (μM)
**52a**	nonyl	1.3431
**52b**	heptyl	1.3526
**52c**	pentyl	2.6841
**52d**	octyl	4.1576
**52e**	3,5-diNO_2-phenyl	4.7745
**52f**	propyl	6.0986
**52g**	*t*-Bu	7.8319
**52h**	2,4-diCl-phenyl	19.0153
**52i**	4-OMe-phenyl	54.6311
kojic acid (**5**)		16.8320

Table S29. Benzylidenethiochroman-4-one derivatives, see **Figure 33** (Rational design of TYRIs bearing the benzylidenethiochroman-4-one scaffold).

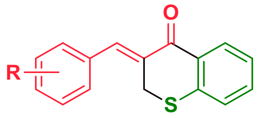

53

Compound	R	PI (%)	IC_{50} (µM)
**53a**	3-OH,4-OMe	91.5	4.1
**53b**	3-Br,4-OH	90.7	-
**53c**	3,4-diOH	45.0	-
**53d**	3-OMe,4-OH	25.8	-
**53e**	2,4-diOMe	20.3	-
**53f**	3,5-diOMe,4-OH	15.6	-
**53g**	3,5-diBr,4-OH	8.5	-
kojic acid (**5**)		77.5	22.0

Table S30. ((*Z*)-5-(Substituted benzylidene)-4-thioxothiazolidin-2-one derivatives, see **Figure 34** ((*Z*)-5-(Substituted benzylidene)-4-thioxothiazolidin-2-one derivatives (**56**) as tyrosinase inhibitors).

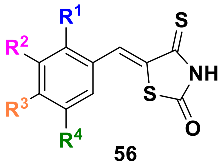

56

Compound	R^1	R^2	R^3	R^4	IC_{50} (µM)
**56a**	OH	H	OH	H	0.47
**56b**	H	OH	OMe	H	15.24
**56c**	H	OMe	OMe	H	23.31
**56d**	H	Br	OH	H	26.27
**56e**	H	H	OH	H	28.05
**56f**	H	H	OMe	H	30.83
**56g**	H	OH	OH	H	47.41
**56h**	H	OMe	OH	H	70.75
**56i**	H	OMe	OH	OMe	82.39
**56l**	H	OEt	OH	H	92.81
**56m**	OMe	H	OMe	H	126.35
**56n**	OH	H	H	H	147.61
kojic acid (**5**)					66.30

Table S31. (*Z*)-5-(Substituted benzylidene)-3-phenyl-2-thioxothiazolidin-4-one derivatives, see **Figure 35** (*Z*)-5-(Substituted benzylidene)-3-phenyl-2-thioxothiazolidin-4-one derivatives **57**, that showed promising inhibitory characteristics).

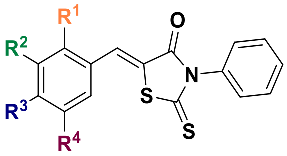

57

Compound	R^1	R^2	R^3	R^4	IC_{50} (µM)

**57a**	OH	H	OH	H	0.59
**57b**	H	OH	OH	H	1.38
**57c**	H	H	OH	H	1.45
**57d**	H	OH	OMe	H	1.88
**57e**	OMe	H	OMe	H	22.15
**57f**	H	Br	OH	H	65.54
**57g**	H	Br	OH	Br	78.75
**57h**	H	OMe	OH	H	80.03
**57i**	H	OMe	OMe	H	94.29
**57j**	H	H	OMe	H	>200
**57k**	H	OMe	OMe	OMe	>200
**57l**	H	OMe	OH	OMe	>200
**57m**	H	OEt	OH	H	>200
kojic acid (**5**)					17.05

Table S32. 2-(Aryl)-5-(trifluoromethyl)benzo[*d*]thiazoles, see **Figure 36** (Inhibitory activities towards TYR of 2-(aryl)-5-(trifluoromethyl)benzo[*d*]thiazoles **58**).

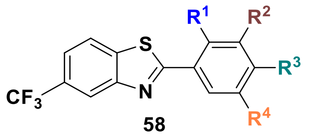

58

Compound	R^1	R^2	R^3	R^4	IC_{50} (μM)
**58a**	OH	H	OH	H	0.2
**58b**	H	H	OH	H	52.4
**58c**	H	Br	OH	H	101.9
**58d**	H	Me	OH	Me	128.9
**58e**	H	OH	H	OH	216.5
**58f**	H	OMe	OMe	H	263.9
**58g**	H	OMe	OH	H	291.1
**58h**	H	OH	OMe	H	>300
**58i**	OH	H	H	H	>300
**58j**	H	H	OMe	H	>300
**58k**	OMe	H	OMe	H	>300
**58l**	H	Br	OH	Br	>300
**58m**	H	OEt	OH	H	>300
**58n**	H	OMe	OMe	OMe	>300
**58o**	H	OMe	OH	OMe	>300
**58p**	H	OH	OH	H	>300
kojic acid (**5**)					12.6

Table S33. ((*Z*)-2-Benzylidene-dihydroimidazothiazolone derivatives, see **Figure 37a** ((*Z*)-2-Benzylidene-dihydroimidazothiazolone derivatives (**59**) (a)).

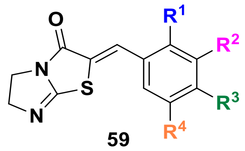

59

Compound	R^1	R^2	R^3	R^4	IC_{50} (μM)
**59a**	OH	H	OH	H	0.88

**59b**	H	OH	OMe	H	17.10
**59c**	H	H	OH	H	36.14
**59d**	H	OH	OH	H	>100
**59e**	H	OMe	OH	H	>100
**59f**	H	OEt	OH	H	>100
**59g**	H	H	OMe	H	>100
**59h**	OMe	H	OMe	H	>100
**59i**	H	OMe	OMe	H	>100
**59j**	H	OMe	OH	OMe	>100
**59k**	H	OMe	OMe	OMe	>100
kojic acid (**5**)					84.41

Table S34. ((*Z*)-2-(Benzylamino)-5-benzylidenethiazol-4(5*H*)-one derivatives, see **Figure 37b** ((*Z*)-2-(Benzylamino)-5-benzylidenethiazol-4(5*H*)-one derivatives (**60**) (b)).

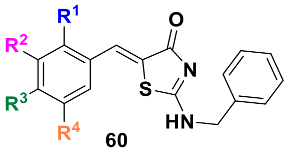

60

Compound	R^1	R^2	R^3	R^4	IC_{50} (μM)	
**60a**	OH	H	OH	H	0.27	1.04
**60b**	H	OH	OMe	H	10.0	10.3
**60c**	H	H	OH	H	27.5	65.1
**60d**	OMe	H	OMe	H	293.3	213.6
**60e**	H	OMe	OMe	OMe	192.4	231.1
**60f**	H	H	OMe	H	125.5	239.4
**60g**	H	OEt	OH	H	198.9	248.1
**60h**	F	H	F	H	>300	>300
**60i**	H	F	F	H	288.7	>300
**60j**	H	OMe	OH	H	148.3	>300
**60k**	H	OMe	OMe	H	>300	>300
**60l**	H	OMe	OH	OMe	166.0	>300
**60m**	H	t-Bu	OH	t-Bu	136.7	>300
**60n**	H	OH	OH	H	>300	>300
kojic acid (**5**)					28.6	20.1

Table S35. Hydroxypyridinone derivatives, see **Figure 39** (Hydroxypyridinone-based TYRIs (**63**) bearing an oxime moiety).

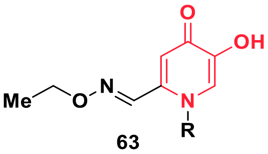

63

Compound	R	IC_{50} monophenolase (μM)	IC_{50} diphenolase (μM)
**63a**	*n*-Octyl	1.60	7.99
**63b**	*n*-Hexyl	2.04	13.89
**63c**	*n*-Bu	4.78	-
**63d**	Et	7.45	-
**63e**	Me	19.65	-

**63f**	H	22.87	-
kojic acid (**5**)		12.24	-

Table S36. Hydroxypyridinones, see **Figure 40** (TYRIs bearing a chalcone-hydroxypyridinone scaffold)

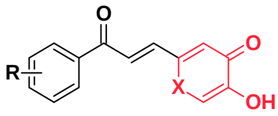

64

Compound	R	X	PI (%)	IC_{50} monophenolase (μM)	IC_{50} diphenolase (μM)
**64a**	4-F	NH	89.5	2.25	11.7
**64b**	3,4-diOCH_3	NH	87.6	2.75	19.3
**64c**	4-F	NCH_3	85.3	3.07	17.1
**64d**	3,4-diOH	NC_2H_5	78.8	8.11	-
**64e**	H	NCH_3	72.8	-	-
**64f**	2-OH, 4-OCH_3	NC_2H_5	71.4	-	-
**64g**	3,4-diOH	NC_4H_9	69.4	-	-
**64h**	2-OH, 4-OCH_3	NCH_3	66.7	-	-
**64i**	H	NH	66.7	-	-
**64j**	2-OH, 4-OCH_3	NC_4H_9	64.6	-	-
**64k**	H	NC_2H_5	63.3	-	-
**64l**	3,4-diOCH_3	NH	63.3	-	-
**64m**	4-F	NC_2H_5	57.8	-	-
**64n**	2,4-diOCH_3	O	50.3	-	-
**64o**	4-F	NC_4H_9	50.3	-	-
kojic acid (**5**)			75.4	-	-

Table S37. Hydroxypyridinones, see **Figure 41** (TYR-inhibitory profile of stilbene-hydroxypyridinone hybrids).

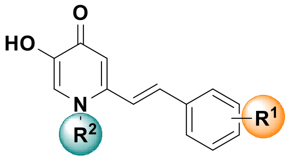

65

Compound	R^1	R^2	IC_{50} monophenolase (μM)	IC_{50} diphenolase (μM)
**65a**	3,5-diOH	n-C_6H_{13}	2.72	15.86
**65b**	3,4-di-OH	n-C_6H_{13}	3.83	-
**65c**	4-OH	n-C_6H_{13}	5.27	-
**65d**	3,5-diOH	n-C_4H_9	6.35	-
**65e**	3,4-diOH	n-C_4H_9	7.45	-
**65f**	4-OH	n-C_4H_9	7.62	-
**65g**	3,5-diOH	n-C_8H_{17}	9.31	-
**65h**	3,4-diOH	n-C_8H_{17}	12.26	-
**65i**	4-OH	n-C_8H_{17}	16.32	-
kojic acid (**5**)			12.52	-

Table S38. Quinoline derivatives, see **Figure 42** (Dihydroquinolinones and their TYR inhibitory behavior).

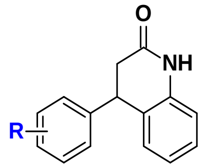

66

Compound	R	PI (%)
**66a**	3-OH,4-OMe	25.94
**66b**	4-OH	-
**66c**	3,4-diOH	-
**66d**	3-OMe,4-OH	-
kojic acid (**5**)		21.39

Table S39. Quinoline derivatives, see **Figure 44** (Quinolinyl based acyl thiourea derivatives).

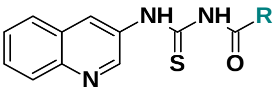

67

Compound	R	IC_{50} (µM)
**67a**	*n*-pentyl	0.0070
**67b**	phenyl	0.0148
**67c**	3,5-diNO_2-Ph	0.1099
**67d**	*n*-hexyl	0.1105
**67e**	*n*-heptyl	0.1149
**67f**	4-Me-Ph	0.1275
**67g**	*n*-nonyl	0.2685
**67h**	*n*-butyl	0.4196
**67i**	2,4-diCl-Ph	0.7656
**67j**	*n*-propyl	1.9737
kojic acid (**5**)		16.8320

Table S40. Pyrimidine-based derivatives, see **Figure 44** (Azo-pyrimidine derivatives that were tested on their inhibitory activity on TYR).

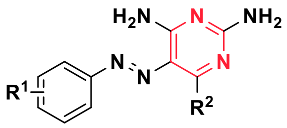

68

Compound	R^1	R^2	IC_{50} (µM)
**68a**	4-NHCOMe	Cl	24.45
**68b**	2-NO_2	Cl	24.68
**68c**	4-OMe	Cl	28.01
**68d**	4-NHCOMe	NH_2	29.77
**68e**	2-NO_2	NH_2	34.06
**68f**	4-NO_2	Cl	37.191
**68g**	4-NO_2	NH_2	40.62
**68h**	4-Me	NH_2	41.4

**68i**	4-Me	Cl	42.04
**68j**	4-OMe	NH_2	43.8
kojic acid (**5**)			25.24

Table S41. Pyrimidine-based derivatives, see **Figure 45** (TYRIs bearing the pyranopyrimidine and the pyranotriazolopyrimidine scaffolds).

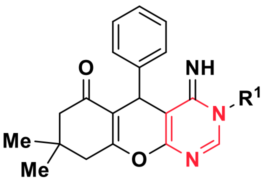

69

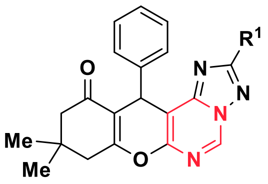

70

Compound	R^1	PI (%)
**69a**	3-Cl-Ph	94.23
**69b**	Ph	91.35
**69c**	4-C_2H_5-Ph	84.14
**69d**	4-Me-Ph	-
**69e**	1-naphtyl	-
**69f**	4-OMe-Ph	-
**70a**	CH_2CN	93.94
**70b**	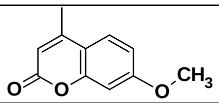	90.12
**70c**	H	78.09
**70d**	Me	-
kojic acid (**5**)		85.50

Table S42. Pyrimidine-based derivatives, see **Figure 47** (Tetrahydrobenzo[4,5]thieno[2,3-d]pyrimidine-4(3*H*)-one derivatives **72**, which are substituted in the 2-position).

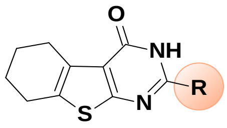

72

Compound	R	IC_{50} (mM)
**72a**	2,4-diOH-Ph	0.51
**72b**	4-OH-Ph	0.70
**72c**	2-OH-Ph	0.87
**72d**	Ph	1.11
**72e**	2-OH,3-OMe-Ph4	1.57
**72f**	3-OMe,4-OH	2.04
**72g**	3-Me-furan	2.10
**72h**	*t*Bu-Ph	2.11
**72i**	4-Me-Ph	2.25
**72j**	4-OMe-Ph	2.34
**72k**	4-OMe,3-OH	2.45
**72l**	furan	3.03
kojic acid (**5**)		0.32

Table S43. Oxoquinazoline derivatives, see **Figure 48** (Oxoquinazoline derivatives **73** and their potential in inhibition of TYR).

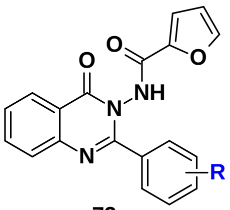

73

Compound	R	IC_{50} (µM)
**73a**	2-OH,3,5-diBr	0.028
**73b**	2-OH,3-NO_2	0.031
**73c**	2-OH,5-Cl	0.052
**73d**	2-OH,3,5diI	0.055
**73e**	2,4-diOH	0.056
**73f**	2-OH,5-br	0.063
**73g**	2-OH	0.499
**73h**	2-OH,3,5-diCl	0.627
**73i**	2-OH,4-$(CH_3CH_2)_2NH$	1.218
**73j**	2-OH,5-OMe	1.775
kojic acid (**5**)		16.832

Table S44. Piperazines, see **Figure 50** (Further 4-fluorobenzylpiperazine derivatives (**78**) which were identified as TYRIs).

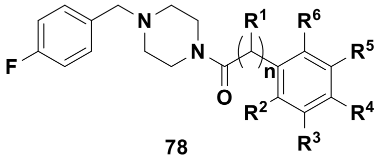

78

Compound	*n*	R^1	R^2	R^3	R^4	R^5	R^6	IC_{50} (µM)
**78a**	0	H	NO_2	Cl	H	H	H	0.18
**78b**	0	H	CF_3	H	H	F	H	0.24
**78c**	0	H	CF_3	F	H	H	H	0.45
**78d**	0	H	CF_3	H	F	H	H	0.87
**78e**	0	H	NO_2	H	H	OMe	H	1.11
**78f**	0	H	NO_2	H	H	H	Cl	1.17
**78g**	1	H	H	H	NO_2	H	H	1.71
**78h**	0	H	NO_2	H	OMe	H	H	1.80
**78i**	0	H	NO_2	H	H	Cl	H	2.01
**78j**	0	H	NO_2	OMe	H	H	H	2.09
**78k**	0	H	NO_2	H	Cl	H	H	2.27
**78l**	1	H	H	H	NO_2	H	H	3.74
**78m**	1	H	H	H	H	H	H	4.28
**78n**	1	H	H	Cl	H	H	H	4.66
**78o**	1	H	Br	H	H	H	H	4.70
**78p**	1	H	H	H	Cl	H	H	4.71
**78q**	1	H	H	H	Br	H	H	7.66
**78r**	1	H	NO_2	H	H	H	H	8.31
**78s**	1	H	H	NO_2	H	H	H	8.66

**78t**	1	H	Cl	H	H	H	H	8.70
**78u**	1	H	H	NH_2	H	H	H	9.36
**78v**	2	H	H	H	H	H	H	13.17
**78w**	1	H	H	Br	H	H	H	15.74
**78x**	1	H	H	H	NH_2	H	H	16.73
**78y**	1	H	H	H	OMe	H	H	19.63
**78z**	1	Ph	H	H	H	H	H	40.43
kojic acid (**5**)								17.76

Table S45. Piperazines, see **Figure 51a** (Phenylpiperazine TYRIs (**79** - **81**) from various studies).

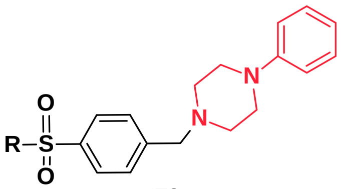

79

Compound	R	IC_{50} (μM)
**79a**	1-piperidinyl	0.06
**79b**	3,5-dimethyl-1-piperidinyl	0.07
**79c**	4-methyl-1-piperidinyl	0.14
**79d**	4-morpholinyl	0.41
kojic acid (**5**)		16.83

Table S46. Piperazines, see **Figure 51b** (Phenylpiperazine TYRIs (**79** - **81**) from various studies).

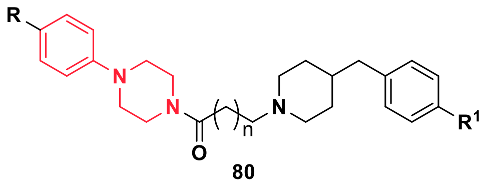

80

Compound	*n*	R	R^1	IC_{50} (μM)
**80a**	0	OH	H	3.80
**80b**	1	OH	H	4.03
**80c**	0	OH	F	4.49
**80d**	1	OH	F	6.06
**80e**	0	H	F	9.60
**80f**	1	F	F	23.36
**80g**	1	H	H	57.50
**80h**	0	H	H	80.86
kojic acid (**5**)				17.76

Table S47. Piperazines, see **Figure 51c** (Phenylpiperazine TYRIs (**79** - **81**) from various studies).

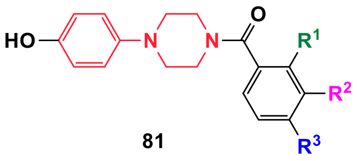

Compound	R^1	R^2	R^3	IC_{50} (μM)
**81a**	Cl	H	Cl	1.5
**81b**	Cl	H	H	2.6
**81c**	OMe	H	H	3.5
**81d**	Br	H	H	4.5
**81e**	CF_3	H	H	4.6
**81f**	H	CF_3	H	5.5
**81g**	NO_2	H	H	7.4
**81h**	NO_2	H	NO_2	7.6
**81i**	H	H	Cl	8.9
**81l**	H	Cl	H	9.0
**81j**	Br	H	Br	9.5
**81m**	H	OMe	H	9.8
**81n**	H	H	OMe	14.9
**81o**	F	H	H	15.2
**81p**	H	Br	H	16.4
**81q**	OMe	H	OMe	16.7
**81r**	F	H	F	17.5
**81s**	CF_3	H	CF_3	18.2
**81t**	H	H	F	21.7
**81u**	H	NO_2	H	21.8
**81v**	H	F	H	22.6
**81w**	H	H	NO_2	23.2
**81x**	Me	H	H	29.9
**81y**	H	Me	H	31.7
**81z**	Me	H	Me	33.7
**81aa**	H	H	Me	34.4
**81bb**	H	H	CF_3	35.1
**81cc**	H	NH_2	H	35.7
**81dd**	H	H	Br	39.6
**81ee**	H	H	NH_2	43.9
**81ff**	NH_2	H	H	66.4
**81gg**	H	H	H	73.2
**81hh**	NH_2	H	NH_2	82.4
**81ii**	H	H	Ph	128.3
kojic acid (**5**)				17.76

Table S48. Piperazines, see **Figure 52** (Piperazinyl butanamide-containing TYRIs).

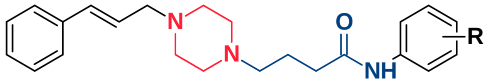

82

Compound	R	IC_{50} (μM)
**79a**	2,4-diMe	0.01
**79b**	2,3-diMe	0.02
**79c**	3-Me	0.04
**79d**	4-Me	0.05
**79e**	4-Et	0.68
kojic acid (**5**)		16.84

Table S49. Piperazines, see **Figure 53** (Cinnamic acid derivatives linked to aryl piperazines).

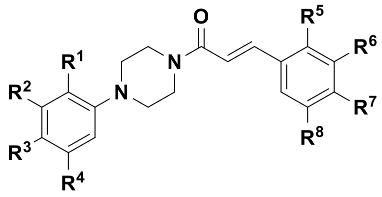

83

Compound	R^1	R^2	R^3	R^4	R^5	R^6	R^7	R^8	IC_{50} (μM)
**83a**	H	Cl	F	H	H	NO_2	H	H	0.16
**83b**	H	Cl	F	H	Cl	OMe	H	H	0.12
**83c**	H	Cl	F	H	NO_2	OMe	H	H	0.51
**83d**	H	Cl	F	H	H	OMe	H	H	0.25
**83e**	H	Cl	F	H	H	H	Cl	H	0.33
**83f**	H	Cl	F	H	H	H	NO_2	H	0.35
**83g**	H	Cl	F	H	OMe	H	H	Cl	0.46
**83h**	H	Cl	F	H	H	NH_2	OMe	H	0.53
**83i**	H	F	F	H	H	NH_2	OMe	H	6.84
**83j**	H	H	F	H	H	OMe	H	H	9.96
**83k**	H	H	F	H	H	H	Cl	H	14.09
**83l**	H	F	F	H	H	NH_2	OMe	H	15.47
**83m**	H	H	F	H	H	NH_2	OMe	H	17.85
**83n**	H	H	F	H	H	NO_2	H	H	23.32
**83o**	OMe	H	H	H	H	H	H	Cl	27.40
**83p**	H	Cl	H	H	H	OCH_2O	OCH_2O	H	34.25
**83q**	H	F	H	H	H	NH_2	OMe	H	>100
**83r**	H	H	F	H	H	H	NO_2	H	NI
**83s**	H	H	F	H	H	OCH_2O	OCH_2O	H	NI
**83t**	H	H	F	H	H	OBn	NO_2	H	NI
**83u**	H	H	F	H	H	NO_2	OMe	H	NI
**83v**	H	F	H	H	H	NO_2	OMe	H	NI
**83w**	H	F	H	F	H	NO_2	OMe	H	NI
**83x**	F	H	Cl	H	H	NH_2	OMe	H	NI
**83y**	H	OMe	H	H	H	OBn	H	H	NI
**83z**	Cl	H	H	H	OMe	H	H	Cl	NI
kojic acid (**5**)									17.76

Table S50. Quinazolines, see **Figure 54** (Chlorophenylquinazolin-4(3*H*)-one derivatives **84** with inhibitory potential of TYR).

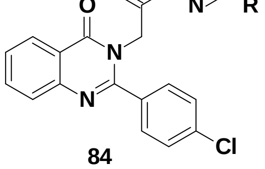

84

Compound	R	PI (%)	IC_{50} (μM)
**84a**	Ph-O-Ph	63.68	25.48
**84b**	4-Me-Ph	53.84	-
**84c**	2-NO_2,3-OMe-Ph	49.77	-

**84d**	3-OMe-Ph	41.78	-
**84e**	2-F-Ph	38.58	-
**84f**	3-NO_2-Ph	29.28	-
**84g**	4-OMe-Ph	29.22	-
**84h**	2-Me-Ph	25.64	-
**84i**	2-naphtyl	24.61	-
**84j**	4-NO_2-Ph	22.60	-
**84k**	2-Cl-Ph	22.37	-
**84l**	2-thienyl	17.17	-
**84m**	Ph	7.30	-
**84n**	2-Cl,5-NO_2	1.02	-
kojic acid (**5**)			9.30

Table S51. Quinazolines, see **Figure 55** (Quinazoline derivatives **85** and **86** in three-digit micromolar range of TYR inhibition).

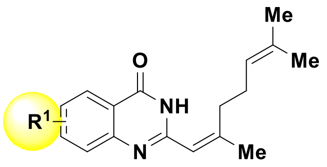

85

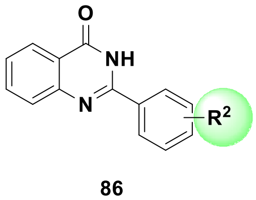

86

Compound	R^1	R^2	IC_{50} (µM)
**85a**	H	-	103
**85b**	5-F	-	105
**85c**	6-Cl	-	168
**85d**	7-Me	-	253
**86a**	-	H	-
**86b**	-	4-Me	-
**86c**	-	4-OMe	-
arbutin (**6**)			180

Table S52. Miscellaneous, see **Figure 56** (Cinnamide derivatives and their percentage of inhibition of monophenolase).

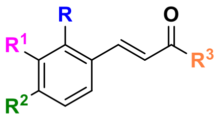

87

Compound	R	R^1	R^2	R^3	PI (%)
**87a**	OH	H	OH	pyrrolidine	93.72
**87b**	OH	H	OH	piperidine	78.97
**87c**	OH	H	OH	1-methyl-piperazine	94.39
**87d**	OH	H	OH	morpholine	95.74
**87e**	H	OMe	OH	piperidine	59.09
**87f**	H	OH	OMe	1-methyl-piperazine	40.22
**87f**	H	OH	OMe	piperidine	36.70
**87g**	H	OH	OMe	morpholine	31.12
**87h**	H	OH	OMe	pyrrolidine	26.42

**87i**	H	OMe	OH	pyrrolidine	24.00
**87i**	H	OMe	OH	morpholine	21.28
**87j**	H	OMe	OH	1-methyl-piperazine	19.33
**87k**	H	H	OH	piperidine	19.07
**87l**	H	H	OH	pyrrolidine	13.65
**87m**	H	OH	OH	piperidine	12.43
**87n**	H	OH	OH	pyrrolidine	3.41
**87o**	H	H	OH	1-methyl-piperazine	-
**87p**	H	OH	OH	1-methyl-piperazine	-
**87q**	H	H	OH	morpholine	-
**87r**	H	OH	OH	morpholine	-
kojic acid (**5**)					20.57

Table S53. Miscellaneous, see **Figure 57** (Morpholine containing cinnamides and inhibition of TYR thereof).

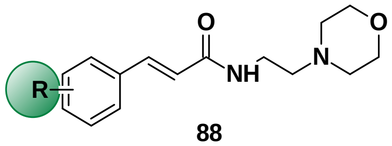

88

Compound	R	IC_{50} (µM)
**88a**	3-Cl	15.2
**88b**	3-Me	21.3
**88c**	4-NO_2	25.2
**88d**	4-Cl	28.6
**88e**	3-NO_2	35.5
**88f**	2-NO_2	36.0
**88g**	4-F	40.6
**88h**	3,4,5-OMe	50.0
**88i**	H	75.8
**88j**	2F	>100
**88k**	3-F	>100
**88l**	2-Cl	>100
**88m**	2-OMe	>100
**88n**	3-OMe	>100
**88o**	4-OMe	>100
**88p**	3,4-diOMe	>100
**88q**	2-Me	>100
**88r**	4-Me	>100
**88s**	4-OH,3-OMe	>100
kojic acid (**5**)		14.4

Table S54. Chromone derivatives, see **Figure 59** (Chromone derivatives and their inhibition of TYR).

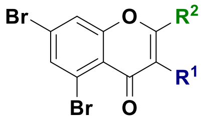

90

Compound	R^1	R^2	IC_{50} (µM)
**90a**	OH	3,4-diOMe-Ph	0.093

**90b**	OH	3-NO_2-Ph	0.15
**90c**	H	indole	0.22
**90d**	OH	4-Me-Ph	0.126
**90e**	OH	4-NO_2-Ph	0.27
**90f**	H	4-NO_2-Ph	0.27
**90g**	H	3,4-diOMe-Ph	0.49
**90h**	OH	4-N$(Me)_2$-Ph	0.50
**90i**	OH	1-naphtyl	0.564
**90j**	H	4-N$(Me)_2$-Ph	0.57
**90k**	H	2-thienyl	0.68
**90l**	OH	4-Cl-Ph	0.71
**90m**	H	2-furyl	2.07
**90n**	OH	4-F-Ph	2.36
**90o**	OH	2-furyl	2.5
**90p**	OH	2-thienyl	3.01
**90q**	H	3-NO_2-Ph	3.28
**90r**	H	1-naphtyl	3.41
**90s**	H	4-Cl-Ph	4.2
**90t**	OH	4-CO_2H-Ph	4.26
**90u**	H	4-Me-Ph	7.2
**90v**	7	2,4-diMe-Ph	7.57
**90w**	H	4-CO_2H-Ph	9.7
**90x**	H	2,4-diOMe-Ph	11.33
**90y**	H	4-F-Ph	20.5
**90z**	OH	indole	23.58
kojic acid (**5**)			1.79

Table S55. Chromone derivatives, see **Figure 60** (3-Hydroxyflavone derivatives **91** as TYRIs).

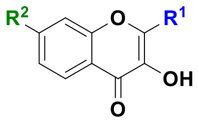

91

Compound	R^1	R^2	IC_{50} (µM)
**91a**	4-Cl-Ph	OH	0.23
**91b**	4-NO_2-Ph	OH	0.28
**91c**	2-thienyl	OH	0.35
**91d**	4-NO_2-Ph	H	0.284
**91e**	N$(Ph)_3$	H	0.327
**91f**	2-thienyl	H	0.347
**91g**	4-F-Ph	H	0.799
**91h**	4-OMe-Ph	H	1.787
**91i**	4-OMe-Ph	Br	2.236
**91j**	3-NO2-Ph	H	2.564
**91k**	1-naphtyl	H	2.90
**91l**	Ph	H	4.268
**91m**	4-Br-Ph	H	4.437
**91n**	4-*i*Bu-Ph	H	4.844
**91o**	3-Br-Ph	Cl	5.783
**91p**	4-Br-ph	Br	7.226

**91q**	4-Me	H	14.171
**91r**	4-*i*Bu-Ph	Br	19.595
**91s**	4-CO_2H-Ph	H	35.189
kojic acid (**5**)			1.79

Table S56. Chromone derivatives, see **Figure 61** (Thioflavones and thioflavonols derivatives and their activities on TYR inhibition).

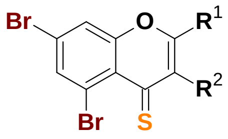

92

Compound	R^1	R^2	IC_{50} (µM)
**92a**	3-NO_2-Ph	OH	1.12
**92b**	4-Cl-Ph	OH	1.16
**92c**	4-N$(Me)_2$-Ph	OH	1.37
**92d**	4-NO_2-Ph	OH	1.45
**92e**	3,4-diOMe-Ph	OH	1.49
**92f**	2-naphtyl	OH	1.55
**92g**	2,4-diMe-Ph	OH	1.70
**92h**	4-Cl-Ph	H	3.46
**92i**	2-naphtyl	H	3.90
**92j**	2,4-diMe-Ph	H	3.96
**92k**	4-Me-Ph	OH	4.60
**92l**	3-NO_2-Ph	H	4.94
**92m**	2-thienyl	H	5.06
**92n**	3,4-diOMe-Ph	H	5.68
kojic acid (**5**)			12.6

Table S57. Xanthone derivatives, see **Figure 62** (1-hydroxyl xanthone (**93**), and derived 3-aryl substituted xanthone derivatives and their TYR inhibitory potencies).

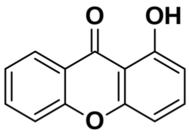

93

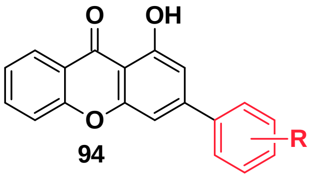

94

Compound	R	IC_{50} (µM)
**93**	-	>150
**94a**	4-OH	11.3
**94b**	2-OH	14.1
**94c**	3-OH	16.2
**94d**	3,4-diOMe	34.8
**94e**	3,4,5-*tri*OMe	35.2
**94f**	4-OMe	36.5
**94g**	2,4-diOMe	37.4
**94h**	3,5-diOMe	39.8
**94i**	4-CHO	42.1

**94j**	4-CO_2H	44.2
**94k**	4-COMe	45.1
**94l**	2-CHO	45.6
**94m**	3,4-diOH	50.7
**94n**	3,4,5-*tri*OH	52.6
**94o**	2,4-diOH	53.6
**94p**	3-CHO	54.1
**94q**	2-CO_2H	55.1
**94r**	4-COOMe	56.1
**94s**	2-CO_2H	73.9
**94t**	2-COOMe	78.1
**94u**	2-OMe	82.2
**94v**	3,5-diOH	92.6
**94w**	3-OMe	109.1
**94x**	3-COOMe	115.8
kojic acid (**5**)		17.3

Table S58. Xanthone derivatives, see **Figure 64** (Further xanthone derivatives with promising inhibitory activity of TYR).

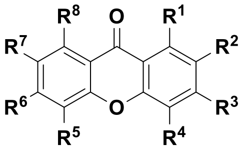

98

Compound	R^1	R^2	R^3	R^4	R^5	R^6	R^7	R^8	IC_{50} (μM)
**98a**	Me	Cl	OMe	OMe	H	OMe	H	H	1.9
**98b**	Me	H	OMe	OMe	H	H	H	H	2.0
**98c**	Me	H	OMe	OMe	H	OMe	H	H	2.24
**98d**	OH	OMe	OH	H	H	H	H	H	3.01
**98e**	CHO	H	OMe	OMe	H	OMe	H	H	3.16
**98f**	Me	H	OH	OH	H	OH	H	H	3.28
**98g**	CH_2Br	H	OMe	OMe	H	H	H	H	3.37
**98h**	H	H	OH	OMe	H	H	H	H	3.81
**98i**	OMe	OMe	H	H	H	H	H	H	4.02
**98j**	H	H	OH	H	H	OH	H	H	4.42
**98k**	OH	H	H	H	H	H	OH	H	4.52
**98l**	H	H	OMe	OMe	H	H	H	H	5.12
**98m**	H	H	OH	OH	H	H	H	H	5.12
**98n**	H	H	OMe	H	H	H	H	H	5.14
**98o**	OMe	H	H	H	H	H	H	H	5.14
**98p**	H	H	OMe	OH	H	H	H	H	5.2
**98q**	H	H	H	OMe	H	H	H	H	5.33
**98r**	Me	Br	OMe	OMe	H	H	H	H	5.57
**98s**	H	OH	H	H	H	H	H	H	5.60
**98t**	H	OH	OH	H	H	H	H	H	5.70
**98u**	OH	OH	H	H	H	H	H	H	7.8
**98v**	H	OMe	OMe	H	H	H	H	H	8.29
**98w**	H	H	H	OH	H	H	H	H	8.46

**98x**	H	OMe	H	H	H	H	H	H	8.47
**98y**	OH	H	H	H	H	H	H	H	8.83
**98z**	H	H	H	H	H	H	H	H	8.84
**98aa**	Me	H	OH	OH	H	H	H	H	8.93
**98bb**	H	H	OH	H	H	H	H	H	9.26
**98cc**	CHO	H	OMe	OMe	H	H	H	H	29.13
**98dd**	CHO	H	OH	OH	H	H	H	H	89.37
kojic acid (**5**)									12.81

Table S59. Coumarin derivatives, see **Figure 65** (Coumarin derivatives **99** and **100** as TYRIs).

Hydroxycoumarin derivatives

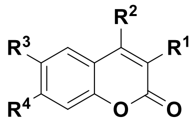

99

Geranyloxycoumarin derivatives

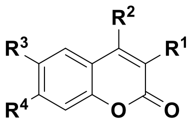

100

Compound	R^1	R^2	R^3	R^4	PI (%)	IC_{50} (μM)
**99a**	H	H	OH	H	191	-
**99b**	CN	H	H	OH	158	-
**99c**	H	Me	OH	H	102	-
**99d**	H	Me	Cl	OH	101	-
**99e**	OH	H	H	H	81	-
**99f**	Cl	Me	H	OH	79	-
**99g**	NO_2	OH	H	H	68	-
**99h**	H	Me	OH	OH	35	-
**99i**	H	H	H	OH	15	-
**99j**	H	CH_2CO_2H	H	OH	14	-
**100a**	H	Me	geranyloxy	geranyloxy	200	0.67
**100b**	H	Me	Cl	geranyloxy	137	-
**100c**	H	H	H	geranyloxy	132	-
**100d**	H	CF_3	H	geranyloxy	127	-
**100e**	H	Me	H	geranyloxy	121	-
**100f**	geranyloxy	H	H	H	107	-
**100g**	H	Me	H	geranyloxy	97	-
**100h**	Ph	H	H	geranyloxy	79	-
arbutin (**6**)					100	-

Table S60. Coumarin derivatives, see **Figure 66a** (Coumarin derivatives with either aryl groups, **101** and **102**).

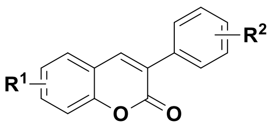

101

or

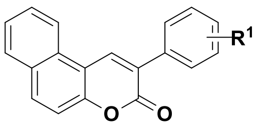

102

Compound	R_1	R_2	IC_{50} (μM)
**101a**	2,4-diOH	4-Br	1.05
**101b**	5,7-diOH	3-Br	7.0
**101c**	6,7-diOH	3-Br	8.3

**101d**	6,7-diOH	4-Br	39.9
**101e**	7-OH	3-Br	42.0
**101f**	5,7-diOH	2-Br	>50
**101g**	6,7-diOH	2-Br	>50
**101h**	7,8-diOH	4-Br	>50
**101i**	7,8-diOH	3-Br	>50
**101j**	7,8-diOH	2-Br	>50
**101k**	6,7-diOH	3-OH	>50
**101l**	6,7-diOH	2-OH	>50
**102a**	4-OH	-	>50
**102b**	3-OH	-	>50
**102c**	2-OH	-	>50
**102d**	3,4-diOH	-	>50
**102e**	3,5-diOH	-	>50
kojic acid (**5**)			17.9

Table S61. Coumarin derivatives, see **Figure 66b** (Coumarin derivatives with thiosemicarbazone (**103**, b) group in the 3-position).

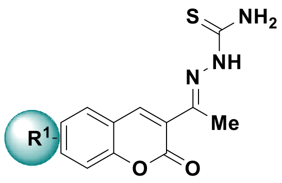

103

Compound	R^1	IC_{50} (μM)
**103a**	H	4.0
**103b**	7,8-diOH	4.1
**103c**	7-OH	5.0
**103d**	8-OH	5.7
**103e**	6,7-diOH	6
**103f**	6-OH	14
kojic acid (**5**)		18.1

Table S62. Coumarin derivatives, see **Figure 67** (Coumarin-based thiophenyl-pyrazolylthiazole hybrids which show good activity in TYR inhibition.).

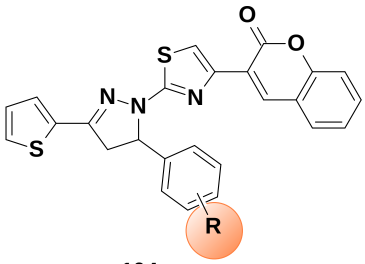

104

Compound	R	IC_{50} (μM)
**104a**	3-OMe,4-OBn	0.043
**104b**	4-OMe	0.278
**104c**	4-Br	0.809

**104d**	H	1.206
**104e**	4-F	1.946
**104f**	4-Cl	2.028
**104g**	4-Me	4.071
**104h**	4-OPh	4.508
kojic acid (**5**)		18.521

Table S63. Kojic acid derivatives, see **Figure 68** (Triazole-based kojic acid derivatives **107** and their inhibitory profile on TYR.).

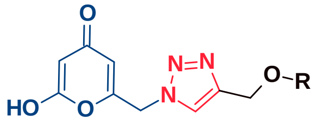

107

Compound	R	IC_{50} (µM)
**107a**	1-naphtyl	0.06
**107b**	4-hydroxycoumarin	0.02
**107c**	2-naphtyl	0.30
**107d**	4-NO_2-Ph	0.52
**107e**	4-Cl-Ph	0.69
**107f**	3,5-diMe-Ph	0.74
**107g**	3,4,5-*tri*OMe-Ph	0.87
**107h**	4-F-Ph	0.88
**107i**	3-Cl-Ph	0.99
**107j**	3-F-Ph	1.07
**107k**	2,4-diCl	1.12
**107l**	4-OMe-Ph	1.24
**107m**	4-CH_2OH-Ph	1.32
**107n**	Ph	1.33
**107o**	4-$CH_3CONHPh$	2.64
**107p**	4-CN-Ph	6.29
kojic acid (**5**)		9.28

Table S64. Kojic acid derivatives, see **Figure 69** (Kojic acid derivatives containing a benzylpiperazine portion (**108**) and their TYR inhibition).

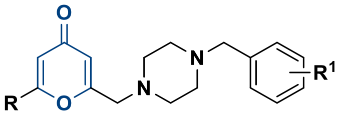

108

Compound	R	R^1	IC_{50} (µM)
**108a**	hydroxymethyl	3,4-diCl	86.2
**108b**	pirrolidinylmethyl	3,4-diCl	94.1
**108c**	methyl	3,4-diCl	123.7
**108d**	methyl	3-Cl	198.7
**108e**	piperidinylmethyl	3,4-diCl	204.1
**108f**	hydroxymethyl	3-Cl	223.2
**108g**	pirrolidinylmethyl	4-F	225.5

**108h**	piperidinylmethyl	4-F	232.3
**108i**	hydroxymethyl	4-F	264.2
**108j**	methyl	4-F	362.1
**108k**	methyl	4-Cl	438.8
**108l**	piperidinylmethyl	3-Cl	493.8
**108m**	pirrolidinylmethyl	3-Cl	588.2
**108n**	chloromethyl	4-F	618.6
**108o**	hydroxymethyl	4-Cl	710.0
**108p**	piperidinylmethyl	4-Cl	746.0
**108q**	chloromethyl	3-Cl	990.7
**108r**	morpholinylmethyl	3-Cl	1105
**108s**	hydroxymethyl	2,5-diF	1564
**108t**	pirrolidinylmethyl	2,5-diF	1616
**108u**	morpholinylmethyl	4-Cl	1759
**108v**	morpholinylmethyl	4-F	1847
**108w**	morpholinylmethyl	2,5-diF	1900
**108x**	chloromethyl	4-Cl	1900
**108y**	chloromethyl	2,5-dF	>2000
**108z**	morpholinylmethyl	3,4-diCl	>2000
**108aa**	piperidinylmethyl	2,5-dF	>2000
kojic acid (**5**)			418.2

Table S65. Kojic acid derivatives, see **Figure 70** (Thioquinazolinones conjugated to kojic acid derivatives (**109**) showing medium 3-digit nanomolar activity as TYRIs).

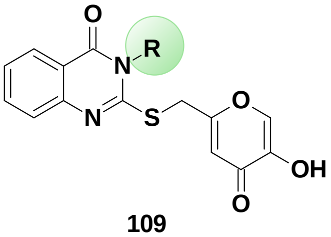

109

Compound	R	IC_{50} (μM)
**109a**	pyridin-2-ylmethyl	0.46
**109b**	pyridin-2-yl	0.50
**109c**	4-Cl-Ph	0.50
**109d**	Bn	1.42
**109e**	*i*-Pr	1.44
**109f**	4-Br-Ph	1.48
**109g**	4-F-Bn	1.56
**109h**	allyl	1.56
**109i**	Ph	1.88
**109j**	cyclopropyl	2.10
**109k**	3,4-diOMe-Ph	2.12
**109l**	pyridin-4-ylmethyl	2.20
**109m**	*n*-butyl	2.31
**109n**	4-OMe-Ph	2.40
**109o**	4-Me-Ph	5.32
kojic acid (**5**)		9.30

Table S66. Kojic acid derivatives, see **Figure 71a** (Kojic acid derivatives).

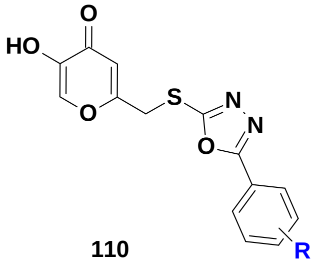

110

Compound	R	IC_{50} (μM)
**110a**	4-F	5.32
**110b**	4-Me	6.17
**110c**	4-OMe	9.57
**110d**	Ph	10.24
**110e**	3-Cl	10.24
**110f**	3,4-diOH	10.67
**110g**	4-*t*Bu	11.34
**110h**	4-Br	11.47
**110i**	4-Cl	11.88
**110j**	4-OH	14.43
**110k**	2-OH	14.79
**110l**	3-Me	17.36
**110m**	3-OH	19.30
**110n**	2-NO_2	19.45
kojic acid (**5**)		49.77

Table S67. Kojic acid derivatives, see **Figure 71b** (Kojic acid derivatives).

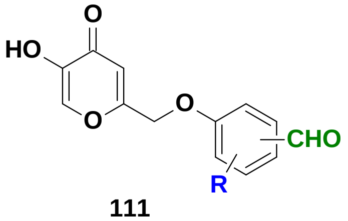

111

Compound	R	CHO	IC_{50} (μM)
**111a**	3-F	*para*	5.32
**111b**	H	*para*	5.98
**111c**	2-F	*para*	9.78
**111d**	3,5-diMe	*para*	12.39
**111e**	2-Cl	*para*	12.75
**111f**	5-OMe	*meta*	25.31
**111g**	2-OMe	*para*	26.44
**111h**	2-C_2H_5O	*para*	37.34
**111i**	2,6-diMe	*para*	77.18
**111j**	2-OMe,6-Br	*para*	77.89
**111k**	2,6-diOMe	*para*	>100
**111l**	6-OMe	*ortho*	>100
**111m**	6-C_2H_5O	*ortho*	>100
kojic acid (**5**)			48.05

Table S68. Human tyrosinase inhibitors, see **Figure 73** (PROTACs as human tyrosinase degraders).

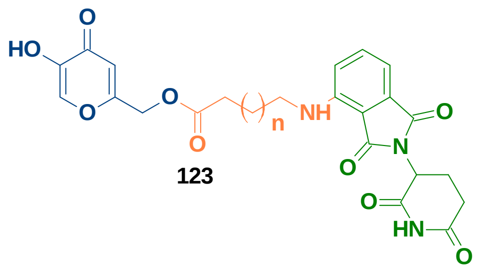

123

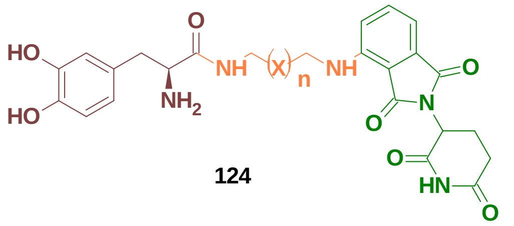

124

Compound	*n*	X	Reduction (%)	IC_{50} (µM)
**123a**	3	-	-	-
**123b**	5	-	-	-
**123c**	6	-	-	-
**123d**	8	-	-	-
**124a**	3	CH_2	46	112.7
**124b**	6	CH_2	38	-
**124c**	5	CH_2	37	-
**124d**	8	CH_2	37	-
**124e**	4	CH_2	33	-
**124f**	1	CH_2	25	-
**124g**	2	CH_2	19	-
**124h**	0	CH_2	14	-
**124i**	1	CH_2-O-CH_2	7	-
**124j**	2	CH_2-O-CH_2	7	-

Table S69. Human tyrosinase inhibitors, see **Figure 75** (Resorcinol-based hemiindigoid derivatives).

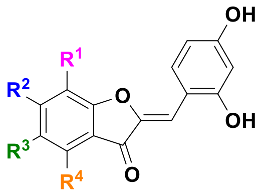

125

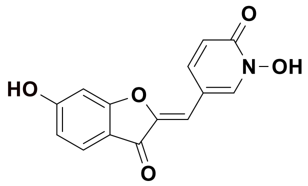

125f

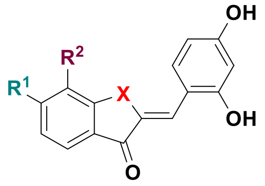

126

Compound	R^1	R^2	R^3	R^4	X	MNT-1 lysate IC_{50} (µM)	MNT-1 whole cells IC_{50} (µM)	MNT-1 whole cells CC_{50} (µM)
**125a**	H	H	H	OH	-	1.57	32	108
**125b**	H	OH	H	H	-	6.4	-	-
**125c**	H	OMe	H	H	-	6.9	-	-
**125d**	H	OH	H	OH	-	7.7	-	-
**125e**	OH	OH	H	H	-	11.8	-	-
**125f**	-	-	-	-	-	15.0	-	-
**125g**	H	H	H	H	-	18.0	-	-
**125h**	H	$N(Me)_2$	H	OH	-	18.4	-	-
**125i**	H	H	OH	H	-	19.0	-	-
**125j**	H	H	H	OH	-	25.0	-	-
**125k**	H	I	OH	H	H	-	32.0	-	-
**125l**	Me	OH	H	H	-	80.0	-	-

**125m**	OMe	H	H	H	-	80.0	-	-
**125n**	H	F	H	F	-	88.0	-	-
**125o**	H	OBu	H	H	-	>100	-	-
**125p**	H	Cl	H	H	-	>100	-	-
**125q**	H	$N(Me)_2$	H	H	-	>100	-	-
**125r**	H	OMe	H	OMe	-	>100	-	-
**125s**	Me	OMe	-	-	-	>100	-	-
**125t**	OMe	OMe	-	-	-	>100	-	-
**126a**	H	OH	-	-	CH_2	1.6	29	91
**126b**	OH	H	-	-	NH	2.3	-	-
**126c**	OH	H	-	-	CH_2	3.4	-	-
**126d**	H	H	-	-	CH_2	5.8	-	-
**126e**	OH	H	-	-	S	5.8	-	-
**126f**	H	H	-	-	S	5.9	-	-
**126g**	H	H	-	-	CHMe	8.2	-	-
**126h**	H	H	-	-	NH	12.7	-	-
**126i**	H	OH	-	-	S	48.0	-	-
**126j**	H	H	-	-	C=O	>100	-	-
rucinol **(9)**						8.3	-	-
